# Mcm5 mutation leads to silencing of Stat1-bcl2 which accelerating apoptosis of immature T lymphocytes with DNA damage

**DOI:** 10.1038/s41419-025-07392-8

**Published:** 2025-02-10

**Authors:** Min Liu, Yuanyuan Li, Zhilin Deng, Ke Zhang, Shuying Huang, Jiamin Xia, Yi Feng, Yundan Liang, Chengfu Sun, Xindong Liu, Shurong Li, Bingyin Su, Yong dong, Sizhou Huang

**Affiliations:** 1 Development and Regeneration Key Laboratory of Sichuan Province, Department of Anatomy and Histology and Embryology, School of Basic Medical Sciences, Chengdu Medical College, Chengdu, 610500 China; 2 Department of Neurology, the Second Affiliated Hospital of Chengdu Medical College, Nuclear Industry 416 Hospital, Chengdu, 610000 China; 3 Centre for Inflammation Research, Queen’s Medical Research Institute, Institute for Regeneration and Repair, The University of Edinburgh, Edinburgh, UK; 4 Department of Pathology and Pathophysiology, Chengdu Medical College, Chengdu, 610500 China; 5 Department of Immunology, School of Basic Medical Sciences, Chengdu Medical College, Chengdu, 610500 China; 6 Present Address: Department of Cardiology, The First Affiliated Hospital, Chengdu Medical College, Chengdu, 610500 Sichuan China

**Keywords:** Lymphopoiesis, T cells

## Abstract

Mutation in genes involved in DNA replication continuously disrupt DNA replication and give rise to genomic instability, a critical driver of oncogenesis. To prevent leukemia, immature T lymphocytes with genomic instability often undergo rapid cell death during development. However, the mechanism by which immature T lymphocytes undergo rapid cell death upon genomic instability has been enigmatic. Here we show that zebrafish *mcm5* mutation leads to DNA damage in immature T lymphocytes and the immature T cells sensitively undergo rapid cell death. Detailed analyses demonstrated that the immature T lymphocytes undergo rapid apoptosis via upregulation of *tp53* and downregulation of *bcl2* transcription in *mcm5* mutants. Mechanistically, Mcm5 directly binds to Stat1a and facilitates its phosphorylation to enhance *bcl2a* expression under the conditions of DNA replication stress. However, in *mcm5* mutants, the absence of the Mcm5-Stat1 complex decreases Stat1 phosphorylation and subsequent *bcl2a* transcription, accelerating apoptosis of immature T lymphocytes with genomic instability. Furthermore, our study shows that the role of Mcm5 in T-cell development is conserved in mice. In conclusion, our work identifies a role of Mcm5 in regulating T cell development via Stat1-Bcl2 cascade besides its role in DNA replication, providing a kind of mechanism by which immature T cells with gene mutation-induced DNA damage are rapidly cleared during T lymphocyte development.

## Introduction

Genomic instability drives the initiation, metastasis, and progression of T cell acute lymphoblastic leukemia (T-ALL) [1, 2]. During tumorigenesis, genomic instability resulting from p16INK4A deletion [3], mutations in mini-chromosome maintenance proteins MCM4/2 [4–6] and impaired nonhomologous end joining (NHEJ) [7] acts cooperatively with MYC to increase cell proliferation, drive tumor initiation and expand early transformed cells. Although promotion of myeloid differentiation has been reported to inhibit leukemic self-renewal and malignant hematopoiesis in myeloid stem cells [8], efficient clearance of lymphocytes with genomic defects is a common way to prevent lymphoblastic leukemia [9–11]. This principle is also supported by the observation that inhibition of apoptosis in p53 mutant cells or overexpression of antiapoptotic signaling mediators drives leukemia [12–16], including T-ALL [13]. Therefore, during T lymphocyte development, immature lymphocytes appear to sensitively undergo cell death after undergoing DNA damage caused by gene mutations (chronic DNA replication stress) [6, 17, 18], while the surviving lymphocytes may progress to thymomas at later developmental stages [6, 19, 20]. On the other hand, DNA damage arises from both endogenous gene mutations and transient exposure to environmental stress [21, 22]. When DNA damage is caused by transient replication stress, anti-apoptotic signaling is activated to prevent rapid cell death to help preserve life and evolution [20, 23]. Thus, organisms must evolve mechanisms to differentiate chronic DNA damage induced by gene mutations from transient DNA replication stress. Using such mechanisms, organisms can eliminate lymphocytes with severe and unrepaired DNA damage to maintain a stable genome and prevent leukemia, preserve life and enhance evolution under transient environmental replication stress. However, these mechanisms are far from being elucidated.

Early studies have shown that the Mcm4(D573H) allele destabilizes the MCM2–7 complex, resulting in chromosome instability and the formation of spontaneous T cell lymphoblastic leukemia/lymphoma (T-ALL) [6]. Mice with reduced expression of MCM2 due to an MCM2 mutant allele die with lymphoma within the first few months after birth [5]. MCM5 is also a crucial component of the DNA replication licensing system (MCM2-7 complex), and in Steere’s early screening work, a MCM5 variant allele was identified with pathogenic potential [24]. MCM5 is highly expressed in T cells from patients with T-ALL as well as in T-ALL cell lines (Fig. S1A-C Fig. S1B Gel Supplementary). These findings imply that MCM5 may play a pivotal role in T lymphocytic leukemia and early T cell development. Here, we focused on studying the role of Mcm5 in T cell development and found that in mice and zebrafish, Mcm5 loss disrupted DNA replication in immature T lymphocytes and sequentially led to upregulation of Tp53 signaling, which resulted in apoptosis of immature T lymphocytes. Moreover, in *mcm5* mutants, the absence of *mcm5* inhibited the phosphorylation of Stat1 and sequentially inhibited the enhancement of *bcl2* signaling, which accelerated the apoptosis of immature T lymphocytes. Our results provide a mechanism by which immature T cells with DNA damage resulting from gene mutations are rapidly cleared during T-cell development.

## Material and Methods

### Mice and fish maintenance

Wild-type (AB, WT) animals and *Tg(c-myb:EGFP)* [25], *Tg(coro1a:EGFP)* [26], *Tg(gata1:DsRed)* [27], *Tg(lyz:DsRed)* [26]*, Δ113p53*^*M/M*^ [28], *mcm5*^*+/−*^ [29]*, mcm3*^*+/−*^*, Tg(HSP70l:mcm5-t2a-mCherry)* and *Tg(Rag2:DsRed)* [30] transgenic lines were maintained under standard conditions at approximately 28.5 °C. The developmental stages were characterized as previously described [31]. Mcm5^fl/fl^ (Strain S-CKO-03682) mice were purchased from Cyagen Co., Ltd, and Mx1-cre (B6.Cg-Tg(Mx1-cre)1Cgn/J) was a gift from Cyagen Co., Ltd.

### Ethics statement

The study was approved by the Institutional Review Board of Chengdu Medical College (SYXK(川)2015-196). All mice were maintained under specific pathogen-free conditions by the Animal Center of Chengdu Medical College, and all procedures were approved by the Institutional Ethics Review Committee of Chengdu Medical College (CMC-IACUC-2022050).

### MCM3 mutant construction

One sgRNA was designed based on exon 4 of the *mcm3* genomic DNA, and the sgRNA was synthesized in vitro with a HiScribe™ T7 High Yield RNA Synthesis Kit (NEB, No. E2040S) according to the manufacturer’s instructions. The detailed procedure was performed based on previous reports [30]. An additional “A” nucleotide was inserted into the genomic DNA sequence of *mcm3* gene, resulting in a premature stop codon in the CDS region of *mcm3* that corresponded to translation termination at amino acid 178.

### Cas9-sgRNA ribonucleoprotein complex (RNP) preparation and injection

To generate the mosaic mutation in F0 embryos, we optimized the CRISPR-Cas9 gene editing process according to previous reports [32]. In brief, four or three forward primers (targeting *bcl2a* or GFP) and the reverse primer were designed using an online tool (https://www.crisprscan.org/?page=gene), and the sgRNAs were then synthesized in vitro using a HiScribe™ T7 High Yield RNA Synthesis Kit (NEB, E2040S). The Cas9 protein (EnGen^®^ Spy Cas9 NLS, NEB, M0646T) was purchased from NEB. To examine the mutation efficiency of *bcl2a*, the injected embryos were collected, and genomic DNA was prepared and analyzed by semi-quantitative RT‒PCR as described in a previous report [31].

### Plasmid construction

Total RNA was extracted according to the manufacturer’s instructions using TRIzol (TRIzol, Ambion, 15596 - 026). cDNA was prepared using Revert Aid First Strand cDNA Synthesis Kit (Fermentas, K1622) according to the manufacturer’s instructions. The coding sequences (CDS) of *Δ113p53* and *bcl2a* were individually amplified by PCR (Prim STAR Max Premix Takara,R045A) and cloned into the vector PCS^2+^ to generate the expression constructs (5x In-Fusion HD Enzemy Premix, Takara, 639649). To the plasmids used in CoIP experiments, the CDS of zebrafish *mcm5* or *stat1a* were amplified by PCR respectively, and cloned into the pcDNA3.1^+^ vector which contained Flag-tag or His-tag. All primers used for cloning are listed in Table S2.

### Mouse bone marrow competitive transplantation and flow cytometry analysis

In this experiment, two mouse lines were used, Mcm5f/f (CD45.2) and Mx1-Cre (CD45.2) mouse. In the Mx1-Cre transgenic mouse, the Cre is under control of the interferon-gamma promoter [33]. To analyze the impact of Mcm5 knockout on mouse hematopoiesis, we performed competitive transplantation of Mcm5f/f;Mx1-Cre (CD45.2) and WT (CD45.1) mouse bone marrow by transplanting total bone marrow into lethal irradiation-treated recipients at a ratio of 1:1 (0.25 million Mcm5f/f;Mx1-Cre (CD45.2) nucleated cells and 0.25 million WT (CD45.1) nucleated cells). Polyinosinic polycytidylic acid (pI-pC) could be used to induce the expression of Cre controlled by the promoter of Mx1 [34]. To induce the knockout of Mcm5 in hematopoietic cells on 4 weeks after transplantation, 200 µg of polyinosinic-polycytidylic acid (pI-pC; Sigma Aldrich) was injected intraperitoneally into all recipients every other day for 3 doses [35]. The peripheral blood (PB) cells of recipients were detected at 4 weeks, 8 weeks and 11 weeks with PE anti-mouse CD45.1 (A20) and FITC anti-mouse CD45.2 antibodies (104). Hematopoietic stem cells/multipotent blood progenitors (HSCs/MPPs) were detected with FITC anti-mouse CD2 (RM2-5)/CD3 (145-2C11)/CD4 (GK1.5)/CD8 (53-6.7)/Ter119 (TER-119)/CD11b (M1/70)/B220 (RA3-6B2)/Gr-1 (RB6-8C5)/CD48 (HM48-1), APC anti-mouse CD117 (c-kit) (2B8), PerCP/Cyanine5.5 anti-mouse Ly-6A/E (Sca-1) (D7), and PE/Cyanine7 anti-mouse CD150 (SLAM) (TC15-12F12.2); common lymphocyte progenitors (CLPs) were detected with PE/Cyanine7 anti-mouse CD127 (IL-7Rα) (SB/199), FITC anti-mouseCD3 (145-2C11)/CD4 (GK1.5)/CD8 (53-6.7)/Ter119 (TER-119)/CD11b (M1/70)/B220 (RA3-6B2)/Gr-1 (RB6-8C5)/CD48 (HM48-1), APC anti-mouse CD117 (c-kit) (2B8), and PerCP/Cyanine5.5 anti-mouse Ly-6A/E (Sca-1) (D7); and T-lineage cells were detected with PE/Cyanine7 anti-mouse CD4 (GK1.5), APC anti-mouse CD8a (53-6.7), FITC anti-mouse CD25 (PC61), and PerCP/Cyanine5.5 anti-mouse/human CD44 (IM7). All antibodies were purchased from BioLegend, Inc. All samples were analyzed using a FACSCantoII instrument (BD Biosciences). The flow cytometry analysis data were analyzed using FlowJov10 software (FlowJo).

### Chemical treatment

As previously reported, zebrafish embryos were treated with 25 nM camptothecin (Beyotime, SC0141), 2.5 μM roscovitine (Sigma, R7772), and 75 μM aphidicolin, as described in previous studies [23, 36]. In brief, the chemicals were diluted in egg water to the concentrations mentioned above, and the embryos were incubated with the chemicals from 1.5 dpf or 3 dpf until the required developmental stage.

### Morpholino oligonucleotide (MO) and mRNA injection

MOs for *mcm5* [37], *p53* [38] and *stat1a* [39] and the control MO were obtained from Gene Tools. *mcm5* mRNA, *Δ113p53* mRNA and *bcl2a* mRNA were synthesized in vitro using an mMESSAGE Kit (Ambion, AM1340). The concentrations of the MOs were as follows: *mcm5* MO, 300 µM; *p53* MO, 200 µM; *stat1a*MO, 500 μM; and control MO, 500 μM. The concentration for mRNA injection was as follows: *mcm5* mRNA, 30 ng/μl; *bcl2a* mRNA, 30 ng/μl; *Δ113p53* mRNA, 30 ng/μl. All MOs and mRNAs were injected at the 1- to 4-cell stage.

### RNA sequencing (RNA-seq) and RT‒qPCR

Total RNA from *mcm5* mutants and WT embryos was extracted using TRIzol reagent (Invitrogen, CA, United States; 15596026). RNA-seq and analysis were performed by Novogene Co. Ltd. (TianJin, China). The sequencing reads were mapped to the Ensemble zebrafish reference genome (GRCz11) using STAR alignment software. Differential gene expression analysis was performed with DESeq2. RT‒qPCR was performed using Brilliant III Ultra-Fast SYBR Green QPCR Master Mix (Agilent Technologies) and a CFX96 Real-Time System (Bio-Rad) according to the manufacturers’ instructions. *Beta-actin* was used for normalization. The primers are listed in Table S1. All experiments were repeated at least 3 times.

### Whole-mount in situ hybridization (WISH)

In situ hybridization was performed as described previously [31]. The previously designed *mcm5, rag2, rag1, foxn1, ccl25a, ikaros, scl, hbbe3, gata1, lmo2, pu.1, c-myb, il7r* and *GH* probes were used as described in previous reports [30, 31, 40]. The CDSs of *mcm3, bcl2a* and the specific region of *Δ113p53* were amplified using PCR and inserted into the vector pcs2^+^. Then, the plasmids were linearized, and the individual antisense probes were synthesized. To synthesize the *p53*-specific probe, the CDS of *p53* was amplified using PCR (the Sp6 promoter sequence was added to the 3’ end of the reverse primer); part of the PCR product was used for sequencing to evaluate whether the correct PCR product was obtained, and the remaining PCR product was used as the template to synthesize antisense probes [31].

### O-dianisidine staining

O-dianisidine staining (Aladdin, 119-90-4) was carried out as previously reported [41, 42]. The stained embryos were stored at 4 °C in the dark for photography.

### Western blotting analysis and cell transfection

To measure the protein levels of Tp53 (GeneTex, GTX128135, which specifically recognizes the N-terminal of Tp53), γH2AX (GeneTex, GTX127340), BCL2a (Abcam, ab182858), Stat1 (Santa Cruz, SC-464) and p-Stat1 (Santa Cruz, SC-8394) in embryos subjected to different treatments, approximately 50–100 embryos were collected for protein extraction. Western blotting was performed as described previously [43]. 293 T cells were cultured to 80%-90% confluence in 6-well plates. The transfection reagent (Invitrogen, L3000001) and the siRNA (purchased from RiboBio) or plasmid were mixed in EP tubes and placed at room temperature for 10–15 min before being added to 6-well plates. The transfection efficiency was evaluated by Western blotting at 72 hours after transfection.

### Co-immunoprecipitation (Co-IP)

Co-IP was performed as described previously [44]. 293 T cell line is a gift from Dr. Tai Yang in Chengdu medical college. Briefly, 293 T cells were co-transfected with Mcm5-Flag and Stat1a-HA plasmids, Mcm5-Flag and PEGFP plasmids. After 72 h, the medium was removed and the cell was washed twice with pre-cooled PBS. 1 ML NP-40 lysate was added into the 10 cm cell culture dish, then the adherent cells were scraped off with cell curettage, and cells were collected into the EP tube. Leave it on ice for 20 min, then centrifuge (12,000 x g for 15 min) and remove the supernatant into EP tube, centrifuge the supernatant again. Prepare 50 μ L supernatant as input, add 5X Loading buffer and mix completely, boil it at 100 °C for 7 min, then store it in −20 °C for future work. When carrying out the CoIP experiment, add 4 μg antibody (Anti-mouse-Flag, Sigma, F1804) to the remaining samples and incubate them overnight at 4 °C. Then prepare new tube to rinse 70 μ L protein G (Invitrogen, 10003D) with 1XPBS, centrifuge at 2000rpm for 5 min, discard the supernatant and get the rinsed protein G. Next the protein G was added into the samples and incubated at 4 °C overnight. Then centrifuge it for 5 min at 4 °C (2000rpm) and discard the supernatant, wash the precipitate 4–5 times with 1 mL NP-40 buffer for 15 min each time. Finally add 2X Loading buffer into the precipitate, boil the mix at 100 °C for 7 min, centrifuge at 2000 rpm for 5 min, collect the supernatant and store at −20 °C for Western Blotting detection. The primary antibody Anti-rabbit-HA (abcam, AB236632) and anti-GFP (Cloud Clone, PAD025Ge07) was used in this research.

### Immunostaining

Embryos were fixed overnight with PEM at 4 °C, washed with PBS (3 times for 5 min each) and blocked with PBTN (4% BSA and 0.02% NaN_3_ in PT) for 2 h at 4 °C. Then, primary antibodies against H3p (GTX128116) and γH2AX (GeneTex, GTX127340) were diluted with PBTN at a ratio of 1:100 and incubated on a shaker at 4 °C overnight. Then, the embryos were washed with PT (0.3% Triton-X-100 in1X PBS) 8 times for at least 20 min each. The secondary antibody (GeneTex, 26800) was diluted 1:500 with PBTN and added. The embryos were incubated overnight at 4 °C (in the dark). Finally, the embryos were washed with PT at least 8 times (30 min each time) and imaged.

### EdU incorporation assay

EdU staining was performed as described in previous reports [45]. A BeyoClick™ EdU Cell Proliferation Kit with Alexa Fluor 594 (Beyotime, C0078S) was used for this experiment. After EdU staining, the embryos were washed with PT 3 times prior to immunostaining for GFP.

### Staining of apoptotic cells

Embryos were dechorionated, fixed with 4% paraformaldehyde overnight at 4 °C, washed with PBST 3 times (10 min each time) and stored in 100% methanol overnight. Then, the embryos were washed 3 times with PBST, and an In Situ Cell Death Detection Fluorescein Kit (Roche11684795910) was applied to examine apoptosis according to the manufacturer’s instructions.

### Statistical analysis

The data were analyzed with NovoExpress, ImageJ, and GraphPad Prism 8 for Windows statistical software (GraphPad Software). Welch’s t-test (two-tailed, unequal variance) was performed to determine the statistical significance of the differences. Quantitative data are presented as the mean ± SD values. Each experiment was performed at least three times. The individuals in each group were selected randomly. The fish sample size is more than 9 individuals, the mouse sample size is more than 3 individuals. All the sample size was showed in the figures and figure legends. NS, not significant; “*”, *p* < 0.05; “**”, *p* < 0.01; and “***”, *p* < 0.001.

## Results

### mcm5 regulates T lymphocyte maturation during definitive hematopoiesis

**Fig. 1:**
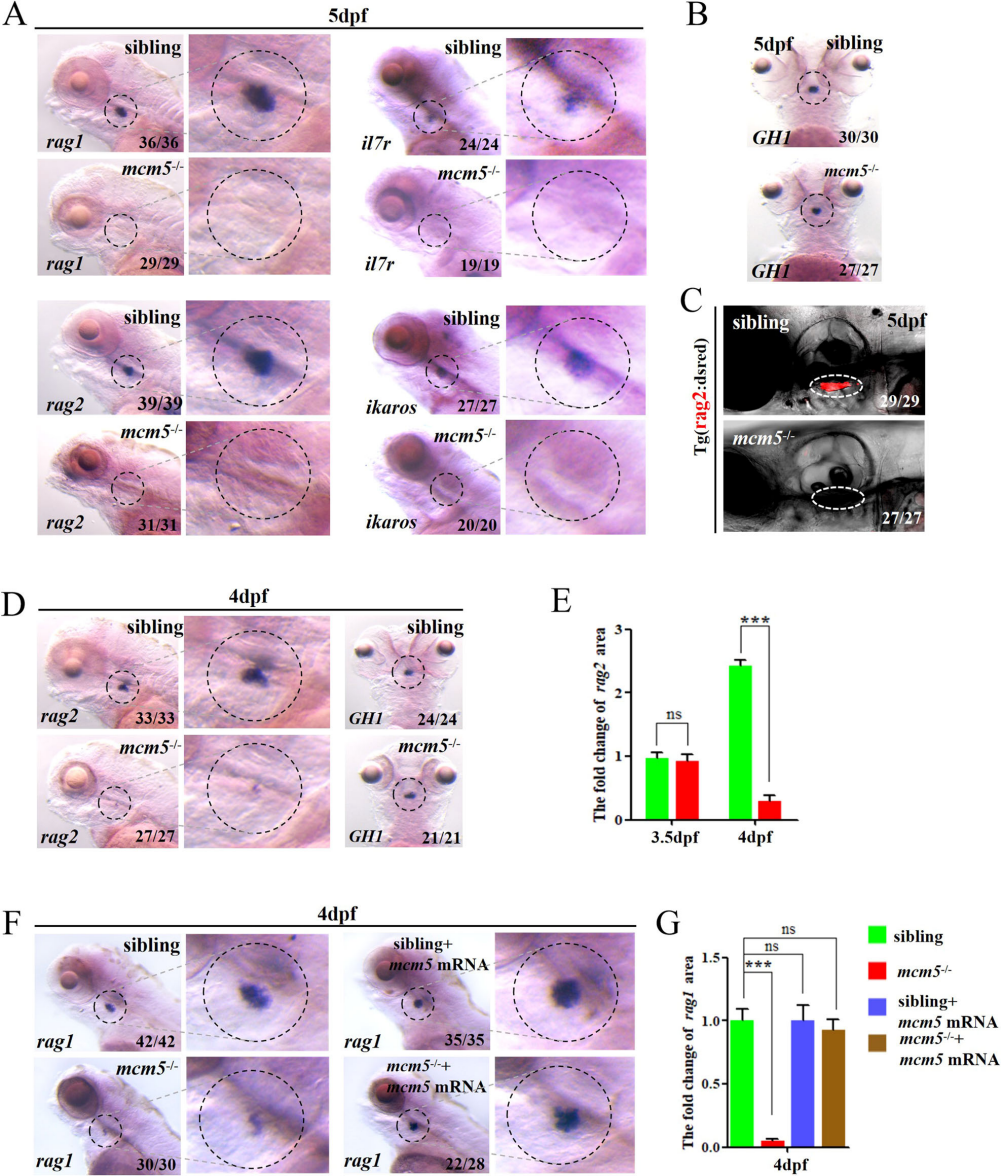
*Mcm5* regulates T lymphocyte maturation during hematopoiesis. **A** In control sibling embryos, the expression of *rag1*, *rag2*, *il7r* and *ikaros* was examined at 5 dpf (shown in the upper-row images). In *mcm5*^*−/−*^ embryos, the expression of *rag1*(n = 29), *rag2* (*n* = 31)*, il7r* (*n* = 19) and *ikaros* (*n* = 20) completely disappeared at 5 dpf (shown in the bottom-row images). **B** *GH1* is a pituitary maker, here working as a control. Comparing to siblings (*n* = 30), the expression of *GH1* in mcm5 mutants was normal (*n* = 27). **C** The cells labeled with DsRed (in *Tg*(rag2:DsRed)) disappeared in *mcm5*^*−/−*^ embryos at 5 dpf (*n* = 27). **D** Expression of *rag2* was greatly decreased in *mcm5*^*−/−*^ embryos at 4 dpf (**D**, *n* = 27). The expression of *GH1* was normal in *mcm5*^*−/−*^embryos at 4 dpf (**D**, *n* = 21). **E** Quantification of the expression area of *rag2* in siblings and *mcm5*^*−/−*^ embryos at 3.5 dpf and 4 dpf was showed. **F** The expression of *rag1* in *mcm5*^*−/−*^ embryos was significantly downregulated at 4 dpf in the thymus (*n* = 30). *Mcm5* mRNA injection restored the expression of *rag1* in *mcm5*^*−/−*^ embryos (*n* = 28). **G** The quantification of the expression area of *rag1* at 4 dpf is showed. For (**E,G**), the data were presented as means ± SD; The *P*-values (t-test; two-tailed); *Ns* not significant; *** *P* < 0.001.

MCM5 is a key component of the DNA replication licensing system. In zebrafish, *mcm5* is highly expressed in all proliferating cells [37], including hematopoietic stem cells (HSCs) and T cells in the thymic region (Fig. S2). This data implied a potential role for *mcm5* in T cell development. To investigate T-cell development under genomic instability induced by endogenous DNA replication stress, a premature stop codon mutation in *mcm5* was generated ([29] and Fig. S3). As previously reported [29, 37], *mcm5*^**−/−**^ embryos exhibited shorter body lengths, smaller retinas and heads than WT embryos and died at approximately 9 days post fertilization (dpf). During the primitive hematopoiesis stage, HSC markers (*scl* and *c-myb*), erythrocyte markers (*gata1* and *hbbe3*), and myelopoiesis regulators (*lmo2* and *pu.1*) were analyzed. The results indicated that primitive hematopoiesis remained intact in *mcm5*^**−/−**^ embryos and *mcm5* morphants (Fig. S4A–F) [38]. In the secondary wave of hematopoiesis, the expression of the T-cell markers *rag1*, *il7r*, *ikaros*, and *rag2* was absent at 5 dpf (Fig. 1A–C), whereas erythrogenesis was largely unaffected (Fig. S5A, B). Furthermore, the data showed that *rag2* expression remained intact at 3.5 dpf in *mcm5* mutant embryos (Fig. S6A) but was significantly downregulated by 4 dpf (Fig. 1D, E). These data suggested that *mcm5* plays a critical role in T-cell maturation but not in early events during T-cell development.

Since defects in the thymic epithelium, HSC specification and HSC migration can all impair T lymphocyte development [40], to evaluate whether these processes were affected in *mcm5* mutants, we examined the expression of the thymus markers *foxn1* and *ccl25a*, as well as the HSC marker *c-myb*. The results indicated that the area of *ccl25a* and *foxn1* expression was reduced in *mcm5*^**−/−**^ embryos (appearing more condensed, Fig. S6B–F), although the expression levels were only mildly diminished (Fig. S6G), implying *mcm5* loss of function partially disturbed thymus development, which contributed, at least partially, to the defect in T-cell maturation in *mcm5*^**−/−**^ embryos. However, the results demonstrated that HSC specification (Fig. S7A, C) and migration (Fig. S7B, D) were unaffected in *mcm5*^**−/−**^ embryos. These data above further showed that *mcm5* mainly regulates T cell maturation, while it has a limited role in the early stages of T-cell development.

To further confirm the role of *mcm5* in T cell maturation, over expression of *mcm5* mRNA was applied to rescue T cell developmental defect in *mcm5* mutants. The data showed that injecting *mcm5* mRNA at the 1- to 4-cell stage rescued the T cell developmental defect (Fig. 1F, G and Fig. S6H). In addition, specific expression of *mcm5* in T cells by injecting TolII mRNA and Rag2:mcm5-T2A-mCherry plasmid also partially rescued T cell developmental defect (Fig. S6I, J), implying *mcm5* cell-autonomously regulates T cell maturation. Collectively, these findings further demonstrated that *mcm5* mutation specifically impacts T lymphocyte maturation, while progenitor specification and migration during definitive hematopoiesis remain unaffected.

### Immature T cells in *mcm5*^−/−^ embryos exhibit increased apoptosis

**Fig. 2:**
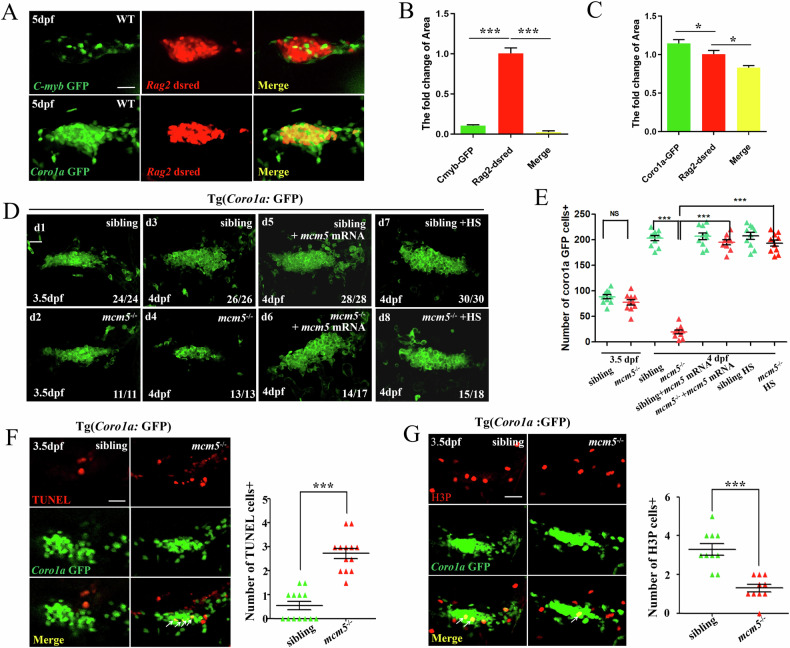
Immature T lymphocytes undergo cell death in *mcm5*^*−/−*^ embryos. **A**–**C** Analysis of the expression of *Rag2*:DsRed, *C-myb*:GFP and *Coro1a*:GFP at 5 dpf. The expression of *C-myb*:GFP almost did not overlap with *Rag2*:DsRed expression (**A**, **B**, yellow column, *n* = 3, 3%), and most *Rag2*:DsRed-positive cells overlapped with cells labeled with *Coro1a*:GFP (A, C, yellow column, *n* = 3, 82.4%). **D**, **E** No significant difference was observed for *Coro1a*:GFP cells in siblings (d1, *n* = 24) and *mcm5*^*−/−*^ embryos (d2, *n* = 11) at 3.5 dpf (d1-d2, E; *n* = 11), but at 4 dpf, the number of *Coro1a*:GFP cells was greatly decreased (d3-d4, E; *n* = 13). This phenotype can be rescued by injecting *mcm5* mRNA at the 1- to 4-cell stage (d5-d6, E; *n* = 17) or heat shock-inducing *mcm5* expression at 3 dpf (d7-d8, E; *n* = 18). Here we counted the *Coro1a*:GFP labeled cells in each single slice, then added them together as the whole T cell number. **F** TUNEL-positive *Coro1a*:GFP cells were increased in *mcm5*^*−/−*^ embryos (*n* = 13) compared with their siblings (*n* = 13). **G** There were fewer H3P-positive *Coro1a*:GFP cells in *mcm5*^*−/−*^ embryos (*n* = 10). Here H3P is a mitotic marker Histone H3 phosphorylated at Serine 10. Scale bars, 40 μm. Statistical analyses of the expression of *Coro1a*:GFP are shown on the right. For (**B**, **C**, **E**–**G**), the data were presented as means ± SD; The *P*-values (t-test; two-tailed); NS, not significant. “***” *P* < 0.001.

Next, we continued to study the detailed role of *mcm5* in zebrafish T cell maturation. Given the facts that in *mcm5* mutant embryos, T cells were substantially reduced at 4dpf and not detected at 5dpf (Fig. 1), the DsRed was not observed expressing on 3.5dpf in *Tg(Rag2:DsRed)* transgenic line, we could not use *Tg(Rag2:DsRed)* transgenic line and tried to find other transgenic line to observe T cell developmental process before T cell death in *mcm5* mutants. In transgenic *Tg(c-myb:GFP)* embryos, only HSCs, not immature T lymphocytes, were labeled with GFP at 4 dpf (Fig. 2A, B and Fig. S8A). In contrast, in *Tg(coro1a:GFP)* embryos, GFP-labeled cells marked immature T cells [26]. Detailed analysis revealed that these cells were predominantly colocalized with immature T lymphocytes in the thymic epithelial area (Fig. 2A, C and Fig. S8B). Additionally, from 3.2 dpf to 3.6 dpf, GFP- labeled cells were observed in the thymic region (Fig. S9A-C). Therefore, we utilized *Tg(coro1a:GFP)*:*mcm5*^*−/−*^ embryos to investigate the detailed process of T lymphocyte development under *mcm5* loss-of-function conditions. Notably, the number of *coro1a*: GFP-labeled cells did not differ between siblings and *mcm5*^**−/−**^ embryos at 3.5 dpf (Fig. 2D and Fig. S9B, C, G, H), but it was greatly reduced at 4dpf (Fig. 2D and Fig. S9E, J). This phenotype was also rescued by *mcm5* mRNA injection (Fig. 2D, E). Subsequently, we compared the T cell developmental process between siblings and *mcm5* mutants from 3.2 dpf to 4 dpf (Fig. S9). In siblings the number of T cell continuously increases (Fig. S9A–E), but in *mcm5* mutants the T cells continuously decreased (Fig. S9H–J). In addition, more *coro1a*:GFP-labeled cells appeared to undergo apoptosis in *mcm5*^**−/−**^ embryos than in WT embryos (Fig. S9G–I, white arrow). These findings suggested that T cells in *mcm5* mutants may undergo apoptosis, cell cycle arrest, or both. To evaluate this hypothesis, TUNEL assays, H3p immunostaining and EdU incorporation assays were performed to evaluate T-cell apoptosis and proliferation independently. The results indicated that immature T lymphocytes exhibited delayed proliferation (Fig. 2G and Fig. S10A, B) and an enhanced pro-apoptotic phenotype (Fig. 2F). To further clarify whether *mcm5* is exclusively involved in T lymphocyte maturation rather than early events in T lymphocyte development, a *Tg(Hsp70l:mcm5-P2A-mCherry)* transgenic line was generated (Fig. S11A). Subsequently, *mcm5-P2A-mCherry* expression was temporally induced at 3 dpf via heat shock to specifically rescue T-cell maturation in *mcm5*^**−/−**^ embryos (Fig. S11A, B). Following this treatment, the population of immature T cells was restored in *mcm5*^**−/−**^ embryos (Fig. 2D, E, and Fig. S11C). These results further confirmed that, in *mcm5*^**−/−**^ embryos, immature T cells, rather than early progenitors, undergo apoptosis starting at 3.5dpf and persisting thereafter.

### Tp53 signaling mediates the increase in T-cell apoptosis upon loss of *mcm5* function

**Fig. 3:**
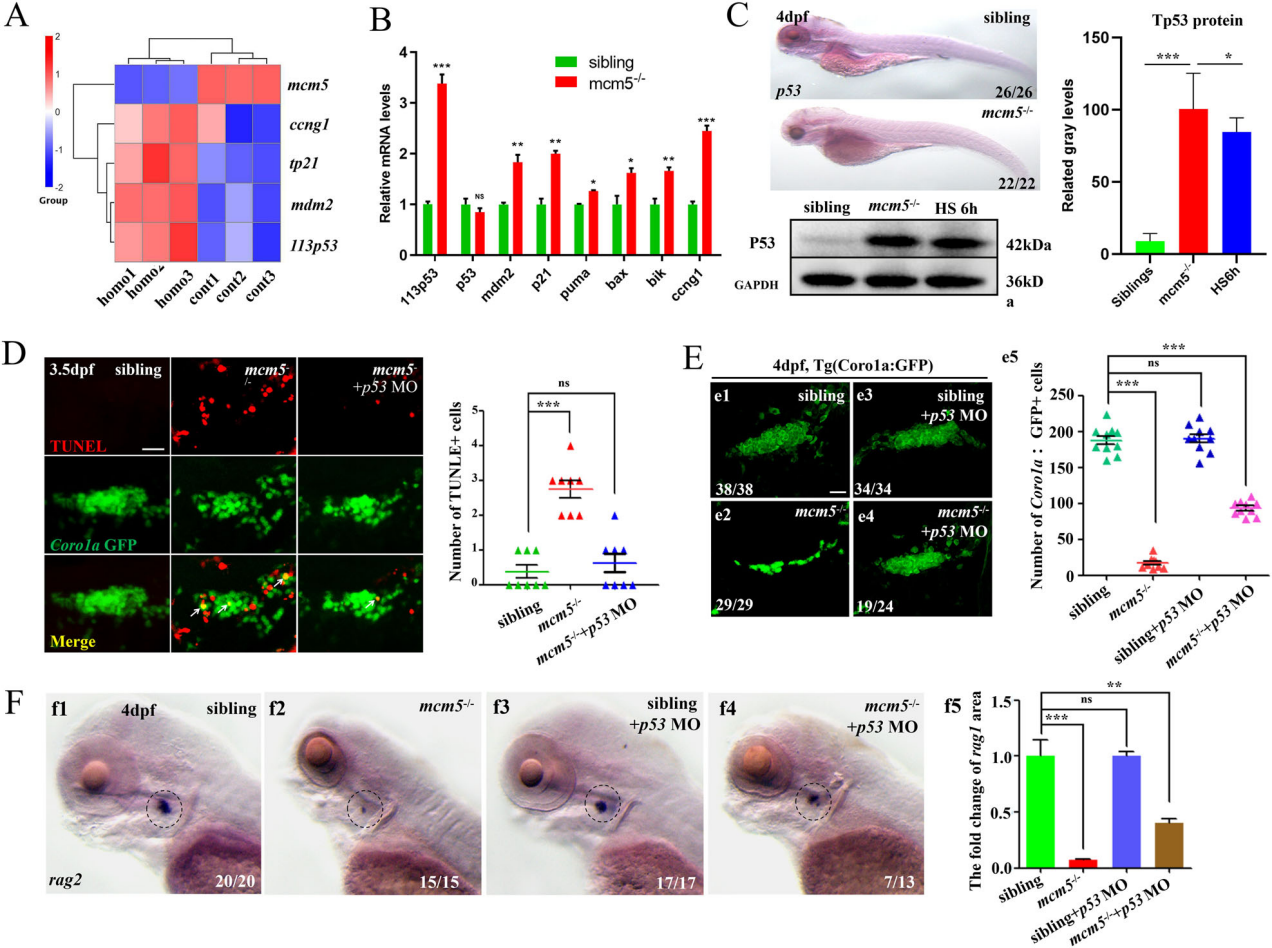
p53-dependentproapoptotic signaling mediates the death of immature T lymphocytes. **A** Comparison of the expression of critical p53 signaling genes and cell cycle genes. **B** Relative expression of *tp53*, the *tp53* downstream genes *113p53*, *mdm2* and *p21*, *puma*, *bax*, *bik* and the cell cycle gene *ccng1*. Besides the expression of *tp53*, all of the genes were upregulated in *mcm5*^*−/−*^ embryos. As shown by RT‒qPCR, the experiments were repeated at least 3 times, and the value was normalized to that of β-actin. **C** The expression of *tp53* was evaluated by WISH and Western blotting for siblings, *mcm5*^*−/−*^ embryos and siblings treated with heat-shock. The right columns show the relative gray level for Western blotting. The mRNA level of *tp53* was not upregulated in *mcm5*^*−/−*^ embryos, but the protein level was greatly increased. **D** TUNEL staining was evaluated for *Coro1a*:GFP-labeled cells at 3.5 dpf in siblings (*n* = 7), *mcm5*^*−/−*^ (*n* = 7) and *mcm5*^*−/−*^ embryos injected with *p53*MO (*n* = 7). **E** On 4 dpf, the number of *Coro1a*: GFP-labeled cells in siblings (e1, *n* = 38), *mcm5*^*−/−*^embryos (e2, *n* = 29), embryos injected with *p53* MO (e3, *n* = 34) and *mcm5*^*−/−*^embryos injected with *p53* MO (e4, *n* = 24) was examined. The number of *Coro1a*: GFP-labeled cells in *mcm5*^*−/−*^embryos was partially restored by injecting *p53*MO (e5). **F** The expression of *rag2* was examined in siblings (f1, *n* = 20), *mcm5*^*−/−*^embryos (f2, *n* = 15), embryos injected with *p53* MO (f3, *n* = 17) and *mcm5*^*−/−*^embryos injected with *p53* MO (f4, *n* = 13). The expression of *rag2* in *mcm5*^*−/−*^embryos was partially restored by injecting *p53*MO (f4-f5). Scale bars, 40 μm. For (**B**, **C**), e5 and f5, the data were presented as means ± SD; The *P*-values (t-test; two-tailed); NS, not significant. “*” *P* < 0.05, “**” *P* < 0.01, “***” *P* < 0.001.

To elucidate the mechanism underlying apoptotic death of immature T lymphocytes in *mcm5*^*−/−*^ embryos, we conducted RNA-seq analysis comparing siblings and *mcm5*^*−/−*^ embryos. In mcm5 mutant embryos, the transcriptions of 378 genes were significantly altered (Fig. S12A). Among these, in addition to genes associated with eye development and function (Fig. S12B), the expression of genes related to the cell cycle, apoptosis and Tp53 signaling was also altered in *mcm5*^**−/−**^ embryos (Fig. S12C, D). Given that several *tp53* isoforms have been identified in zebrafish [23, 46], we further analyzed which *tp53* isoform was upregulated in *mcm5*^**−/−**^ embryos. The results indicated that, rather than full-length *tp53*, the *tp53* downstream gene *Δ113p53* (a N-terminal-truncated p53 isoform whose transcription is initiated from an alternative p53 promoter [47]) was upregulated (Fig. 3A). These findings were consistent with the results of in situ studies, RT-qPCR and Western blotting: although the transcript level of *tp53* was unchanged (Fig. 3B, C and Fig. 3C Gel Supplementary), Tp53 protein expression and the transcription of P53 downstream genes (*mdm2, Δ113p53* and *tp21)* were upregulated (Fig. 3B, C). This result suggested that in *mcm5*^*−/−*^ embryos, *tp53* translation might be enhanced, Tp53 protein degradation reduced, or *Tp53* protein stability increased. Furthermore, we observed that the expression of *tp53* downstream genes *tp21* and *mdm2*, but not *tp53* itself, was obviously upregulated in the thymus region (Fig. S12E), implying the possibility that upregulation of Tp53 signaling mediates immature T-cell apoptosis in *mcm5*^**−/−**^ embryos. Indeed, blocking *tp53* translation via injection of a *tp53*MO [38] inhibited apoptosis in immature lymphocytes (Fig. 3D) and significantly restored T lymphocyte numbers (Fig. 3E, F). These results demonstrated that Tp53 signaling upregulation mediates T-cell apoptosis following the loss of *mcm5* function.

### Immature T cells in *mcm5* mutants exhibit increased sensitivity to DNA damage

**Fig. 4:**
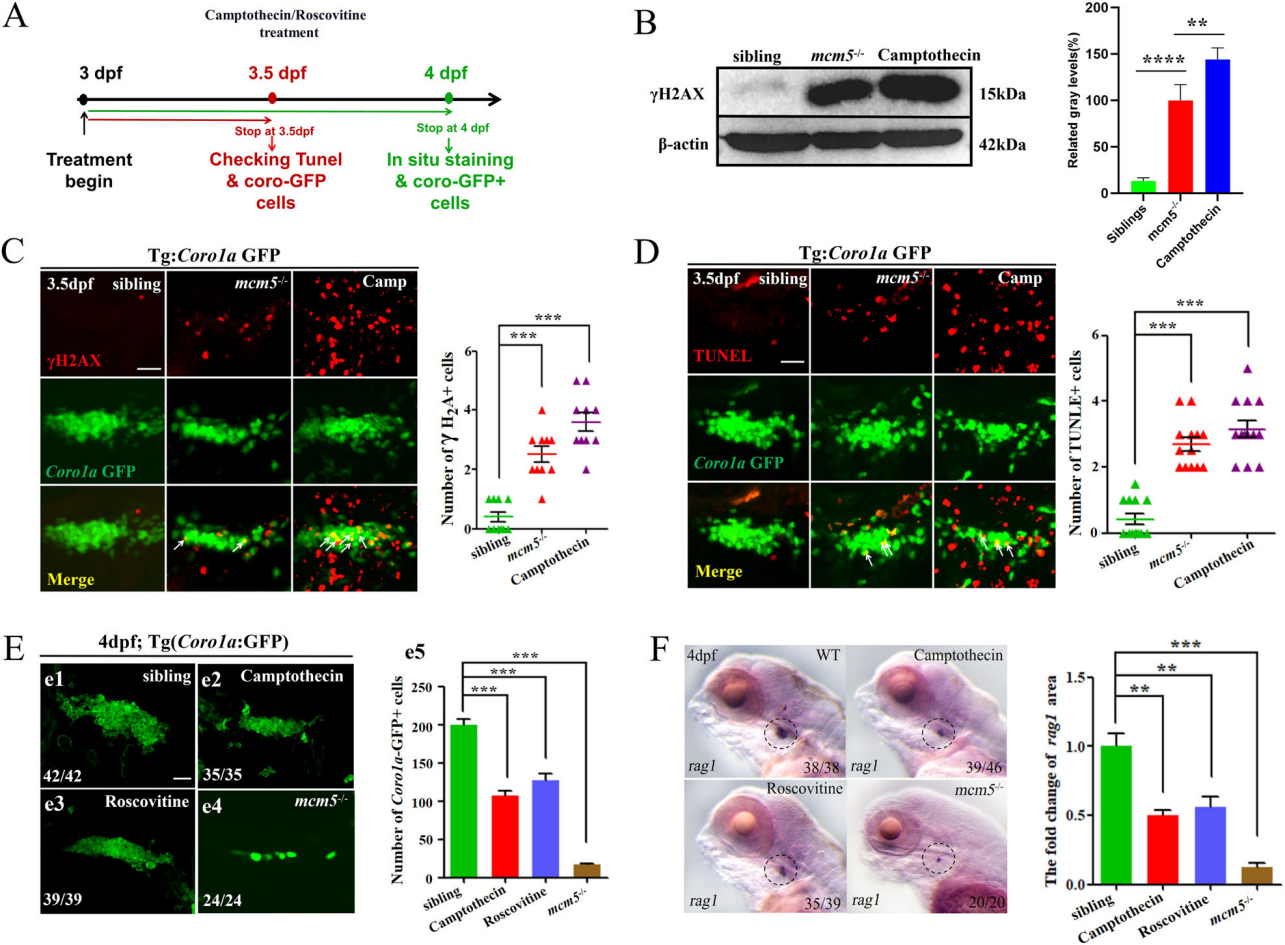
Immature T cells in *mcm5* mutants are more sensitive to DNA damage. **A** Schematic for chemical treatment and subsequent examination. **B** γH2AX protein was evaluated by Western blotting. The right columns show the relative gray level. **C** γ-H2AX is a phosphorylated form of H2AX, which is a DNA damage marker. DNA damage was evaluated by γH2AX immunostaining for siblings (*n* = 10), *mcm5*^*−/−*^ embryos (*n* = 10) and siblings treated with camptothecin (*n* = 10). **D** Proapoptotic *Coro1a*:GFP cells were examined using TUNEL staining for embryos with different treatments at 3.5 dpf. More TUNEL-positive *Coro1a*:GFP cells were observed in embryos treated with camptothecin. **E** On 4 dpf, the expression of *Coro1a*:GFP in the thymus was compared among siblings (e1, *n* = 42), embryos treated with camptothecin (e2, *n* = 35) or roscovitine (e3, *n* = 39), and *mcm5*^*−/−*^ embryos (e4, *n* = 24). The number of *Coro1a*:GFP labeled cells was decreased in embryos treated with camptothecin or roscovitine, but the phenotype was not as strong as that in *mcm5*^*−/−*^ embryos (e5). **F** Expression of *rag1* in embryos with different treatments. Embryos treated with camptothecin (45% of WT controls) or roscovitine (56% of WT controls) displayed decreased expression of *rag1*, but the decrease was not as strong as that in *mcm5*^*−/−*^ embryos (10.7% of controls). For (**B**–**F**), the data were presented as means ± SD; The *P*-values (t-test; two-tailed); NS, not significant. “**” *P* < 0.01, “***” *P* < 0.001.

Given that MCM5 is critical for initiating DNA replication, the loss of MCM5 function could lead to DNA replication defects and subsequent DNA damage. Our data showed a significant increase in γ-H2AX protein levels in *mcm5* mutants (Fig. 4B and Fig. 4B Gel Supplementary). Additionally, γ-H2AX expression was elevated in immature lymphocytes in *mcm5* mutants (Fig. 4C), suggesting that DNA damage caused by DNA replication defects in *mcm5* mutants acts as an apoptosis inducer. Camptothecin is a nuclear DNA topoisomerase I (TOP1) inhibitor [48], and roscovitine is a CDK inhibitor [49]. Treatment with either of these chemicals induces DNA damage during the cell cycle [23, 49]. To test whether DNA damage acts as an apoptosis inducer, zebrafish embryos were treated separately with camptothecin and roscovitine. We then examined whether DNA damage caused by these two chemicals induced apoptosis in T lymphocytes (Fig. 4A). Indeed, both chemical treatments resulted in a reduced number of T lymphocytes at 4 dpf (Fig. 4E, F), along with more severe DNA damage and an increased number of TUNEL-positive cells compared to *mcm5*^**−/−**^ embryos (Fig. 4B–D). In addition, we found the *p53* downstream genes *p21* and *mdm2* were upregulated (Fig. S13A), consistent with the findings in *mcm5* mutants. These results suggest that DNA damage induces Tp53 signaling upregulation, which in turn mediates apoptosis in immature T cells. Aphidicolin, a reported DNA replication inhibitor, induces DNA replication defects and subsequent DNA damage upon treatment [50]. To directly evaluate whether DNA damage induced by DNA replication defects acts as an apoptosis inducer and leads to a decreased T-cell number in *mcm5* mutants, aphidicolin was used to treat embryos, and the T-cell developmental process was analyzed. The data showed that aphidicolin treatment also reduced the number of T cells at 4dpf (Fig. S13B). Given that T cell maturation is more sensitive to DNA damage in *mcm5* mutants (Fig. 1 and Fig. 4E), chemical treatments should have minimal effects on early T cell development and HSC development. To evaluate this hypothesis, we examined whether HSCs are significantly affected by chemical treatments administered to embryos before 2.5 dpf. the data showed that, following early-stage chemical treatment, the number of c-myb:GFP-labeled HSCs was not significantly reduced (Fig. S13C). This result further supports the notion that T cell maturation is more sensitive to DNA damage.

Interestingly, although the DNA damage (Fig. 4C) and pro-apoptotic signaling (Fig. 4D) in camptothecin-treated embryos were more severe than those in *mcm5*^**−/−**^ embryos, the number of immature T cells at 4dpf was significantly higher than in *mcm5*^**−/−**^ embryos (Fig.4E, F). These results showed that immature T cells in *mcm5* mutants exhibit enhanced sensitivity to DNA damage. Therefore, an immediate question was why the severe DNA damage induced by the chemical treatments led to a small number of immature T cells undergoing terminal cell death.

### Bcl2a silencing in *mcm5* mutants accelerates apoptosis in immature T cells

**Fig. 5:**
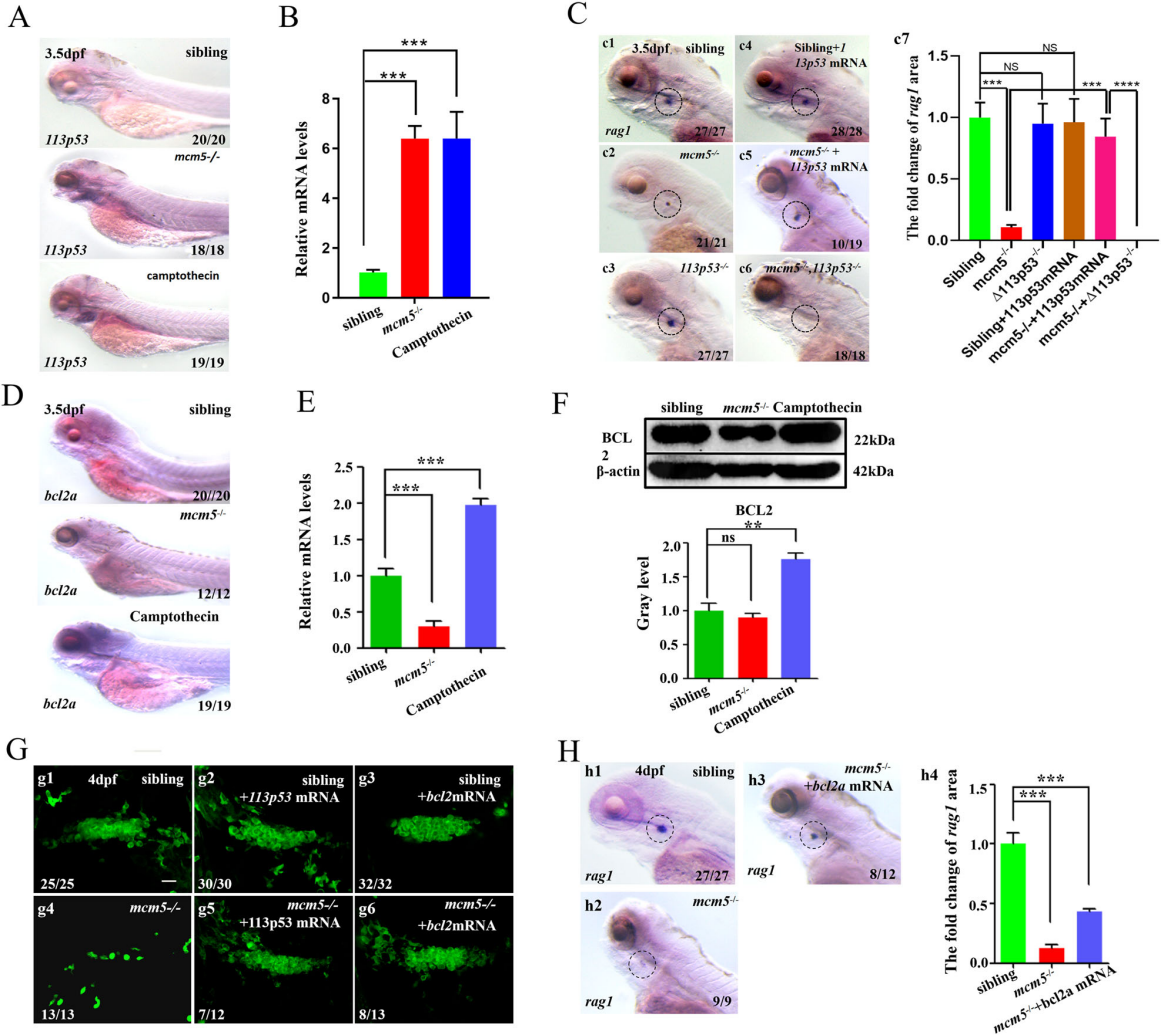
Silencing of *bcl2a* in *mcm5* mutants accelerates the death of immature T lymphocytes. **A** Upregulation of *Δ113p53* expression in *mcm5*^*-/ -*^embryos and embryos treated with camptothecin was confirmed using in situ experiments. **B** RT‒qPCR examination showed that the expression of *Δ113p53* was upregulated in thymus region of *mcm5*^*−/−*^ embryos and embryos treated with camptothecin. **C** The expression of *rag1* in controls (c1, *n* = 27), *Δ113p53*^*−/−*^ embryos (c3, *n* = 27) and embryos injected with *Δ113p53 mRNA* (c4, *n* = 28) was normal (c7). Downregulation of *rag1* expression in *mcm5*^*−/−*^embryos (c2, *n* = 21) was partially reversed by injection of *Δ113p53 mRNA* (c5, *n* = 19; c7) and was exacerbated in *mcm5*^*−/−*^*;Δ113p53*^*−/−*^double mutants (c6, *n* = 18; c7). **D**, **E** The expression of *bcl2a* in different embryos was assessed using WISH (**D**) and RT‒qPCR (**E**). **F** At the protein level, Bcl2 was upregulated in embryos treated with camptothecin but not in *mcm5*^*−/−*^ embryos. **G** In the transgenic line *Tg(coro1a:GFP)*, compared to that in siblings (g1, *n* = 25), *mcm5*^*−/−*^ embryos (g4, *n* = 13) and embryos injected with *Δ113p53* mRNA (g2, *n* = 30), the decrease in *coro1a:GFP* cells in *mcm5*^*−/−*^ embryos was partially rescued by injection of *Δ113p53* mRNA (g5, *n* = 12). Overexpression of *bcl2a* mRNA did not affected the number of *Coro1a*:GFP labeled cells (g3, *n* = 32), but also partially reversed the T-cell developmental defect in *mcm5*^*−/−*^embryos (g6, *n* = 13). **H** Compared to that in siblings (h1, *n* = 27), *mcm5*^*−/−*^ embryos (h2, *n* = 9), the expression of *rag1* was partially rescued by injection of *bcl2* mRNA in *mcm5*^*−/−*^ embryos (h3, *n* = 12; h4). Scale bars, 40 μm. For (**B**, c7, **E** and f4), the data were presented as means ± SD; The *P*-values (t-test; two-tailed); *NS* not significant. “**” *P* < 0.01, “***”*P* < 0.001.

It is well established that DNA replication stress and other developmental stress simultaneously stimulate pro-apoptotic and anti-apoptotic pathways to regulate cell survival or death [23, 51, 52]. To investigate why severe DNA damage in chemically treated embryos resulted in fewer immature T cells undergoing cell death, we examined whether the levels of anti-apoptotic signaling mediators Bcl2 and *Δ113p53* differed in the thymic region between chemically treated embryos and *mcm5* mutants. The data revealed that, in both groups of embryos, *Δ113p53* expression was upregulated in endodermal organs and the head region, including the thymic region (Fig. 5A, B). In *Δ113p53* mutant embryos, an 11 bp deletion in the TP53 response element represses *Δ113p53* transcription upon P53 signaling upregulation ([28] and Fig. S14A–C). To evaluate whether upregulation of *Δ113p53* protects T cell from apoptosis in *mcm5*^*−/−*^ embryos, we examined *rag1* expression in wild type embryos, *Δ113p53*^*−/−*^ embryos and *Δ113p53*^*−/−*^*:mcm5*^*−/−*^ double mutant embryos. The data showed that although the expression of *rag1* was not affected in *Δ113p53* single mutants (Fig. 5Cc3, c7), *Δ113p53* loss of function further decreased *rag1* expression in *mcm5*^*−/−*^ embryos (Fig. 5Cc6, c7). In addition, injection of exogenous *Δ113p53* mRNA (further enhance the anti-apoptosis role) partially rescued the T cell defect in *mcm5*^*−/−*^ embryos (Fig. 5Cc5, c7, G). Although *Δ113p53* expression was upregulated in *mcm5* mutants, it was also increased in embryos treated with camptothecin (Fig. 5A, B); thus, upregulation of *Δ113p53* was not the underlying reason that the embryos treated with the chemicals contained more immature T cells. Next, we examined the expression of *bcl2a* in all these kinds of embryos. The data showed that the transcription of *bcl2a* was downregulated in *mcm5* mutants (Fig. 5D–F and Fig. 5F Gel Supplementary), whereas in chemically treated embryos, the expression of *bcl2a* was upregulated at 3.5 dpf and 2.5 dpf (Fig. 5D–F; Fig. S13D). These results implied that the absence of upregulation of Bcl2 in *mcm5* mutants resulted in an inability to protect T cells from apoptosis, whereas in embryos treated with chemicals, upregulated Bcl2 signaling protected T cells from apoptosis. Indeed, additional data revealed that T-cell numbers were partially restored at 4 dpf in *mcm5*^*−/−*^ embryos following injection with *bcl2a* mRNA (Fig. 5G, H). Conversely, in chemically treated embryos, transient knockout of *bcl2a* via sgRNR-Cas9 complex injection at the one-cell stage (Fig. S15A, B) significantly reduced T lymphocyte numbers compared to controls (Fig. S15C, c2-c6). These findings suggest that Bcl2 signaling cannot be efficiently enhanced in *mcm5* mutants, thereby accelerating the apoptotic death of immature T cells.

### Downregulation of stat1 signaling mediates silencing of the Bcl2 cascade in *mcm5* mutants

**Fig. 6:**
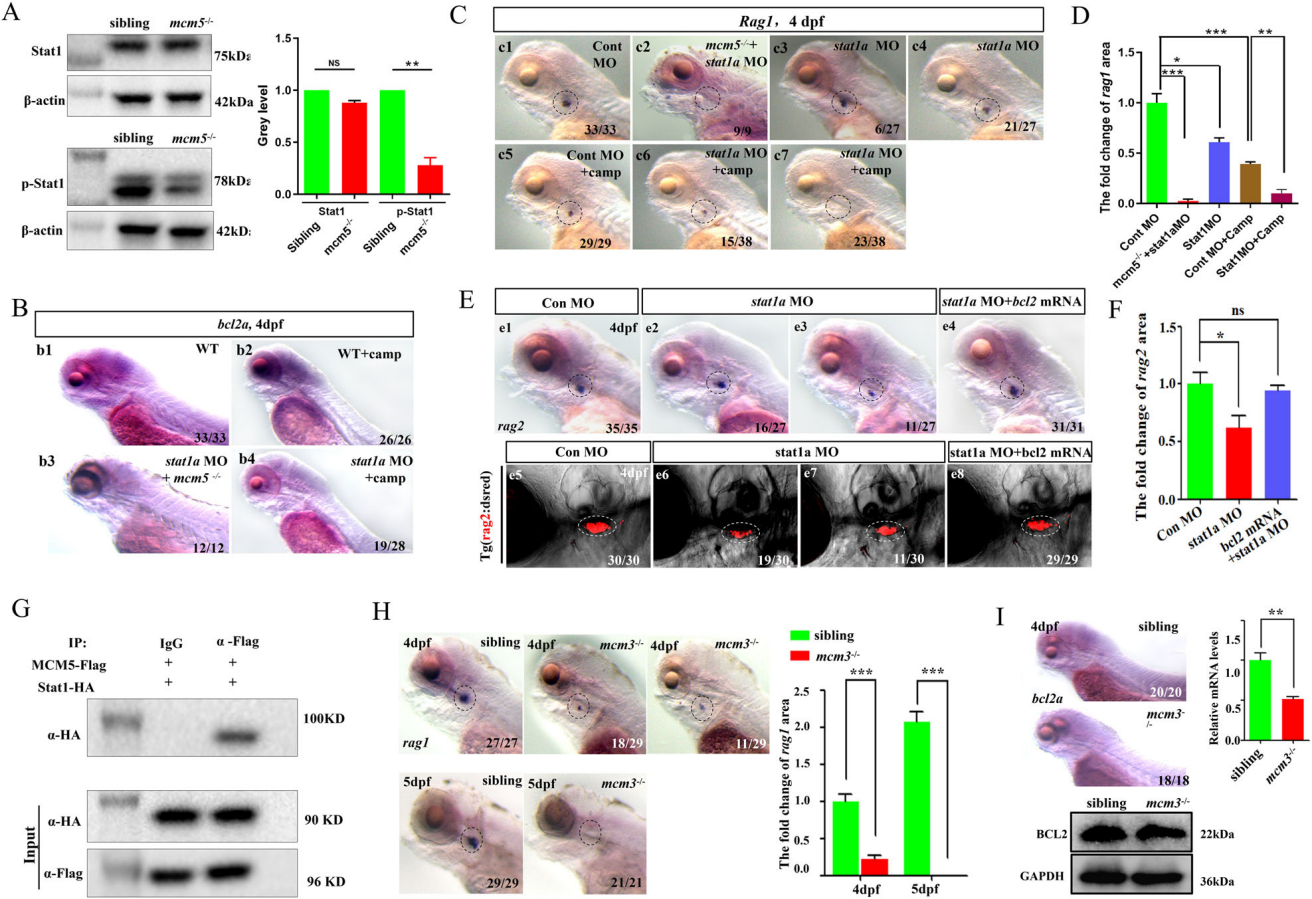
The MCM5/stat1 complex is required for Stat1 phosphorylation and downstream gene *bcl2a* transcription. **A** The proteins Stat1 and p-Stat1 were evaluated in siblings and *mcm5*^*−/−*^ embryos at 3.5 dpf. p-Stat1 but not Stat1 was downregulated in *mcm5*^*−/−*^ embryos. **B** The expression of *bcl2a* in embryos with different treatments. The data showed that *stat1a* MO injection further downregulated *bcl2a* expression in *mcm5* mutants, and *stat1a* MO injection also decreased *bcl2a* expression in embryos treated with camptothecin. **C** In situ staining for the *rag1* probe in embryos. *stat1a* MO injection further decreased the expression of *rag1* in *mcm5* mutants (c2, *n* = 9). Compared to controls (c1, *n* = 33), embryos injected with *stat1a* MO displayed decreased expression of *rag1* (c3, c4, *n* = 27). Compared to controls (c5, *n* = 29), *stat1a* MO injection led to a more severe decrease in *rag1* expression when camptothecin treatment was applied (c6, c7, *n* = 38). **D** Quantification of the area of *rag1* expression in different kinds of embryos. **E** The expression of *rag2* in the thymus. Compared to controls (e1, *n* = 35), the expression of *rag2* was decreased in embryos injected with *stat1a* MO (e2, e3, *n* = 27). Injection of *bcl2a* mRNA rescued the expression of *rag2* in WT embryos injected with *Stat1a* MO (e4, *n* = 31). The DsRed-labeled T cells were examined in different kind of embryos (e5-e8). Compared to controls (e5, *n* = 30), the expression of DsRed in thymus was decreased in embryos injected with *stat1a* MO (e6, e7, *n* = 30). The expression of DsRed in *stat1a* morphants was also rescued by injecting *bcl2a* mRNA (e8, *n* = 29). **F** Quantification of the expression of *rag2* in e1 to e4. **G** Examination of the interaction of zebrafish MCM5 and zebrafish Stat1a using a Co-IP experiment. In first lane, IgG was used as negative control to carry out IP experiment, anti-Flag was used to examine the interation between Stat1 and MCM5, anti-HA was used to perform west blotting staining. The staining showed that MCM5 binds to Stat1a. **H** The expression of *rag1* in the thymus was greatly downregulated in *mcm3*^*−/−*^ embryos but not as strongly as that in *mcm5*^*−/−*^ embryos. **I** The expression of *bcl2a* in siblings and *mcm3*^*−/−*^ embryos was examined using WISH, WB and RT‒qPCR. For (**A**, **D**, **F**, **H**, **I**), the data were presented as means ± SD; The *P*-values (t-test; two-tailed); *NS* not significant. “*”*P* < 0.05, “**”*P* < 0.01, “***”*P* < 0.001.

Previous studies have reported that Stat1 activation enhances bcl2 expression and promotes leukemia [13]. Additionally, neutrophil counts are increased in zebrafish *stat1a* morphants [39]. Here, we found that in *mcm5* mutants, neutrophil counts and the expression of *mpeg1.1* were increased(Fig. S16A, B), the expression of Stat1 downstream genes *tap1*, *gbp1*, *irf1b*, and *irf1a* was downregulated in the RNA-seq result and was confirm by RT-qPCR data (Fig. S16C), suggesting that Stat1 signaling is suppressed in *mcm5* mutants. To test this hypothesis, the Stat1 and p-Stat1 protein levels were measured. The level of p-Stat1 was decreased, but that of Stat1 was not decreased in *mcm5* mutants (Fig. 6A and Fig. 6A Gel Supplementary 1-2). These findings demonstrate that downregulation of Stat1 signaling in *mcm5* mutants may contribute to reduced *bcl2* transcription and the subsequent T-cell defects. To evaluate this hypothesis, we injected *stat1a* MO to block *stat1a* translation in *mcm5* mutants and subsequently analyzed the expression of *bcl2a* and *rag1*. Indeed, injection of *stat1a* MO further decreased the expression of *bcl2a* and *rag1* in *mcm5*^*−/−*^ embryos at 4 dpf (Fig. 6Bb3, Cc2, D). Given this finding, *stat1a* loss of function was expected to block *bcl2a* transcription in embryos treated with camptothecin. Indeed, *stat1a* MO injection repressed the upregulation of *bcl2a* in embryos treated with camptothecin (Fig. 6Bb2, b4), suggesting that Stat1 mediates Bcl2 signaling to protect immature T cells from apoptosis under DNA damage stress. To further evaluate this hypothesis, we injected *stat1a* MO into embryos, treated with camptothecin at 3 dpf and examined T lymphocyte development. The data showed that *stat1a* inhibition resulted in a substantial decrease in immature T cells compared to controls at 4dpf (Fig. 6Cc5-c7, D). Additionally, *bcl2a* mRNA injection partially restored T lymphocyte development in embryos injected with *stat1a* MO and treated with camptothecin (Fig. 6Ee2-e4, e6-e8, F). These results demonstrate that enhanced Stat1-Bcl2 signaling protects immature T cells from terminal apoptosis in embryos treated with camptothecin, whereas the lack of Stat1-Bcl2 axis upregulation accelerates immature T-cell apoptosis in *mcm5* mutants.

To elucidate how Mcm5 facilitates Stat1a signaling to promote *bcl2a* transcription in zebrafish, the interaction between Stat1a and Mcm5 was analyzed using Co-IP. The experiments demonstrated that zebrafish Mcm5 and Stat1a bind to each other (Fig. 6G, Fig. S16D, Fig. 6G Gel Supplementary, and Fig. S16D Gel Supplementary), suggesting that Mcm5 directly interacts with Stat1a and facilitates its phosphorylation to regulate *bcl2a* transcription. A previous study reported that MCM3 acts as a partner for MCM5 to activate target gene expression in vitro [53, 54]. This fact implies that Mcm3 is required for the activation of the downstream gene *bcl2a* by the Mcm5/Stat1 complex and that the T-cell developmental defect in *mcm3* mutants should be similar to that in *mcm5* mutants. Indeed, in *mcm3* mutants (Fig. S17A–E), *rag1* expression and the number of Coro-GFP labeled cells were decreased at 4 dpf (Fig. 6H and Fig. S18A). Additionally, more immature T cells exhibited increased pro-apoptotic signaling and DNA damage compared to controls (Fig. S18B–D and Fig. S18C Gel Supplementary). Furthermore, *bcl2a* transcription was downregulated, whereas *Δ113p53* transcription was upregulated (Fig. 6I, Fig. S18E and Fig. 6I Gel Supplementary). Moreover, the number of immature T cells was reduced compared to embryos treated with camptothecin, and *bcl2a* mRNA injection partially rescued T lymphocyte development defects (Fig. S18A). These findings further support the notion that the Mcm5/Stat1 complex is essential for upregulating *bcl2a* expression to protect immature T cells from rapid death under DNA replication stress.

### The role of Mcm5 in T-cell maturation is conserved during mouse T-cell development

**Fig. 7:**
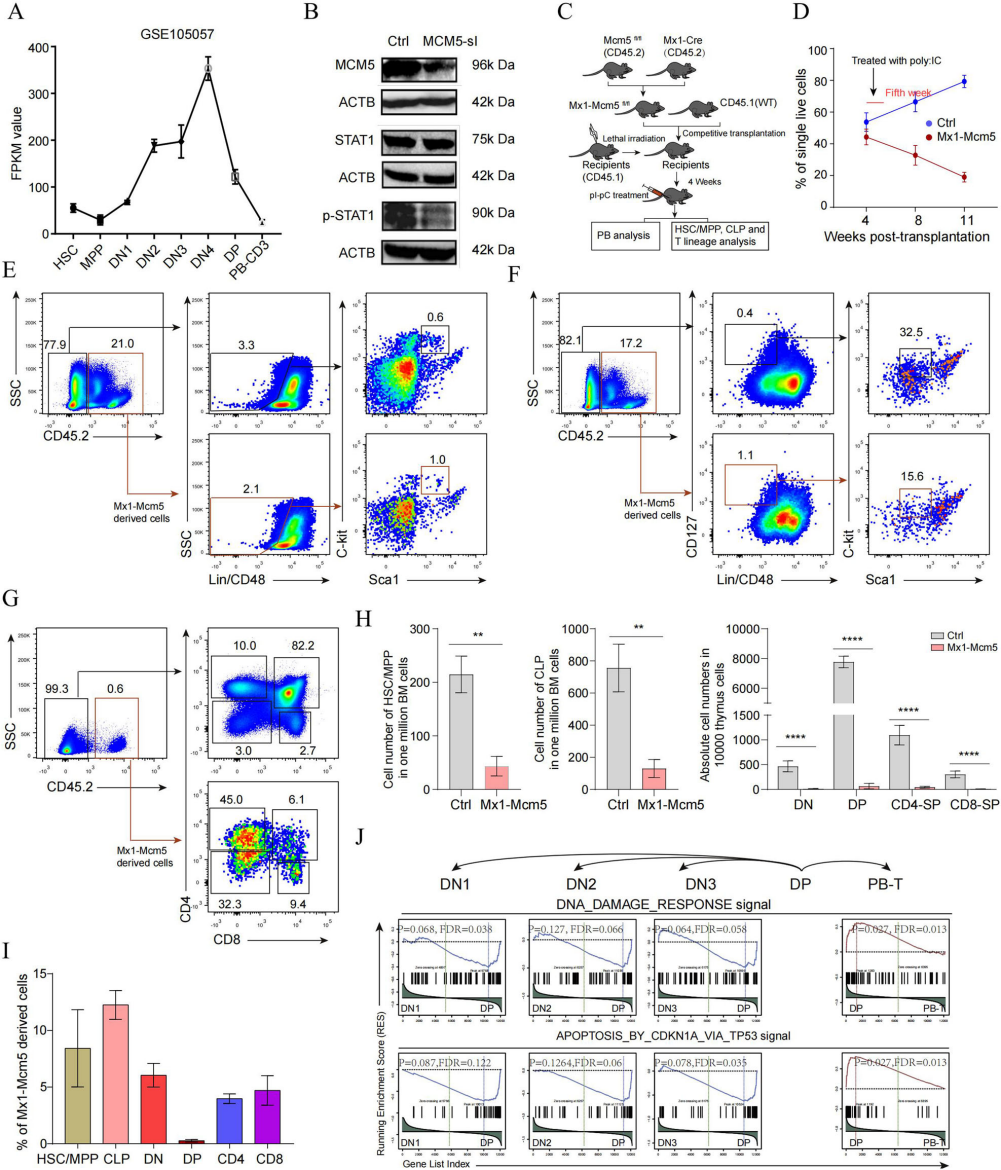
CD4^+^CD8^+^ DP cells are most sensitive to Mcm5 knockout. **A** Expression levels of mouse Mcm5 in different stages of T differentiation. The data are derived from GSE105057. **B** The expression level of Stat1 and p-Stat1 in 293 T cells before and after Mcm5 was knocked down. When Mcm5 was knocked down, the level of p-Stat1 but not Stat1 was decreased. **C** Experimental flowchart of the analysis of the impact of Mcm5 knockout on T lineage differentiation. **D** Statistical analysis of Mcm5^−/−^ and WT-derived contributions in the PB of recipients. The ratio of Mcm5^−/−^ derived PB decreased at 8 and 11 weeks post-transplantation. **E**–**G** Flow cytometry analysis of hematopoietic stem cells/multipotential progenitors (HSCs/MPPs) (**E**), common lymphocyte progenitors (CLPs) (**F**), CD4^-^CD8^-^ double-negative (DN) T progenitors, CD4^+^CD8^+^ double-positive (DP) T progenitors, CD4^+^CD8^-^ and CD4^-^CD8^+^ single-positive T cells (**G**) in wild type control cells (CD45.1) and Mcm5^−/−^ derived cells (CD45.2). **H** Cell number of HSCs/MPPs, CLPS and different kinds of T cells in one million analyzed cells. Compared to wild type control cells (CD45.1), all kinds of Mcm5^−/−^ derived cells (CD45.2) are greatly decreased. **I** Compared to wild type controls, statistical analysis of the ratio of Mcm5^−/−^derived HSCs/MPPs, CLPs, DN T progenitors, DP T progenitors, and CD4^+^CD8^-^ and CD4^-^CD8^+^ single-positive T cells in the bone marrow and thymus of recipients. **J** Gene set enrichment analysis (GSEA) of the DNA damage response and apoptosis-related signals in DN1-, DN2-, DN3-, DP- and PB-derived mature CD3^+^ T cells. The transcriptome data were derived from GSE105057. The data are representative of two independent experiments (**E**–**G**) or are pooled from two independent experiments (**H**, **I**; mean ± SD of *n* = 4 biological replicates). The data in (**A**, **D**, **H**, **I**) are presented as the means ± SDs. NS not significant. “*” *P* < 0.05, “**” *P* < 0.01, “***” *P* < 0.001, “****” *P* < 0.0001.

Interestingly, a previous study has shown that the expression of mouse Mcm5 is increased during T-cell maturation (Fig. 7A, [55] GSE105057). Our data also revealed that the p-Stat1 level, but not the Stat1 level, was significantly reduced in mammalian 293 T cells following Mcm5 knockdown (Fig. 7B and Fig. 7B Gel Supplementary 1–3). These results implied that the role of Mcm5 in T-cell maturation might be conserved in mice. To evaluate this hypothesis, a Mcm5 conditional knockout mouse (C57BL/6J-Mcm5^em1cyagen^) was generated using the LoxP system (Fig. S19A). This mouse line was then crossed with Mx1-Cre mice to generate Mcm5f/f; Mx1-Cre mouse, which were used to create hematopoiesis-specific Mcm5-knockout (Mcm5^−/−^) mice when induced by pI-pC (Fig. 7C). Bone marrow competitive transplantation was performed to analyze the impact of Mcm5 knockout on hematopoietic development (Fig. 7C). Donor-derived leukocytes (CD45.1+ and CD45.2+ cells) were analyzed at 3 and 6 weeks after pI-pC treatment (pI-pC was performed at 5 weeks after transplantation) (Fig. 7D). The results showed that, at 1.5 weeks after treatment with pI-pC, the level of Mcm5 protein was decreased (Fig. S19B), at 3 and 6 weeks after treatment with pI-pC, the number of Mcm5^−/−^ derived leukocytes continuously decreased in peripheral blood (Fig. 7D). Next, we analyzed the effect of Mcm5 knockout on hematopoietic stem cells (HSCs), common lymphocyte progenitors (CLPs) in the bone marrow and T-lineage cells in the thymus at 4 to 5 weeks after treatment with pI-pC. We found that, compared to transplanted control cells (CD45.1+ cells), the number of HSCs (Fig. 7E, H), CLPs (Fig. 7F, H), and all types of T-lineage cells (Fig. 7G, H) were significantly reduced in Mcm5 knockout cells (CD45.2+ cells), suggesting that Mcm5 is essential for the maintenance of all analyzed cell types. To further evaluate which kind of cell is more sensitive to Mcm5 knockout, we compared the ratio of each kind of cell in Mcm5 knockout to total cells in each group. Notably, the ratio of CD4^+^CD8^+^ double-positive (DP) cells was most low, almost disappeared, but not the HSCs and CLPs or mature T cells (CD4^+^ single-positive T cells and CD8^+^ single-positive T cells) (Fig. 7I). This suggests that DN to DP-staged T cells are the most sensitive to Mcm5 knockout. This result showed that the function of Mcm5 in T-cell maturation is conserved in mice during T-cell development. Furthermore, in wild type mice, GSEA of lymphocyte progenitors and PB-derived T cells compared to DP cells revealed that the DNA damage response and apoptosis-related signals (Tp53 signal) were more active in DP cells (Fig.7J, [55] GSE105057). This implies that the mechanism underlying the sensitivity of immature T cells to cell death upon Mcm5 loss of function is conserved between zebrafish and mice. After analyzing the role of Mcm5 in T cell development, we also observed the B cell development using bone marrow competitive transplantation when Mcm5 was knocked out. The data also showed that B cell maturation is more sensitive to Mcm5 knockout (Fig. S19C–F). This data indirectly suggest that immature T cells are more sensitive to Mcm5 knockout during hematopoietic process.

## Discussion

During hematopoiesis, T lymphocytes exhibit heightened sensitivity to developmental stresses, including DNA replication stress [17, 18]. One possible mechanism is related to T-cell specification and maturation, since in this stage, V(D)J rearrangement of T-cell receptor (TCR) genes occurs in developing T cells, and additional specific genes are transcribed [56, 57]. In *top3a*^*−/−*^ zebrafish embryos, developing lymphocytes exhibit specific and heightened impairment; however, suppressing *rag1* function does not alleviate the severe phenotype [17]. These findings suggest the presence of a complex mechanism underlying T lymphocyte development, which remains poorly understood.

Our data showed that in zebrafish *mcm5*^*−/−*^ embryos, immature T lymphocytes were more sensitive to DNA damage during T-cell development, whereas primary hematopoiesis and erythrogenesis were not significantly affected. The possible explanation is that, during normal T-cell maturation, V(D)J rearrangement of T-cell receptor (TCR) genes leads to more frequent DNA damage in T cells compared to other types of hematopoietic cells under stress. In this situation, Mcm5 loss of function further exacerbates DNA damage in T cells, making immature T cells more prone to apoptosis. Similar results have also been observed in other studies in which only T-cell developmental defects were observed [17, 18]. The data in mice further suggested that the critical role of Mcm5 in T-cell maturation is conserved in mammals (Fig. 7). In addition, our data showed that the role of *mcm5* is likely mediated mainly in a T lymphocyte-autonomous manner since *Mcm5* was highly expressed in immature mouse T cells (Fig. 7A) and double positive (DP) T cells almost disappeared when Mcm5 was specifically knocked out in hematopoietic cells (Fig. 7I). Previous studies have shown that in zebrafish, some HSCs migrate into the thymus to develop into T lymphocytes and mature beginning at 7dpf [58]. Therefore, the thymus, as a T-cell developmental niche, is critical for T-cell maturation [40, 58]. In addition, *foxn1* has been reported to lie upstream of *mcm2* to regulate T-cell development [40], the area of *ccl25a* and *foxn1* expression was decreased in *mcm5*^**−/−**^ embryos (more condensed appearance, Fig. S6B–F), so the thymic developmental defect in *mcm5* mutants should also contribute, at least partially, to this stage-specific role of *mcm5* in T-cell maturation. Besides the reduced thymus size, we also found multiple tissues display reduced size, including eyes, head, and body length [59]. Although the mechanism underlying the reduced size of multiple tissues remains unknown, we speculate that it might be partially similar to that in the thymus. Because Mcm5 mutation leads to DNA replication defect and DNA damage in proliferating cells, which resulting in DNA damage response, including DNA repair process and delaying cell proliferation. When the DNA damage could not be repaired and the cells could not pass through cell cycle, cell apoptosis would occur.

Mechanistically, our data showed that under DNA replication stress, loss of *mcm5* function decreased the level of Stat1 phosphorylation and the transcription of *bcl2a* (Fig. S20A). In contrast, under transient DNA replication stress, normal Mcm5 increased the level of *bcl2a* to protect T cells from apoptosis (Fig. S20B). This model partially explains why immature T lymphocytes are sensitive to DNA damage during T-cell maturation in *mcm5* mutants. More importantly, the MCM2-5 proteins work as a whole complex to regulate DNA replication in proliferating cells, our current data (Fig. 6H and Fig. S18A) and previous literature [40] also showed the number of immature T cells were greatly decreased in these kinds of mutants, implying the possibility that other MCM proteins play similar role via similar mechanism during T cell development. Far more work is needed to fully elucidate the role of MCM proteins in T cell development.

A noteworthy observation is that, although immature T cells in mice are the most sensitive to Mcm5 knockout, nearly all DP T cells disappeared when Mcm5 was knocked out (Fig. 7H, I), whereas a subset of CD4+ or CD8+ single-positive T cells remained in the thymus (Fig. 7H, I). It remains unclear whether the remaining CD4+ or CD8+ cells are those that have abnormally passed through the DN-to-DP stage bottleneck and possess clonally rearranged Tcrb genes. However, we speculate that this possibility exists, similar to the situation observed in Mcm4 (D573H) allele mouse [6]. Further evidence is required in future mouse studies to validate this possibility. Alternatively, since CD4+ or CD8+ cells have a long lifespan, it is possible that some of the remaining CD4+ or CD8+ cells were already single-positive before pI-pC treatment.

Although the mechanism underlying the sensitivity of developing T lymphocytes to DNA damage remains unclear, the biological significance of this phenomenon is evident, as the accumulation of DNA damage contributes to carcinogenesis with aging [6, 56]. Therefore, to prevent leukemia, immature T lymphocytes with DNA damage resulting from gene mutations tend to undergo cell death efficiently. However, paradoxically, to maintain life, organisms and cells, including immature T lymphocytes, must develop mechanisms for protection from rapid death under transient environmental replication stress. Indeed, our data demonstrated that the anti-apoptotic factor *Δ113p53* was highly expressed upon endogenous DNA replication stress and transient exogenous DNA replication stress (Fig. 5A), but silencing the stat1-bcl2 cascade accelerated rapid T-cell death only upon endogenous DNA damage in *mcm5* mutants. Notably, in our study, although *bcl2a* expression was downregulated in *mcm5* mutants, *bcl2a* mRNA injection only partially reversed the T-cell developmental defect in *mcm5* mutants. One possible explanation is the presence of other anti-apoptotic genes. Another possible explanation is that in *mcm5* mutants, although *bcl2a* mRNA expression was restored, the protein level of Tp53 (an apoptosis inducer) remained upregulated, leading T cells to undergo apoptosis. Therefore, the rescue effect was incomplete.

Mouse Pole3 has been identified as playing an essential role in T-cell differentiation. This role is linked to a nonreplicative function of the ubiquitous POLE complex [60], suggesting that DNA replication factors have additional roles beyond regulating DNA replication [61]. Furthermore, previous research demonstrated that Mcm2-7 proteins are loaded onto DNA at levels 20-fold higher than the number of replication origins [62], enabling dormant origins to initiate DNA replication under replication stress [63]. Even though the cells continue to proliferate for several days when Mcm2-7 complex is greatly downregulated, the cells are hypersensitive to DNA replication stress [63]. These findings suggest the multifunctionality of the MCM complex in maintaining genomic integrity under normal DNA replication conditions and replication stress. They also imply that cells cannot proliferate with normal genomic integrity or survive long-term in the complete absence of the MCM complex. More recently, a detailed analysis showed that most dormant DNA replication origins are within highly transcribed genes [61], implying that most of the components of ORC, including the members of the MCM family, are involved in gene expression regulation. Indeed, as to Mcm5, our previous work identified endoderm cells migration and facial motor neuron development are regulated by *mcm5* partially via *cxcr4a* and FGF signaling, which are independent of the role of *mcm5* in DNA replication [29, 59]. Here, our research further showed that Mcm5 plays a critical antiapoptotic role through the Stat1-bcl2a axis (Fig. S20B), providing a mechanism by which immature lymphocytes with DNA damage caused by gene mutation are efficiently cleared via apoptotic cell death: through orchestration of upregulation of the Tp53 cascade and blockade of Bcl2 upregulation. While paradoxically, Vetro et al reported a patient with biallelic variants in MCM5 gene, in this patient only Meier-Gorlin syndrome was reported, the population of T- and NK-cells was normal [64]. Why is there no distinct T cell phenotype in this patient? Although we have no data to completely explain this difference, one possible reason is that, the mutations in our zebrafish model and the patient are different. In this reported patient, one of MCM5 variants is a missense substitution mutation within a conserved domain critical for the helicase activity [64], possibly some function of MCM5 was still maintained in this MCM5 variant. In contrast, in our zebrafish *mcm5*^*−/−*^ embryos, biallelic mutation in *mcm5* caused a frameshift and a premature stop codon ([29] and Fig. S3A, B). The differences in disorders between this patient and our zebrafish model, as well as our explanation, align with previous zebrafish data [29, 37]. In these reports, although maternal Mcm5 protein/mRNA supported normal development of other organs and tissues during early stages, eye, head, and neural development were severely disrupted upon Mcm5 downregulation.

## Supplementary information

Supplementary information

Western Blot Gel image (merged in one PDF file).

## Data Availability

All data are available in the main text or the supplementary materials. The RNA-seq data was submitted in GEO database (GSE236184). Additional data or information can be required by contacting with the corresponding author.
