# Correction: Mcm5 mutation leads to silencing of Stat1-bcl2 which accelerating apoptosis of immature T lymphocytes with DNA damage

**DOI:** 10.1038/s41419-025-07674-1

**Published:** 2025-06-03

**Authors:** Min Liu, Yuanyuan Li, Zhilin Deng, Ke Zhang, Shuying Huang, Jiamin Xia, Yi Feng, Yundan Liang, Chengfu Sun, Xindong Liu, Shurong Li, Bingyin Su, Yong dong, Sizhou Huang

**Affiliations:** 1 Development and Regeneration Key Laboratory of Sichuan Province, Department of Anatomy and Histology and Embryology, School of Basic Medical Sciences, Chengdu Medical College, Chengdu, 610500 China; 2 Department of Neurology, the Second Affiliated Hospital of Chengdu Medical College, Nuclear Industry 416 Hospital, Chengdu, 610000 China; 3 Centre for Inflammation Research, Queen’s Medical Research Institute, Institute for Regeneration and Repair, The University of Edinburgh, Edinburgh, UK; 4 Department of Pathology and Pathophysiology, Chengdu Medical College, Chengdu, 610500 China; 5 Department of Immunology, School of Basic Medical Sciences, Chengdu Medical College, Chengdu, 610500 China; 6 Present Address: Department of Cardiology, The First Affiliated Hospital, Chengdu Medical College, Chengdu, 610500 Sichuan China

**Keywords:** Lymphopoiesis, T cells

Correction to: *Cell Death and Disease* 10.1038/s41419-025-07392-8, published online 10 February 2025

Figure 5Cc2 and the middle image in the upper row of Figure 6H, Figure 5Hh1 and Figure S15Cc1, the images in these two groups were unintentionally duplicated during figure preparation. These errors occurred due to an oversight when we arranged the individual image in Figure 5, and did not involve any manipulation or alteration of the original data. Importantly, as stated in the article, both mcm5 and mcm3 mutations result in similar T-cell developmental phenotype. In addition, Figure 5Hh1 and Figure S15Cc1 all showed the T cell phenotype in controls. Thus, our unintentional duplication does not compromise the integrity of the dataset and the conclusions of the study.

**Original Fig 5** 
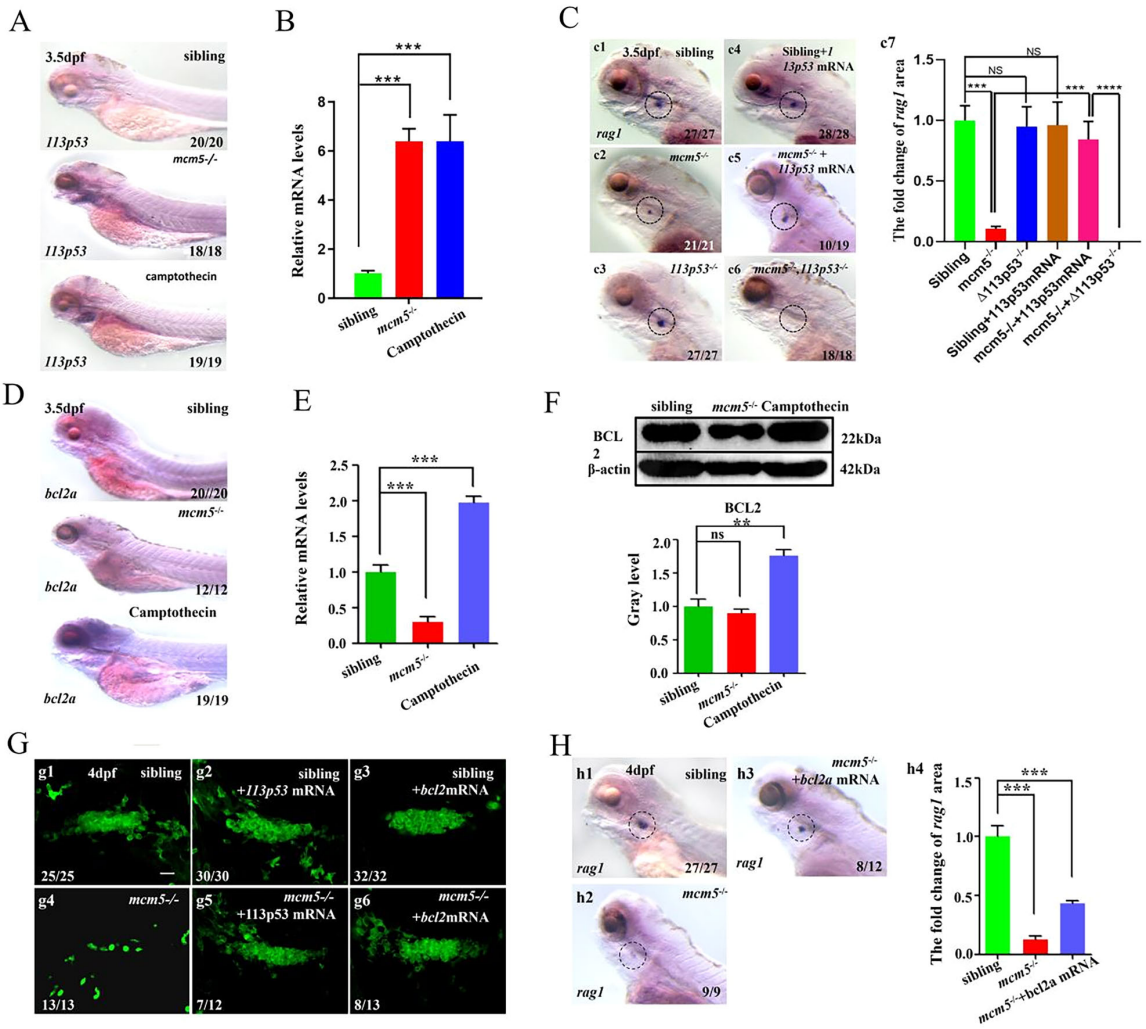


**Original data Fig5C-c2(not cut)** 
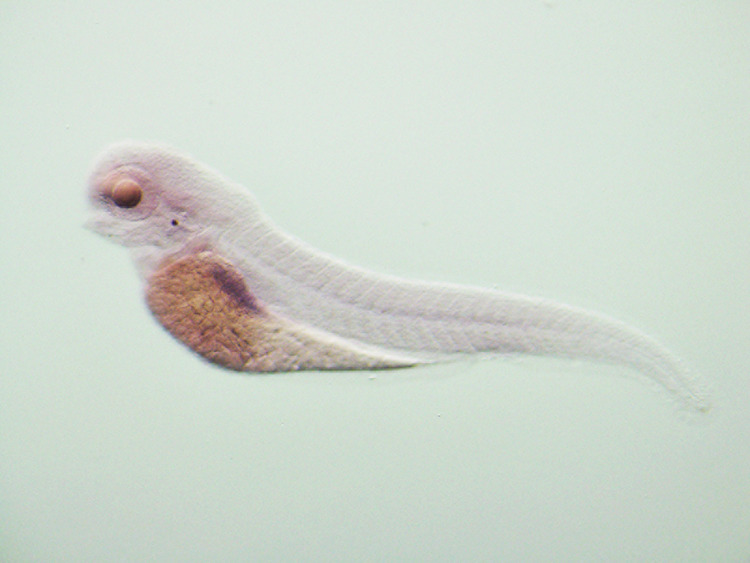


**Original data Fig5H-h1(not cut)** 
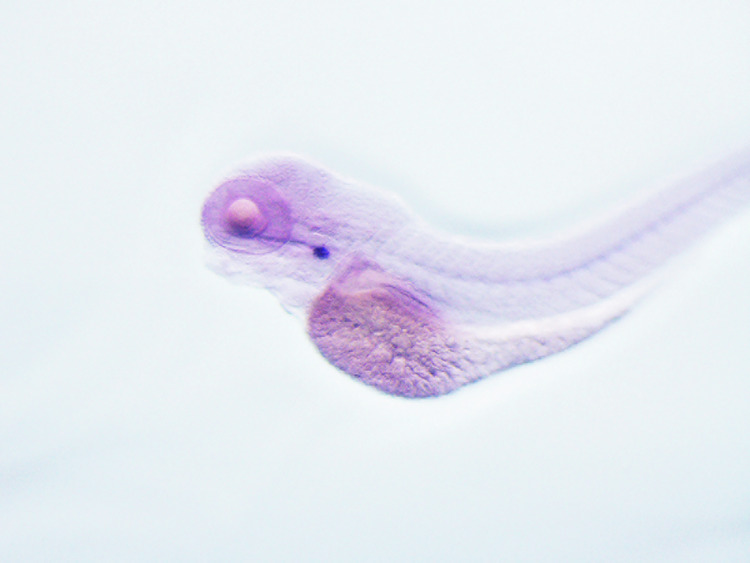


**Amended Fig. 5** 
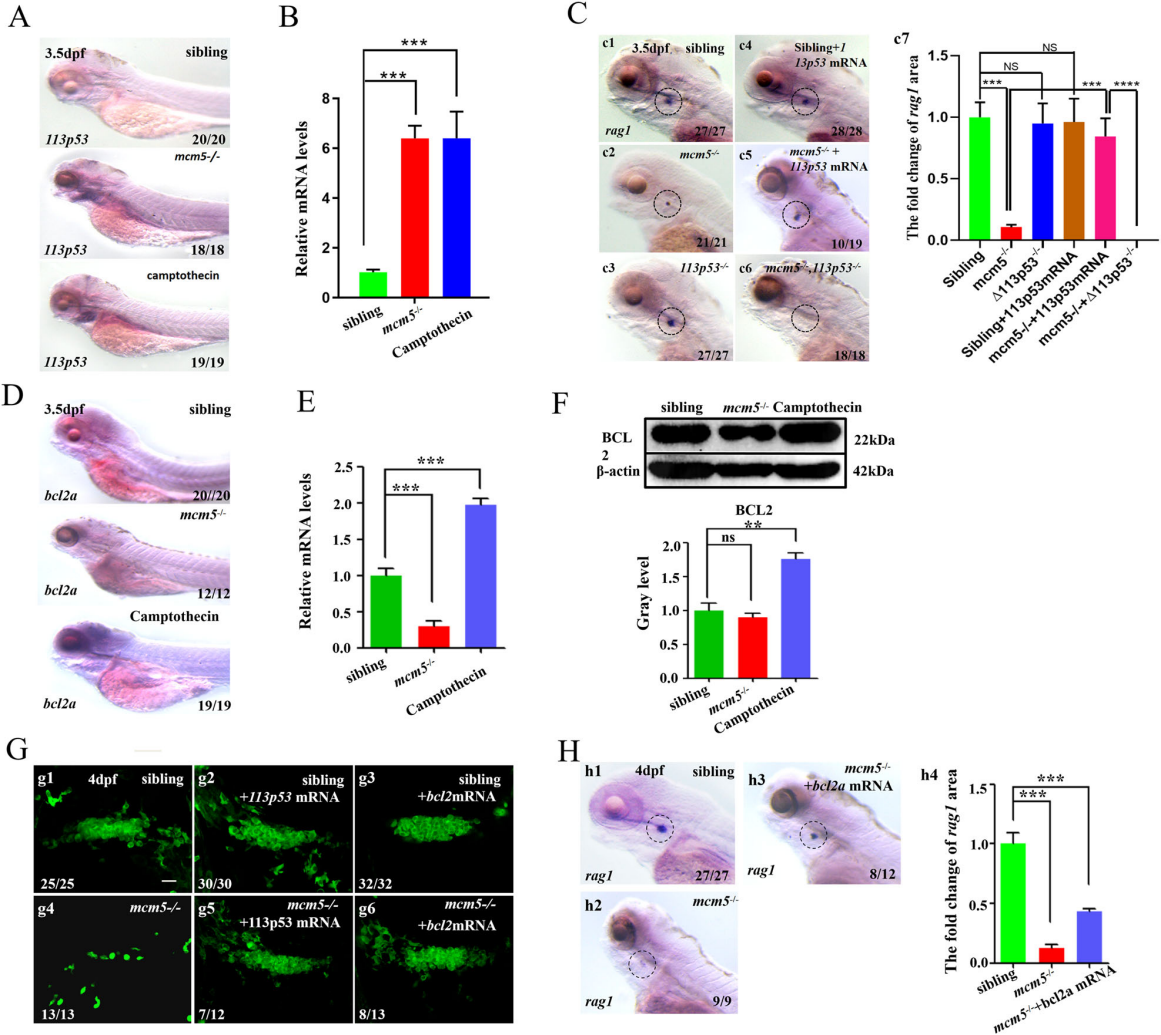


**Amended Fig5C-c2 (cut)** 
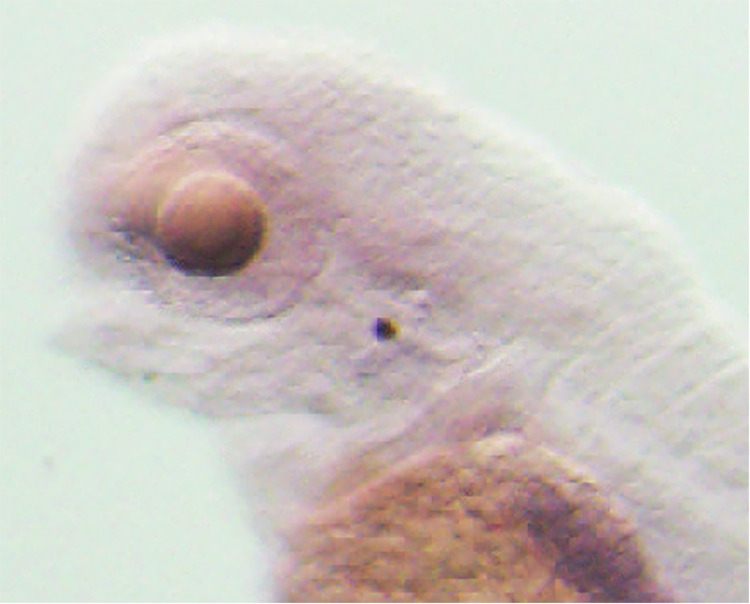


**Amended Fig5H-h1(cut)** 
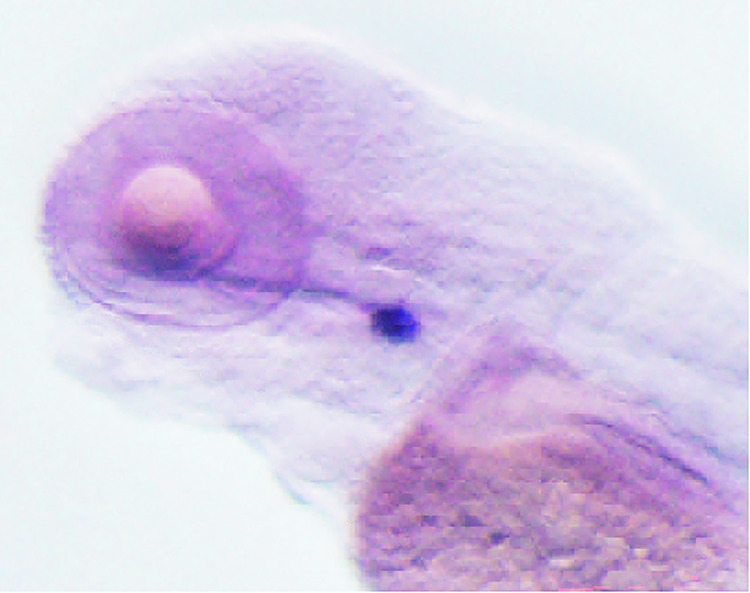


The original article has been corrected.

