# Supplementary material for: Mcm5 mutation leads to silencing of Stat1-bcl2 which accelerating apoptosis of immature T lymphocytes with DNA damage
Source: Cell Death Dis. 2025 Feb 10;16(1):84. doi: 10.1038/s41419-025-07392-8 (PMC11811017; doi:10.1038/s41419-025-07392-8)
Supplement: Supplementary file 1 — Supplementary information [file 41419_2025_7392_MOESM1_ESM.docx]

**Supplementary information**

**Figure S1-20 and Figure S legends:**

**
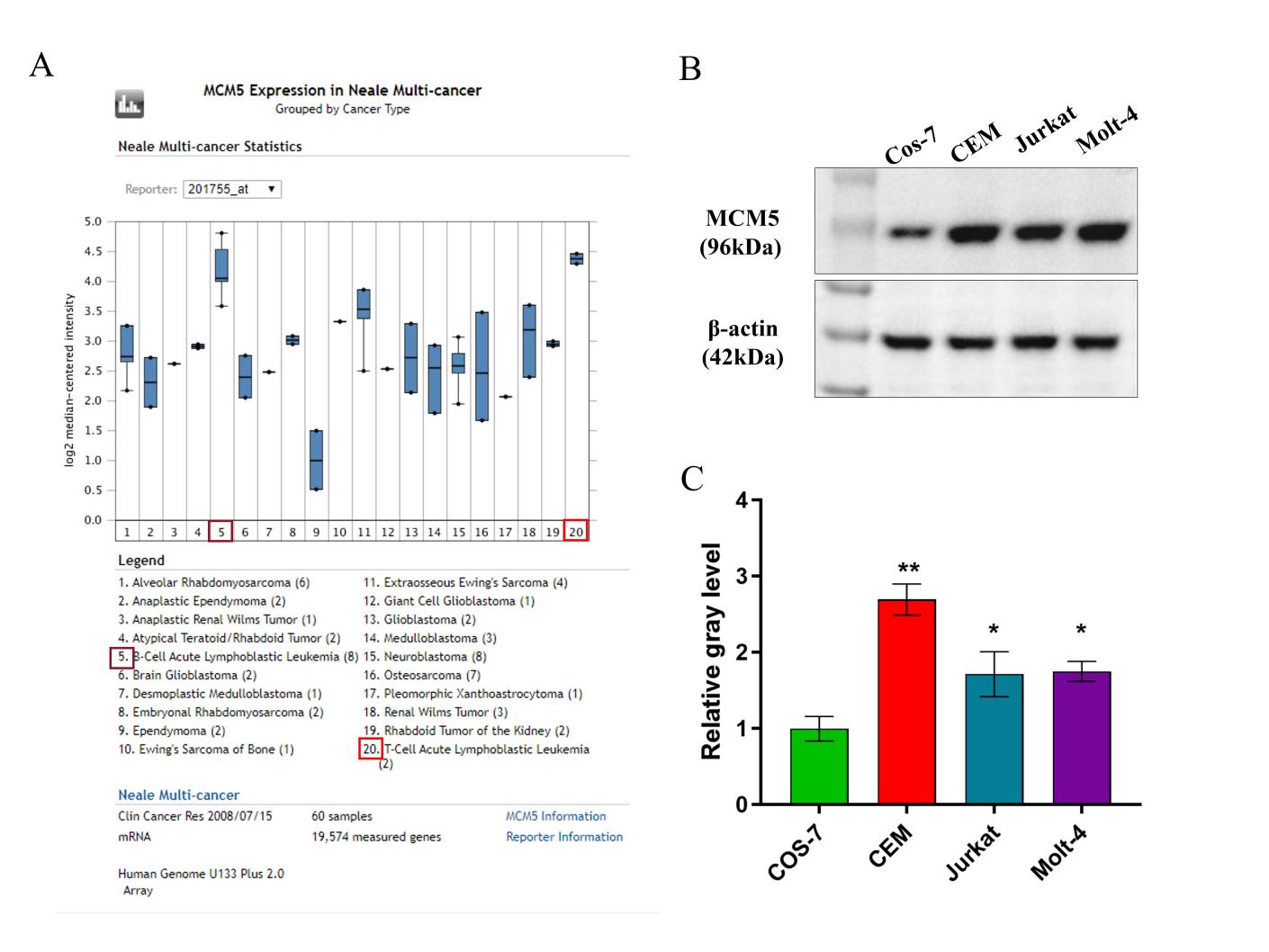
Fig. S1 MCM5 expression in cancer and cell lines.**

A. 1.Alveolar Rhabdomyosarcoma; 2.Anaplastic Ependymoma; 3.Anaplastic Renal Wilms Tumor; 4. Atypical Teratoid/Rhabdomyoid tumor; 5.B-cell lymphoblastic Acute leukemia (deep red box);Glioblastoma; 6.Brain Glioblastoma; 7.Desmoplastic medulloblastoma; 8.Embryonial Rhabdomyosarcoma; 9.Ependymoma; 10.Ewing's Sarcoma of Bone; 11.Extraosseous Ewing's Sarcoma; 12.Giant Cell Glioblastoma; 13.Glioblastoma; 14.Medulloblastoma; 15.Neuroblastoma; 16.Osteosarcoma; 17.Pleomorphic Xanthoastrocytoma; 18.Renal Wilms Tumor; 19. Rhabdooid Tumor of the Kidney; 20. T-cell Acute lymphoblastic leukemia (red box). B: The protein level of MCM5 in cell lines was detected by WB assay. Renal fibroblasts (COS-7), human acute T lymphocytic leukemia cell line (CEM, Jurkat, Molt4); C: Use ImageJ software to analyze the protein level of MCM5. For b, the data were presented as means ± SD; The P values (t-test; two-tailed) “*” P＜0.05; “**” P＜0.01.

**
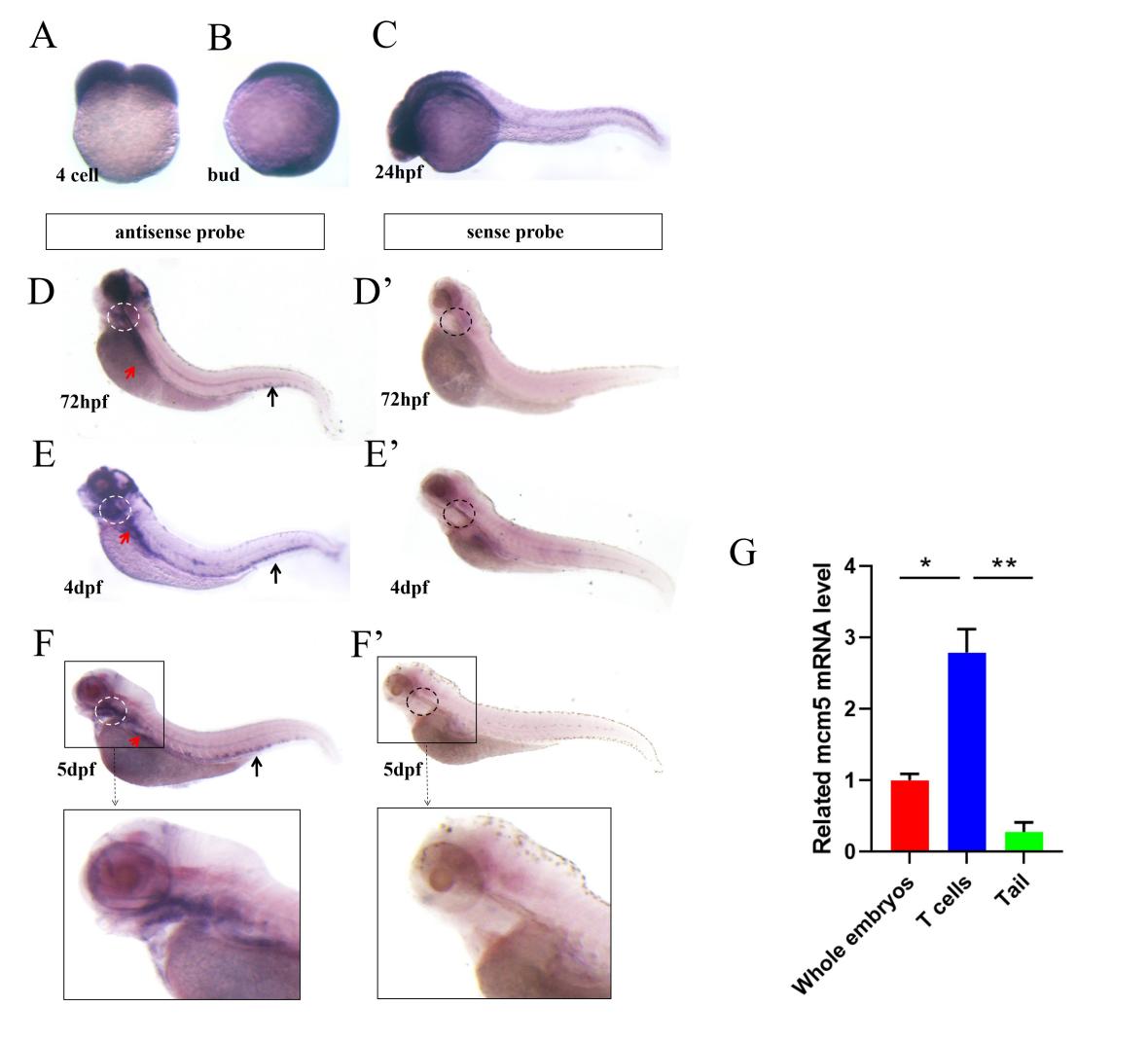
Fig. S2 Expression of *mcm5* at different developmental stage and in different tissues**

(A) Expression of *mcm5* indicated *mcm5* was a maternal expressed gene. (B) *mcm5* was expressed ubiquitously at bud stage. (C) At 24 hours post fertilization (hpf), *mcm5* was restricted in head eyes and endoderm. (D-F) The expression of *mcm5* was restricted in eyes, the boundary of hindbrain and midbrain, gills and thymus area (white/black circles showed), endoderm organ (red arrow showed) and caudal hematopoietic tissue (CHT) (black arrow showed). (G) Immature T cells was sorted at 5 dpf, then the whole embryos, the T cells and the tails were used to examine the relative level of *mcm5* expression using RT-qPCR. The level of *mcm5* expression in T cells was higher than that in tails and whole embryos. For g, the data was presented as means ± SD; The P values (t-test; two-tailed), “*” P＜0.05; “**” P＜0.01.

**
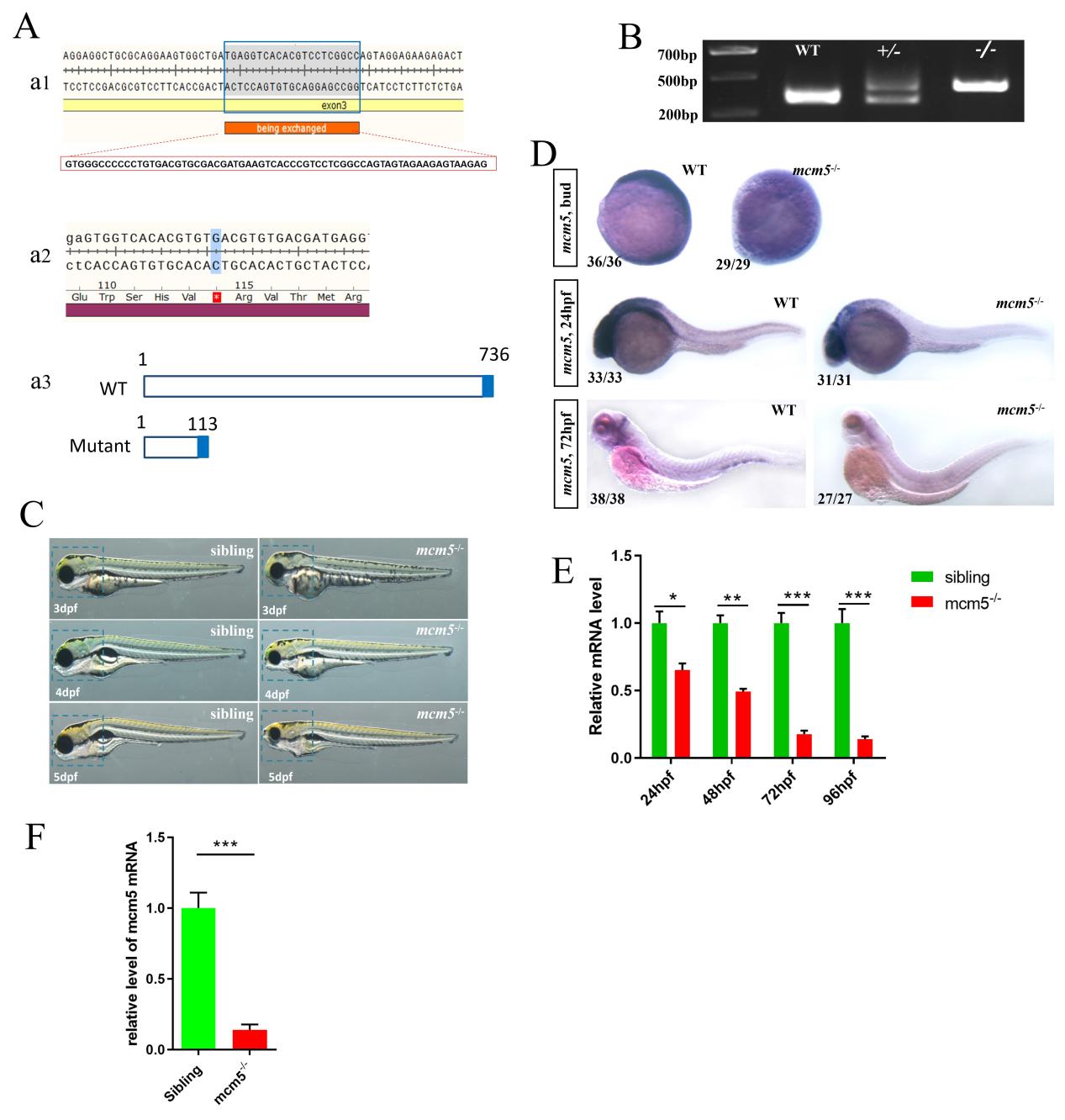
Fig. S3 The related information about *mcm5* mutants**

1. In the exon3 of *mcm5* gene, 23bp nucleotides was exchanged with another 65 bp nucleotide (a1, showed on the left, blue box), which results in pre-stop codon in the CDs region (a2) and a truncated MCM5 protein (a3). (B) PCR amplification of the genomic DNA in wild type embryos (a single and short band), heterozygotes (a longer band and a short band) and homozygotes embryos (a single longer band). (C) Living embryos of sibling and *mcm5^-/-^* embryos at 3dpf, 4dpf and 5dpf. The eye and the head in *mcm5^-/-^* embryos was smaller (red box showed), and the swimming bladder was disappeared in *mcm5^-/-^* embryos. (D) The expression of *mcm5* mRNA was greatly downregulated in *mcm5^-/-^* embryos at bud stage and 24hpf, even disappeared in eyes, boundary of hindbrain and midbrain, thymus area and CHT region at 3dpf. (E) RT-qPCR was used to examine the level of *mcm5* mRNA in controls and *mcm5^-/-^* embryos, the control siblings are wild type embryos. (F) RT-qPCR was used to examine the level of *mcm5* mRNA in thymus region in controls and *mcm5^-/-^* embryos. For e and f, the data were presented as means ± SD; The P values (t-test; two-tailed), “*” P＜0.05; “**” P＜0.01; “***” P＜0.001.


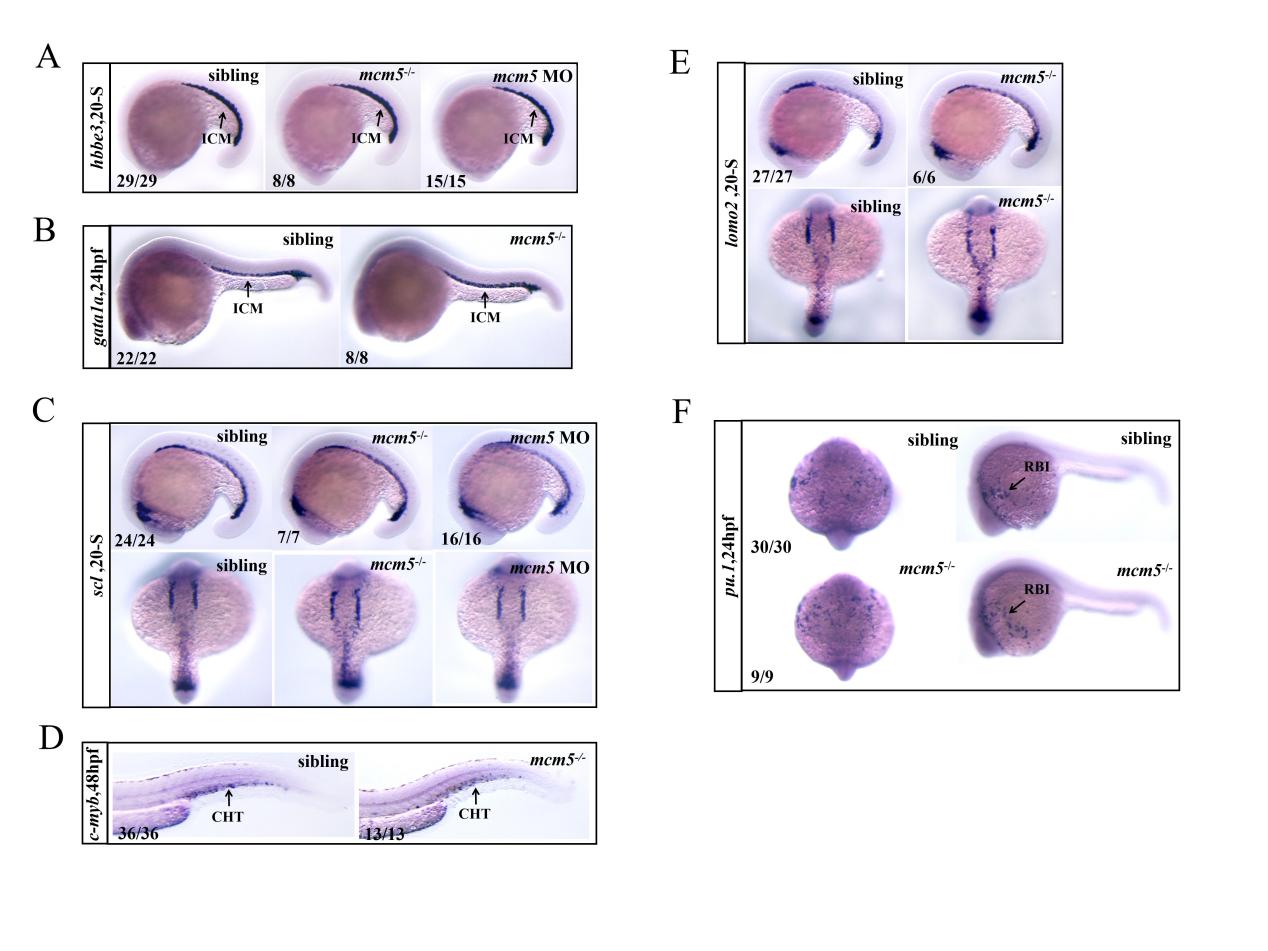


**Fig. S4 Mcm5 loss of function does not influence the primary hematopoiesis at early stage**

(A-B) The expression of the erythrocyte markers *hbbe3* and *gata1a* were examined. (A) No difference was observed for the expression of *hbbe3* in controls (n=29), *mcm5^-/-^* embryos (n=8) and *mcm5* morphants (n=15) at 20 Somite stage (SS) (A). Expression of *gata1a* in siblings (n=22) and *mcm5^-/-^* embryos (n=8) was examined at 24 hpf (B), there is no difference between these two groups of embryos. (C-D) The expression of the HSCs markers *scl* and *c-myb* were examined. Expression of *scl* in siblings (n=24), *mcm5^-/-^* (n=7) and *mcm5* morphants (n=16) was similar at 20 SS(C). Expression of *c-myb* in siblings (n=36) and *mcm5^-/-^* embryos (n=13) was same at 48hpf (D). (E) Expression of *lmo2,* the primitive hematopoietic regulator, was checked in siblings (n=27) and *mcm5^-/-^*embryos (n=6) at 20 SS, there is no difference between them. (F) Myelopoiesis regulator *pu.1* was examined and the expression of *pu.1* in siblings (n=30) and *mcm5^-/-^* embryos (n=9) was similar at 24hpf.

**
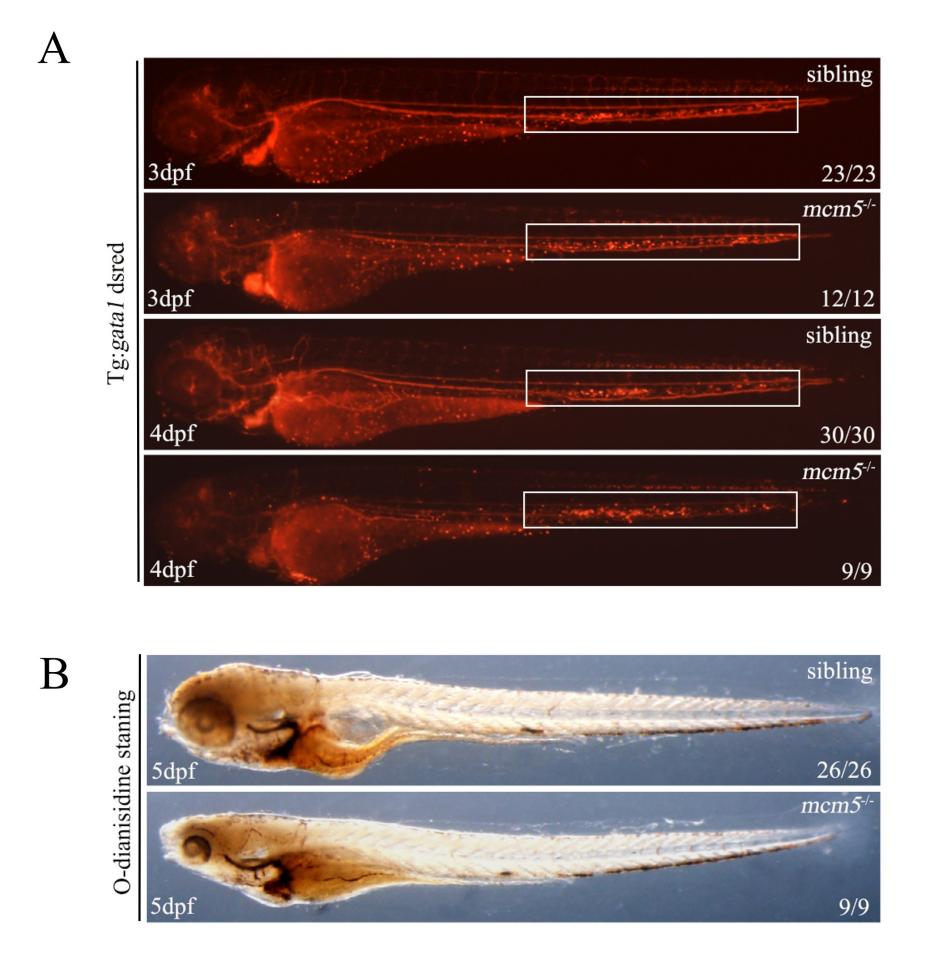
**

**Fig. S5 The role of *mcm5* was not observed in erythropoiesis**

(A) Erythrocyte development was evaluated at 3dpf and 4dpf for siblings and *mcm5^-/-^* embryos in *Tg(gata1a: desRed)* transgenic line background. (B) Erythrocyte staining with o-dianisidine showed no different in siblings and *mcm5^-/-^* embryos at 5dpf.


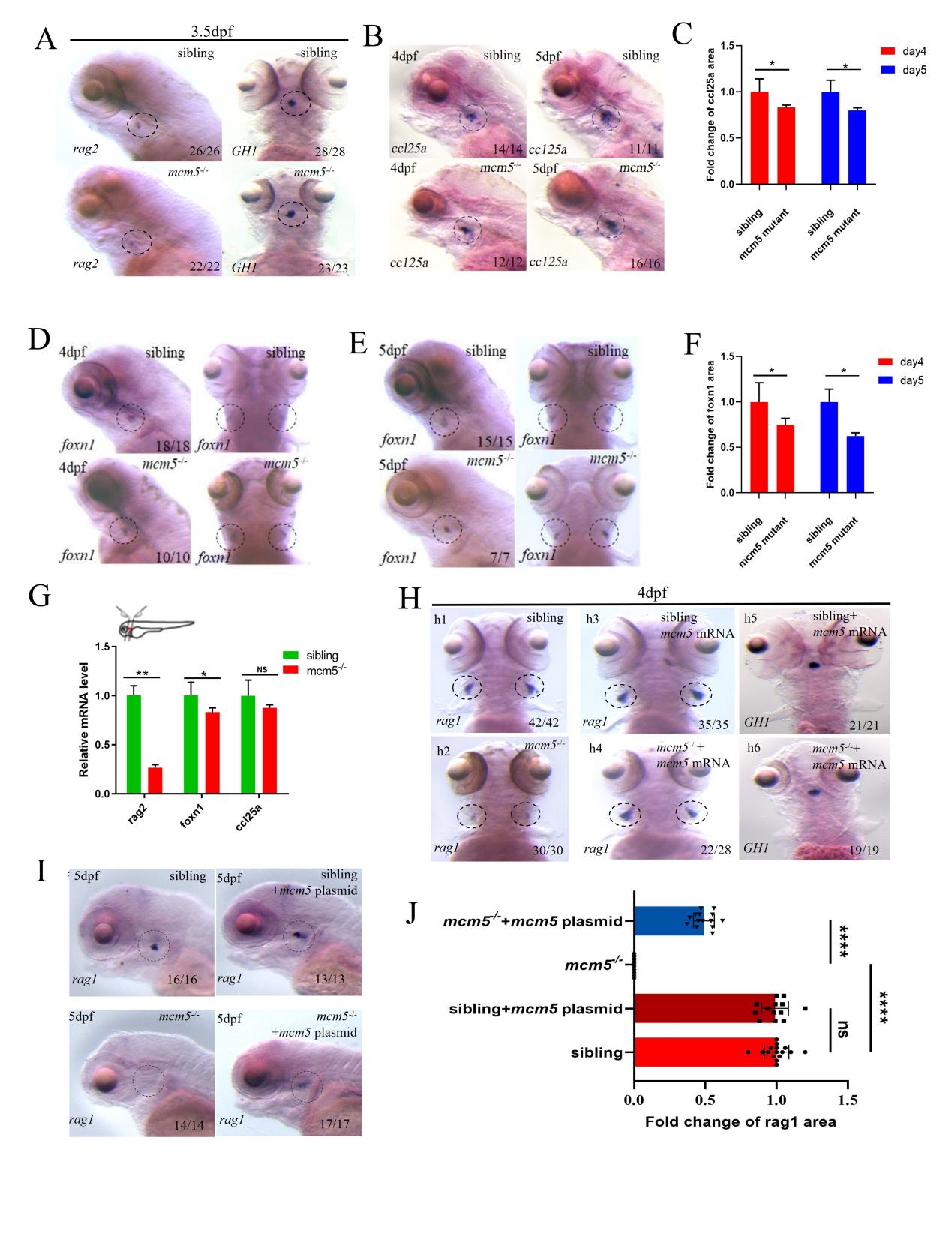
 **Fig. S6 Immature T cell development was disturbed in mcm5 mutants**

1. The expression of ***rag2*** and ***CH1*** was examined in siblings and ***mcm5^-/-^*** embryos at 3,5 dpf, no differences in the expression of ***rag2*** and ***CH1*** were observed between siblings and ***mcm5^-/-^*** embryos. (B) Expression of *ccl25a* in siblings and *mcm5^-/-^* embryos at 4dpf and 5dpf. The quantification analysis showed that the area of *ccl25a* expression was decreased in *mcm5* mutants (C). (D-E)The area of *foxn1* expression was decreased at 4dpf (D) and 5dpf (E). (F) The quantification analysis showed that the area of *foxn1* expression was decreased in *mcm5* mutants. (G) The thymus and the around region was cut and collected, which was used to examine the expression level of *rag2*, *foxn1* and *ccl25a*. The level of *rag2* expression was greatly decreased, the level of *foxn1* expression was mild decreased. (H) At 4 dpf, the expression of ***rag1*** was reduced in ***mcm5^-/-^*** embryos compared to siblings (h1-h2). Injection of ***mcm5*** mRNA rescued the expression of ***rag1*** in ***mcm5^-/-^*** embryos (h3-h4). Overexpression of ***mcm5*** mRNA did not affect the expression of ***CH1*** in either siblings or ***mcm5^-/-^*** embryos (h5-h6). (I) After thymus-specific overexpression of ***mcm5*** mRNA, the expression of ***rag1*** in ***mcm5*** mutant embryos was partially rescued at 5 dpf. (J) The quantification analysis showed that the area of *rag1* expression was partially rescued in *mcm5* mutants. For C, F,G,and J, the data were presented as means ± SD; The P values (t-test; two-tailed); “*” P＜0.05; “**” P＜0.01; “****” P < 0.0001.

**
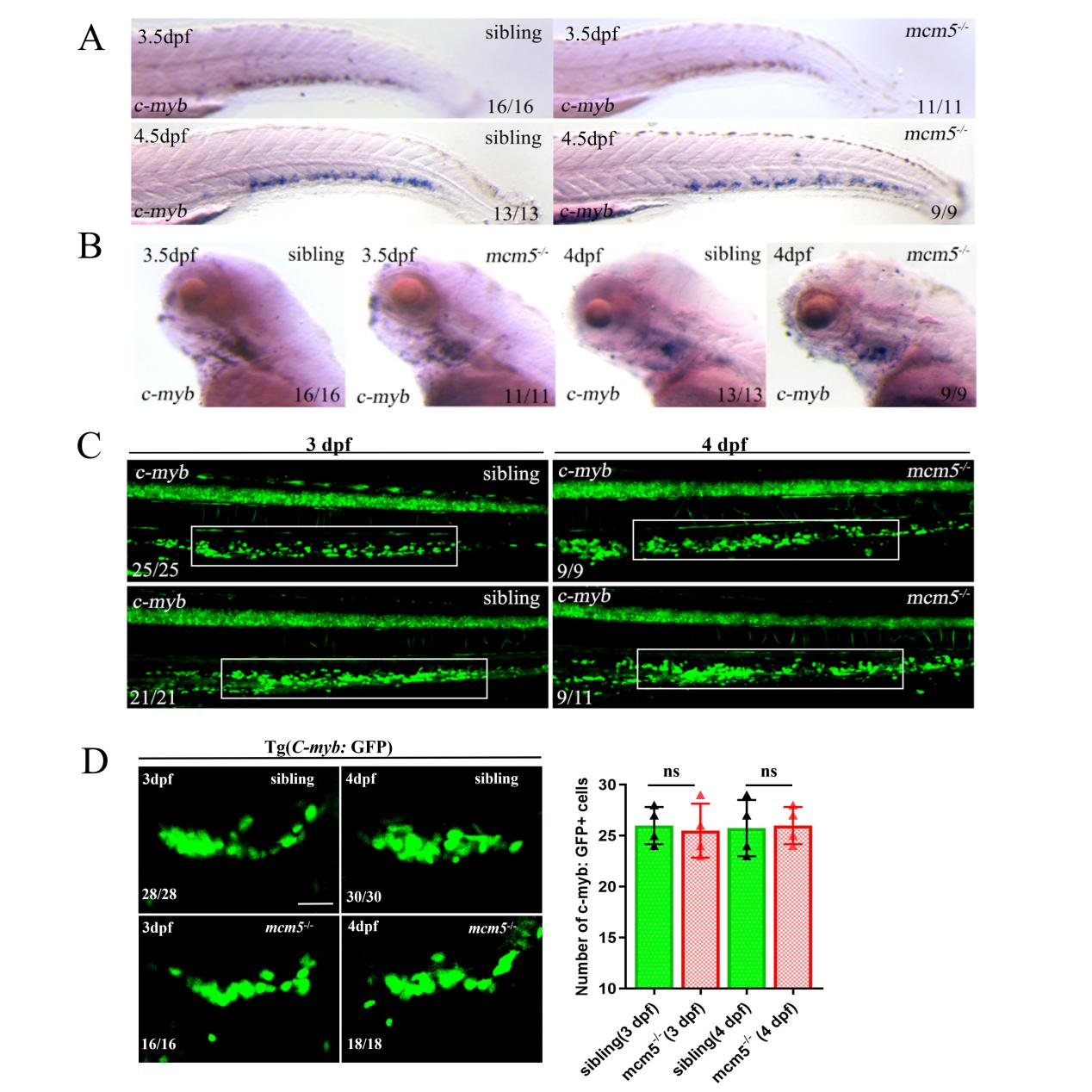
**

**Fig. S7 HSCs development was not disturbed greatly in *mcm5^-/-^*embryos.**

(A-B) HSCs were analyzed in siblings and *mcm5^-/-^* embryo using *in situ* experiments at 3.5dpf and 4.5dpf. No big difference was observed for the expression of *c-myb* in the CHT (A) and thymus area (B) in siblings and *mcm5^-/-^* embryos at 3.5dpf and 4.5dpf. (C-D) The HSCs in the CHT (C) and thymus area (D) were examined using *Tg (c-myb:GFP*) transgenic line, no distinct difference was observed in siblings and *mcm5^-/-^* embryos at 3dpf and 4dpf. Scale bars, 40μm. For D, the data was presented as means ± SD; The P values (t-test; two-tailed), NS, not significant.

**
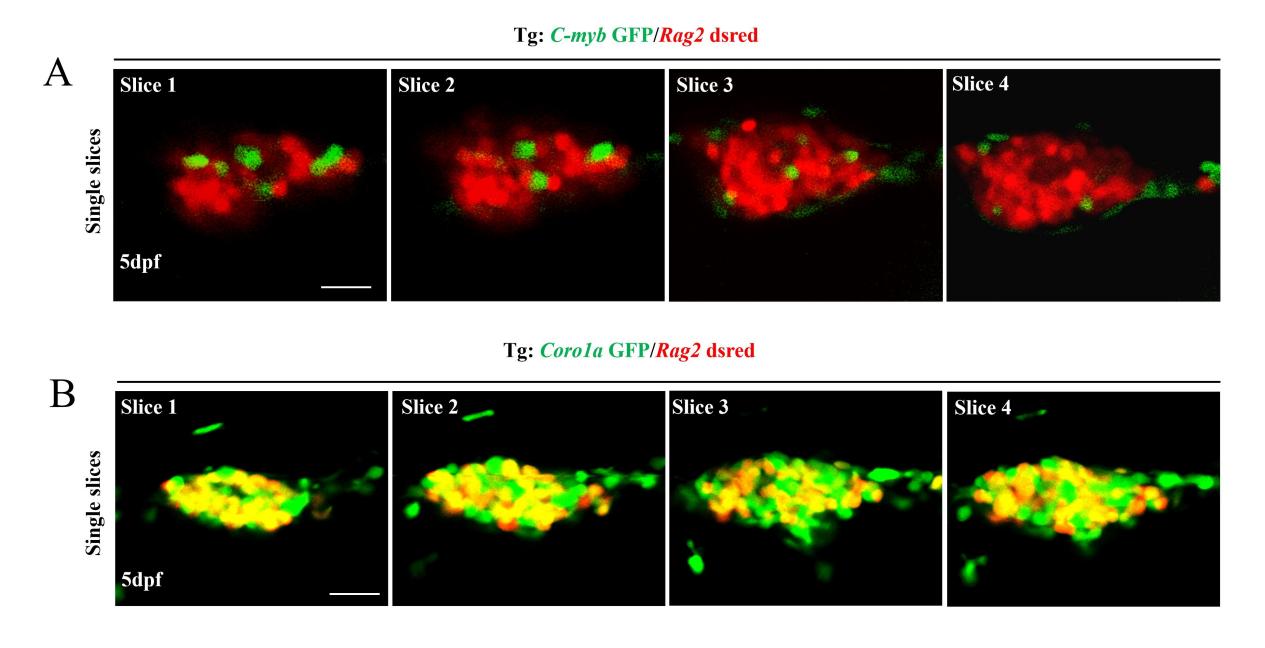
Fig. S8 Most of immature T cells was labeled with *Coro1a*:GFP positive cells**

(A) Live image of *rag2:desRed* positive immature T cells (Red cells) and *c-myb:GFP* positive HSCs in different single slices in Fig. 2B. Only small part of immature T cells was labeled with GFP (yellow column, 3%). (b) Live image of *rag2:desRed* positive immature T cells and *coro1a:GFP* positive cells in single slices in Fig. 2C. 82.4% of desRed positive cells were overlapped with GFP labeled cells (yellow column). Scale bars, 30μm.

**
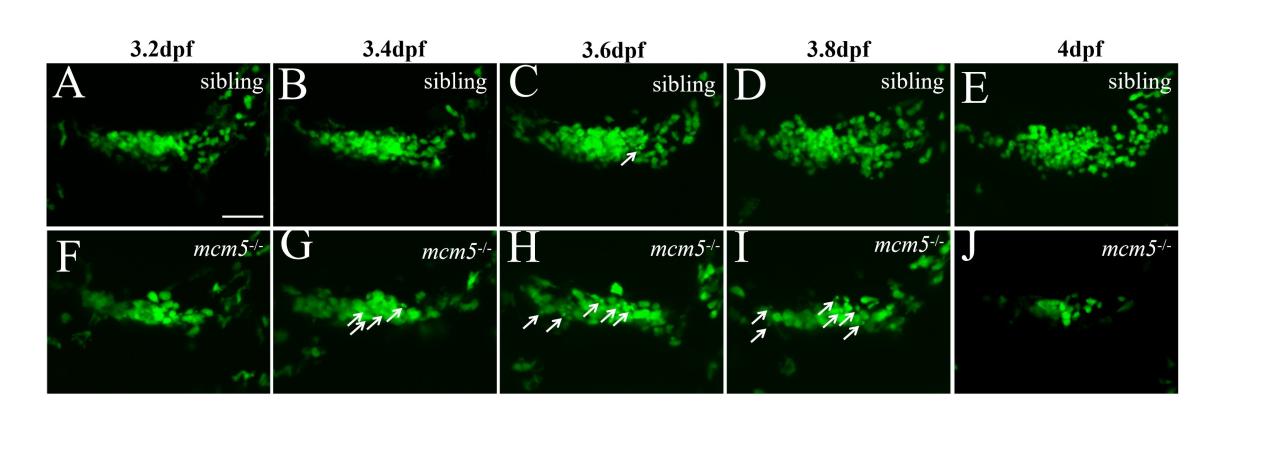
**

**Fig. S9 Live image of *coro1a:GFP* labeled cells in *Tg(coro1a:GFP)* embryo.**

(A-E) Live image of *coro1a:GFP* cells in siblings. From 3.2dpf to 4dpf, the *coro1a:GFP* labeled cells increased (A-E), only occasional apoptosis like cell appeared (C, D, arrow showed). (F-J) Live image of *coro1a:GFP* cells in *mcm5^-/-^* embryos. Comparing with the siblings, the *coro1a:GFP* cell number was not significant different before 3.4dpf (G), but from this stage and later, more *coro1a:GFP* cells displayed apoptosis-like phenotype (G-I, arrow showed), and the *coro1a:GFP* cell number decreased (H-J). Scale bars, 40μm.

**
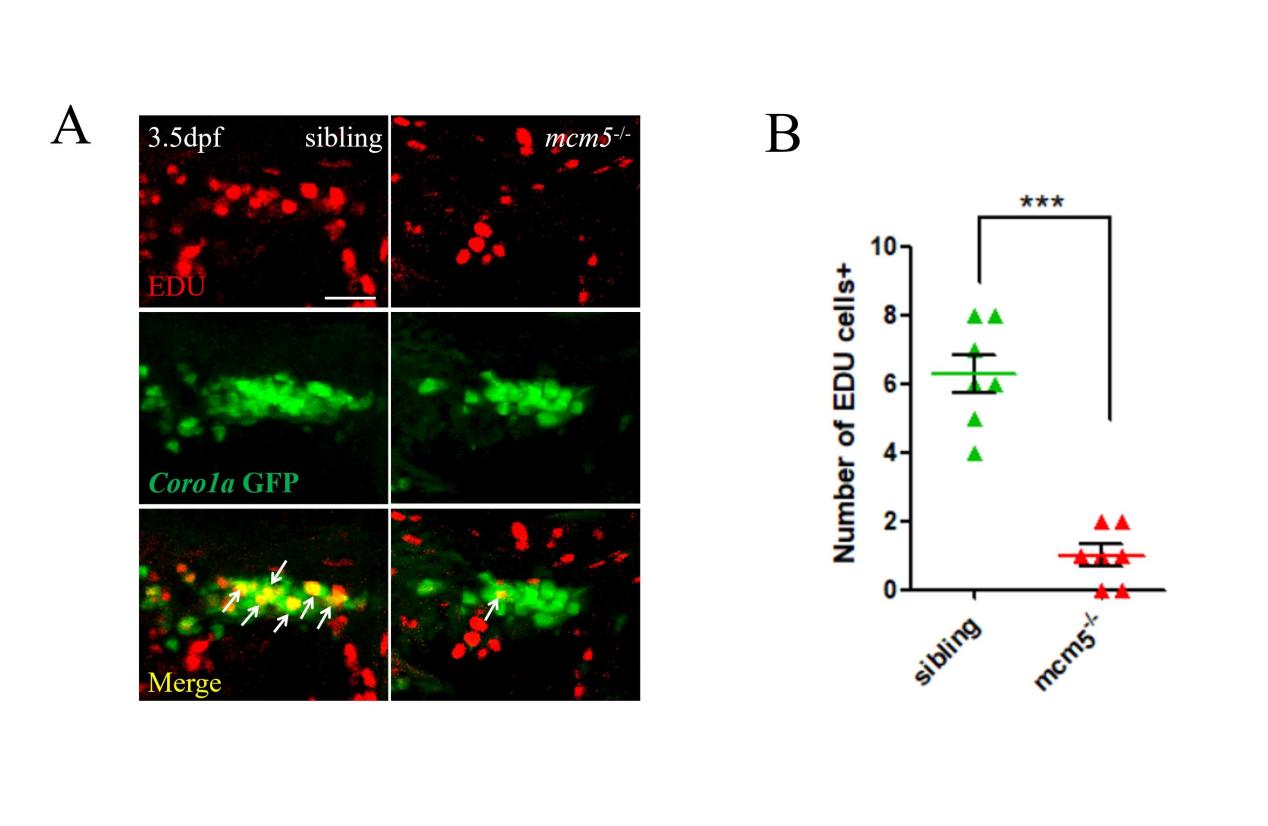
**

**Fig. S10 The Edu incorporation assay.**

(A) EdU incorporation was assessed in siblings (left showed) and *mcm5^-/-^*embryos (right showed) at 3.5dpf. (B) [Statistic](javascript:;) [analysis](javascript:;) showed that the *coro1a:GFP* cell labeled with EdU was significantly decreased in *mcm5^-/-^* embryos (n=7 for siblings and mutants). For B, the data was presented as means ± SD; The P values (t-test; two-tailed); “***” P＜0.001. Scale bars, 40μm.

**
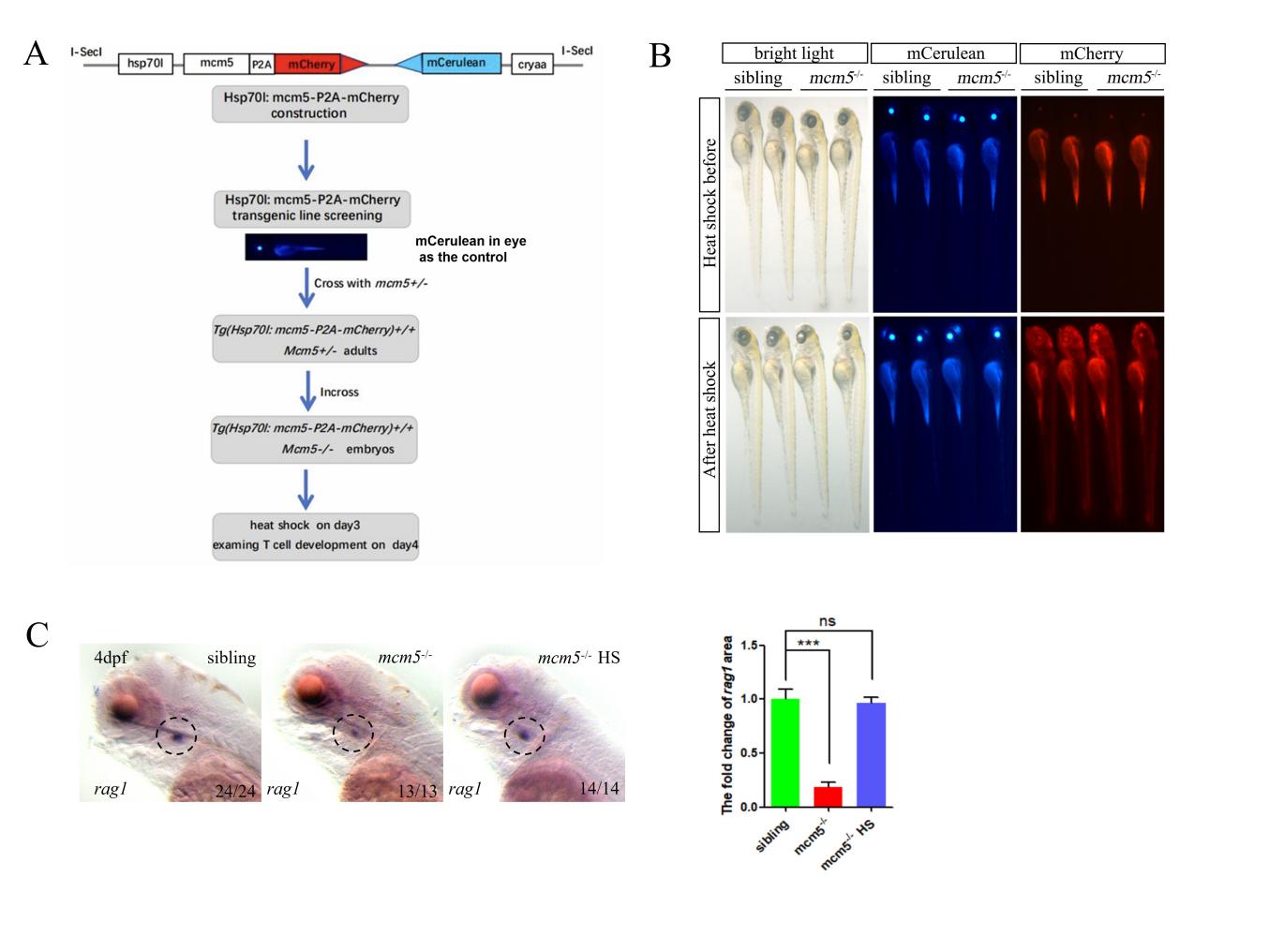
**

**Fig. S11 Induced expression of *mcm5* on 3dpf rescued the T lymphocyte developmental defect in *mcm5^-/-^* embryos.**

(A) Schematic for the construction of the transgenic line *Tg(Hsp70l:mcm5-T2A-mCherry)*, and how to use it to carry out rescue experiment in specific stage. The mCerulean in eyes is used as a control, the embryos with mCerulean in eyes are *Tg(Hsp70l:mcm5-T2A-mCherry)* transgenic embryos. (B) MCM5-T2A-mCherry was extensively induced to express using heat-shock at 3dpf. Before heat shock, the autofluorescence (red) exists in the yolk of embryos. After heat shock, MCM5-P2A-mcherry was induced to express in the whole body, including head, thymus region and truck region. (C) The expression of *rag1* was rescued at 4dpf by overexpression of *mcm5*. For C, the data was presented as means ± SD; The P values (t-test; two-tailed); “NS”, not significant; “***” P < 0.001.

**
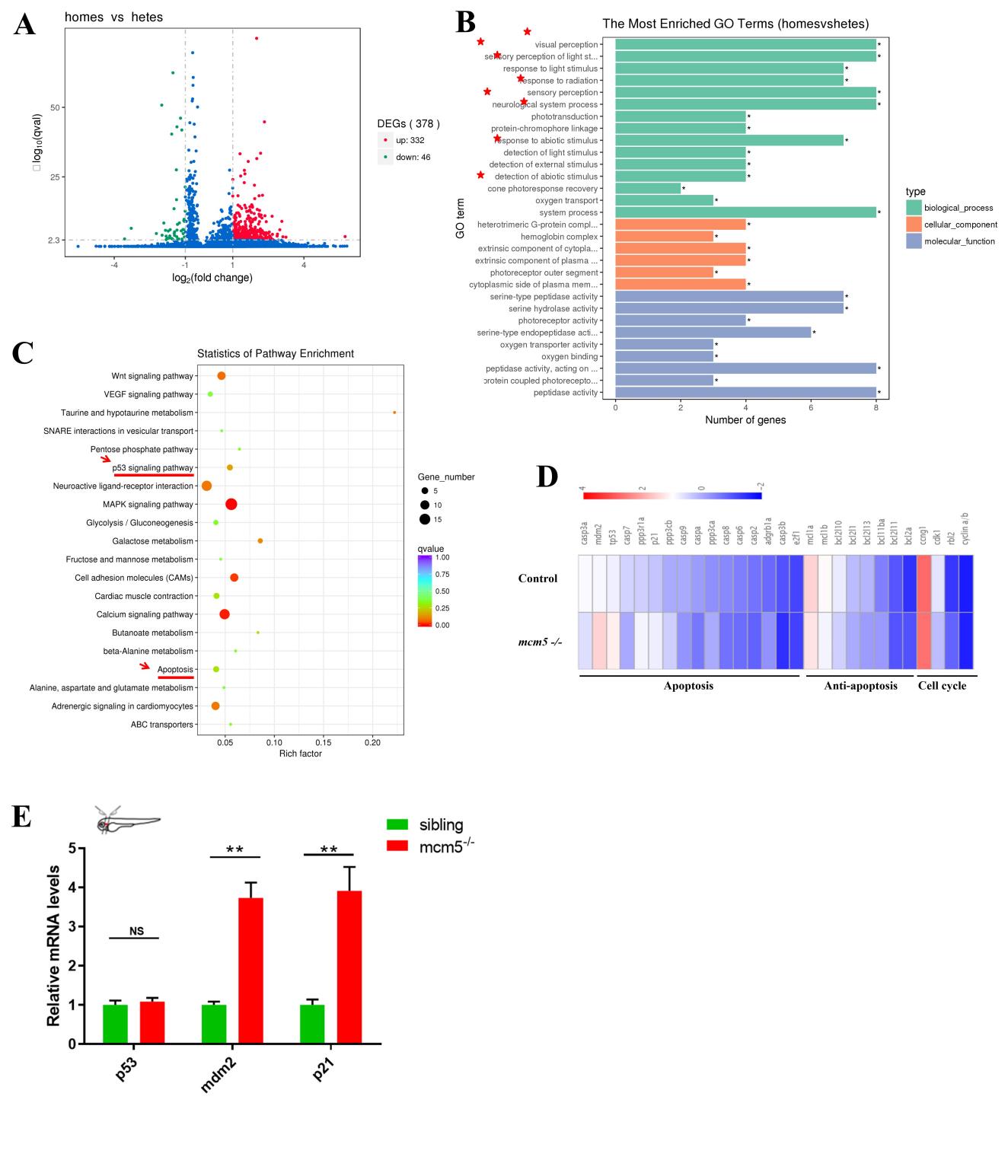
Fig. S12 The transcription analysis in *mcm5* mutants and control embryos**

(A) In comparing with the controls, 332 genes and 46 genes were up/down regulated significantly in *mcm5* mutant embryos. (B) In the down-regulated genes, many of them were neural related genes, especial the genes being related to eye development and function (8/15 of the clusters in biological process). (C) Pathway enrichment analysis showed that the *p53* signal and cell apoptosis signal were both up-regulated (red arrows and red lines showed). (D) Detailed analysis of some genes in *p53* signal and cell cycle regulation, the apoptosis and the anti-apoptosis related genes were both up-regulated, as well the cell cycle related genes were also changed. (E) The thymus region and the region around thymus were cut from *mcm5* mutants and siblings, which were used to evaluate the expression difference of *tp21* and *mdm2* between *mcm5* mutant and siblings using RT-qPCR. The data showed that *tp53* signaling downstream gene *tp21* and *mdm2* was also upregulated in thymus region of *mcm5* mutants. For e, the data was presented as means ± SD; The P values (t-test; two-tailed); “NS”, not significant; “**” P < 0.01.

**
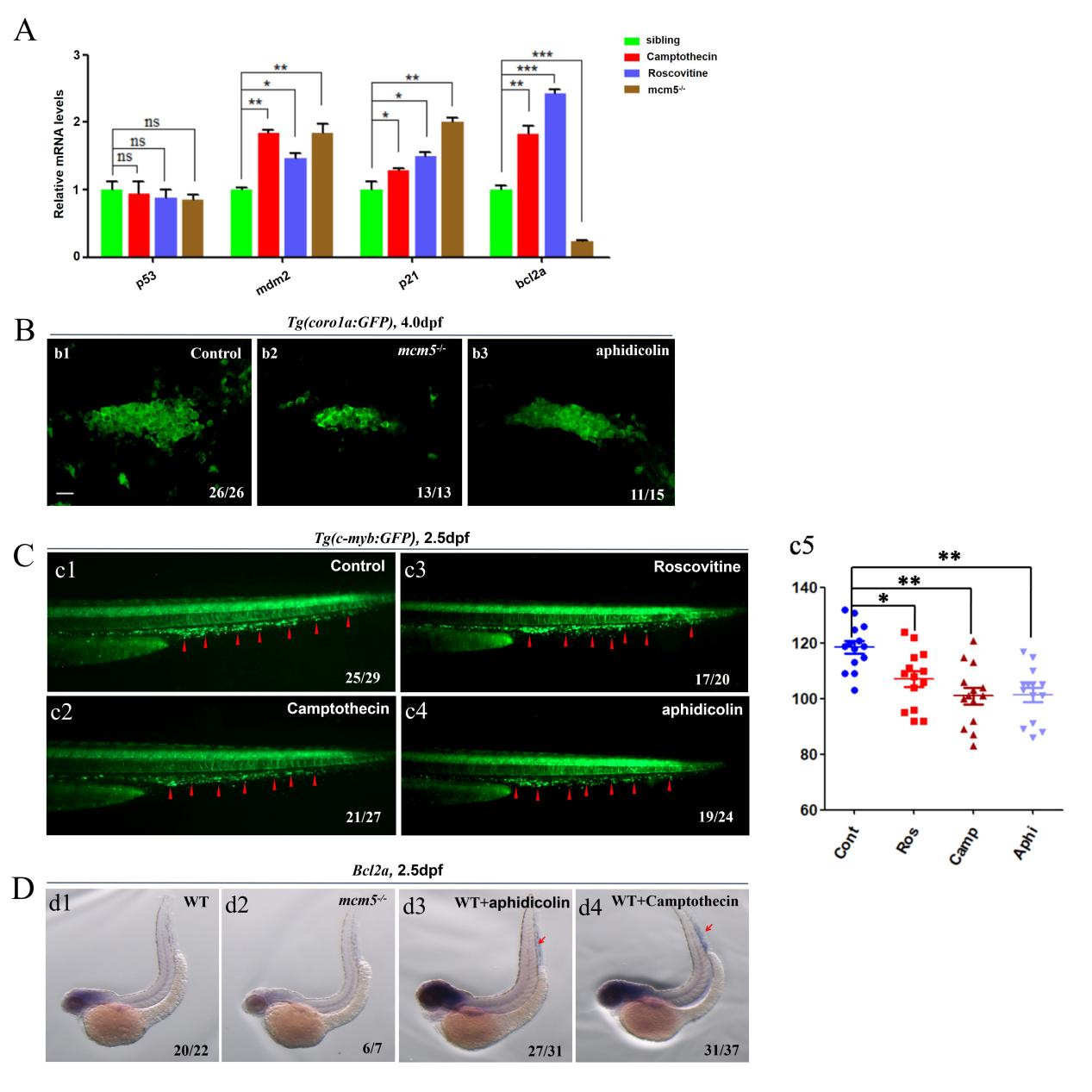
Fig. S13 Treatment with different chemicals resulted in mild T cell development defect.**

(A) The transcription level was examined using RT-qPCR for *p53*, *mdm2*, *p21* and *bcl2a*. Expression of *mdm2* and *p21* were upregulated in *mcm5^-/-^* embryos and chemical treatment embryos; Expression of *bcl2a* was upregulated in chemical treatment embryos, but decreased in *mcm5^-/-^* embryos. (B) Comparing with controls (b1), after treating with chemicals from 1.5 dpf to 4 dpf the coro:GFP labeled T cells was decreased in embryos treated with aphidicolin (b3), but not as strong as in that of *mcm5^-/-^* embryos (b2). (C) c-myb:GFP labeled HSCs was examined in embryos treated with different chemicals from 1.5 dpf to 2.5 dpf (red arrow heads showed). The data showed that in CHT region the member of HSCs was mild decreased (b2-b5). (D) Expression of *bcl2a* in different kind of embryos at 2.5 dpf after treating with chemicals from 1.5 dpf to 2.5 dpf. Comparing with controls (d1), the expression of *bcl2a* was decreased in *mcm5^-/-^*embryos (d2), the expression of *bcl2a* was increased in embryos treated with aphidicolin or camptothecin, including in the CHT region (d3, d4, red arrow showed). The data was presented as means ± SD; The P values (t-test; two-tailed); NS, not significant. “*” P < 0.05, “**” P < 0.01, “***” P < 0.001.


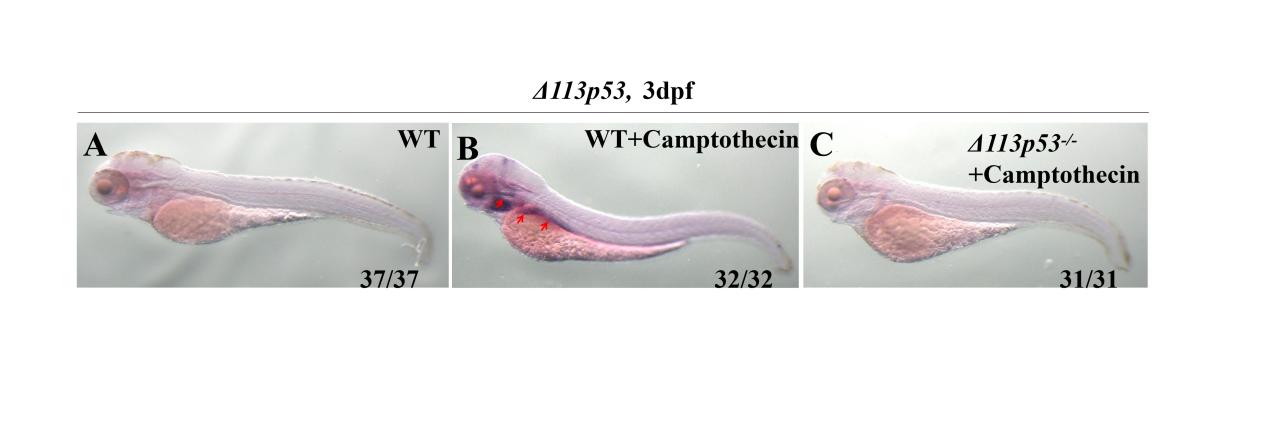


**Fig. S14 Expression of *Δ113p53* could not be increased in *Δ113p53^-/-^* embryos when campothecin treatment was applied**

(A) In wild type embryos, no expression of *Δ113p53* was observed in thymus, endodermal organs and the boundary of midbrain and hindbrain. (B) While its expression in these regions was upregulated in embryos treated with campothecin (red arrows showed). (C) *Δ113p53* expression was not observed in *Δ113p53^-/-^* embryos.

**
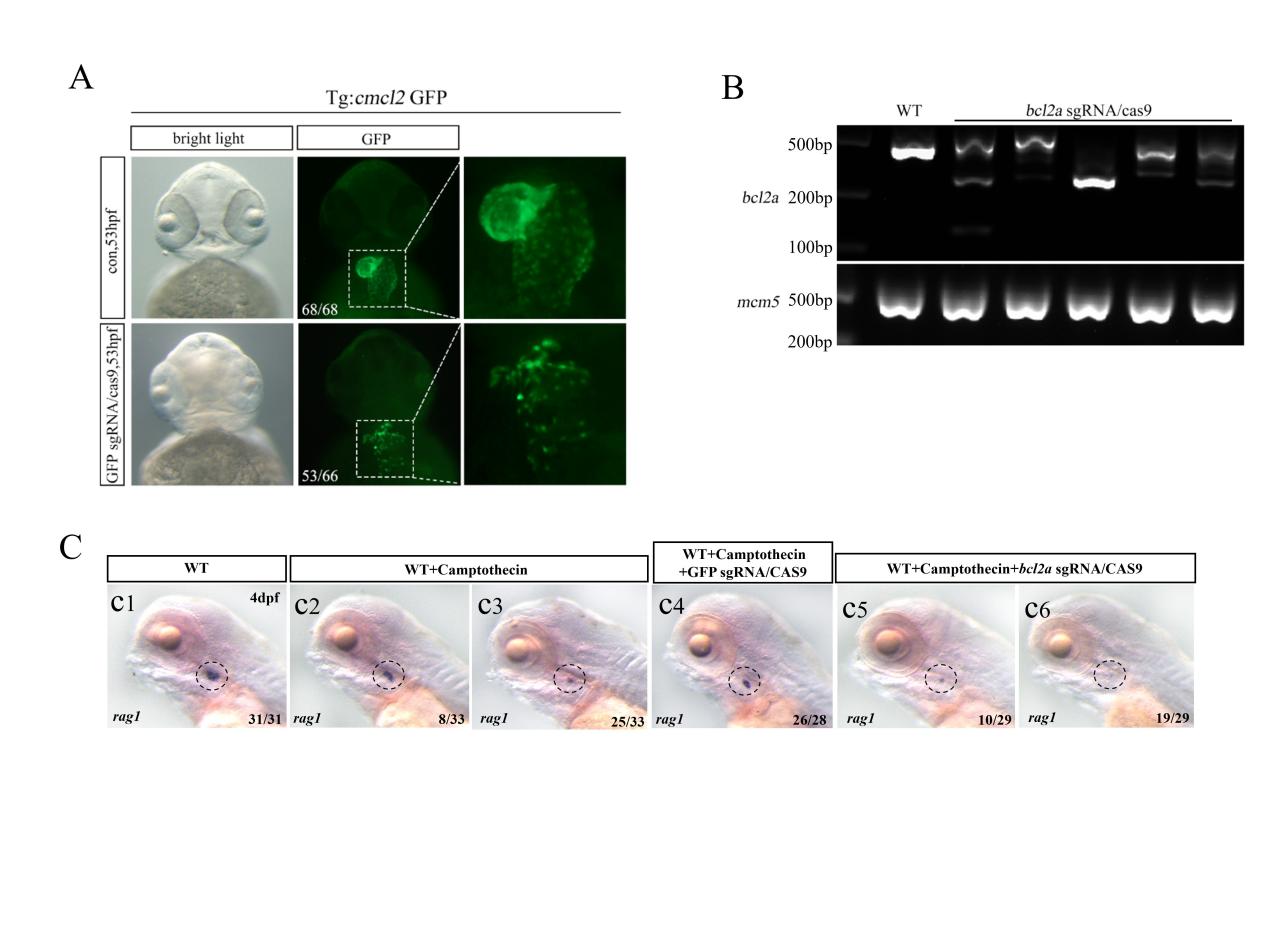
Fig. S15 bcl2a loss of function enhanced T lymphocytes phenotype in embryos treated with camptothecin**

(A, B) 3 sgRNAs targeting GFP were synthesized and co-injected with Cas9 protein into the *Tg(cmlc2:GFP)* embryos at one-cell stage, the genome was edited and the GFP expression was greatly down-regulated in the heart (A), indicating the gene editing system works well; and 4 sgRNAs targeting *bcl2a* were co-injected with Cas9 protein into the embryos, most of genome was edited (B). (C) At 4dpf, comparing with that of controls, the expression of *rag1* was decreased in embryos treated with camptothecin (c1-c3). When the embryos were treated with camptothecin, comparing to injection with GFP sgRNAs/Cas9 complex (c4), injection with *bcl2a* sgRNAs/Cas9 enhanced the T lymphocytes developmental defect (c5-6).

**
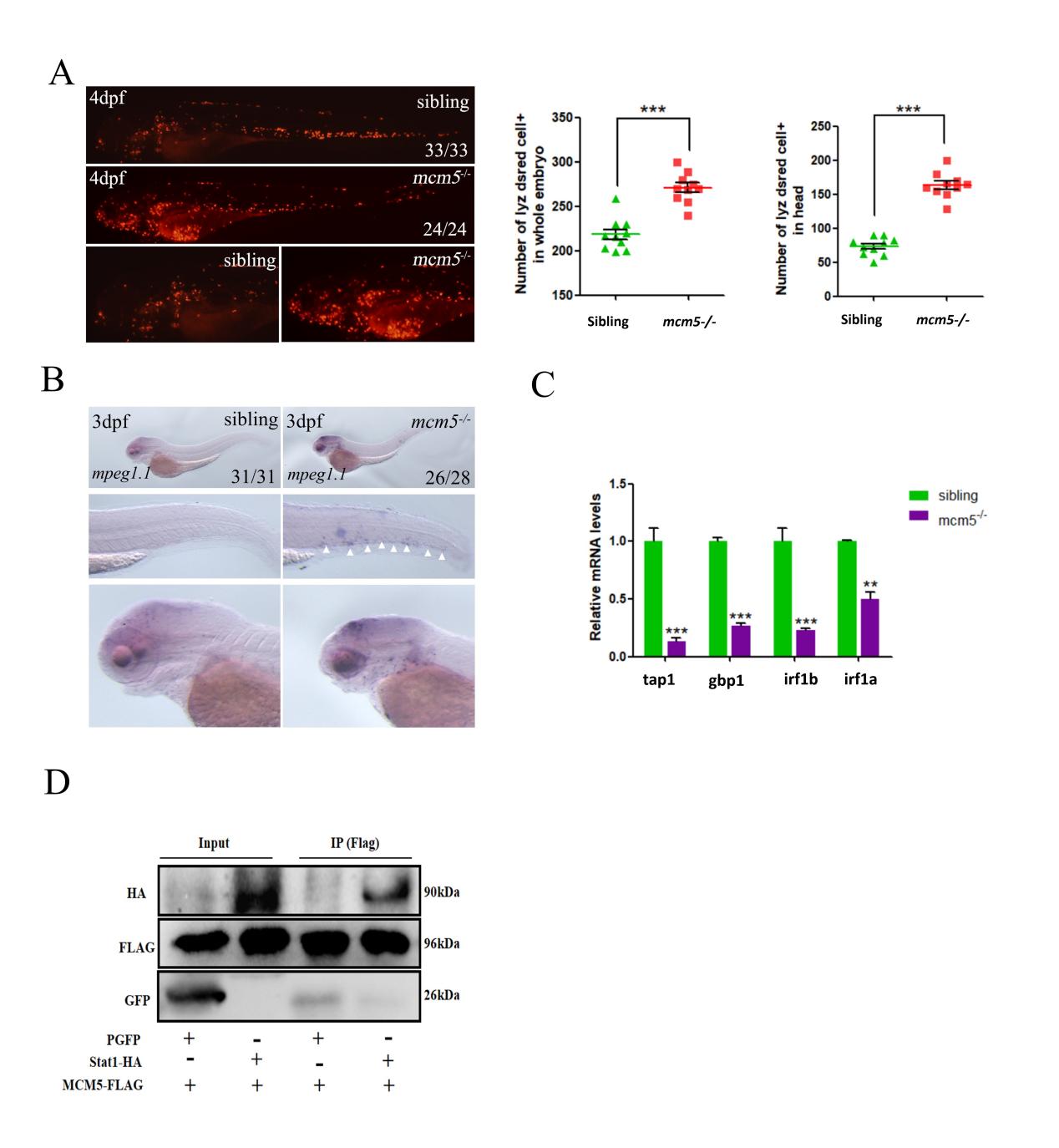
**

**Fig. S16 The activity of Stat1a signaling was downregulated in *mcm5^-/-^* embryos.**

1. The neutrophil in siblings and *mcm5^-/-^* embryos was showed in *Tg(lyz:desRed)* transgenic line. Comparing to siblings (n=9), the number of total neutrophil in *mcm5^-/-^* embryos was increased (n=10), especially in head region of *mcm5^-/-^* embryos (A, n=9). (B) *In situ* staining for probe macrophage marker *mpeg1.1* at 3dpf. Comparing to siblings (n=31), in *mcm5^-/-^* embryos, the expression of *mpeg1.1* was increased in head and tail region (n=28, 26/28). (C) The four downstream gene of Stat1, the expression of *tap1*, *gbp1*, *irf1b* and *irf1a* was downregulated in *mcm5^-/-^* embryos. (D) The interaction between Mcm5 and Stat1a was examined using CoIP experiment. The GFP was used as negative control. For a and c, the data were presented as means ± SD; The P values (t-test; two-tailed); “**” P < 0.01, “***” P < 0.001.

**
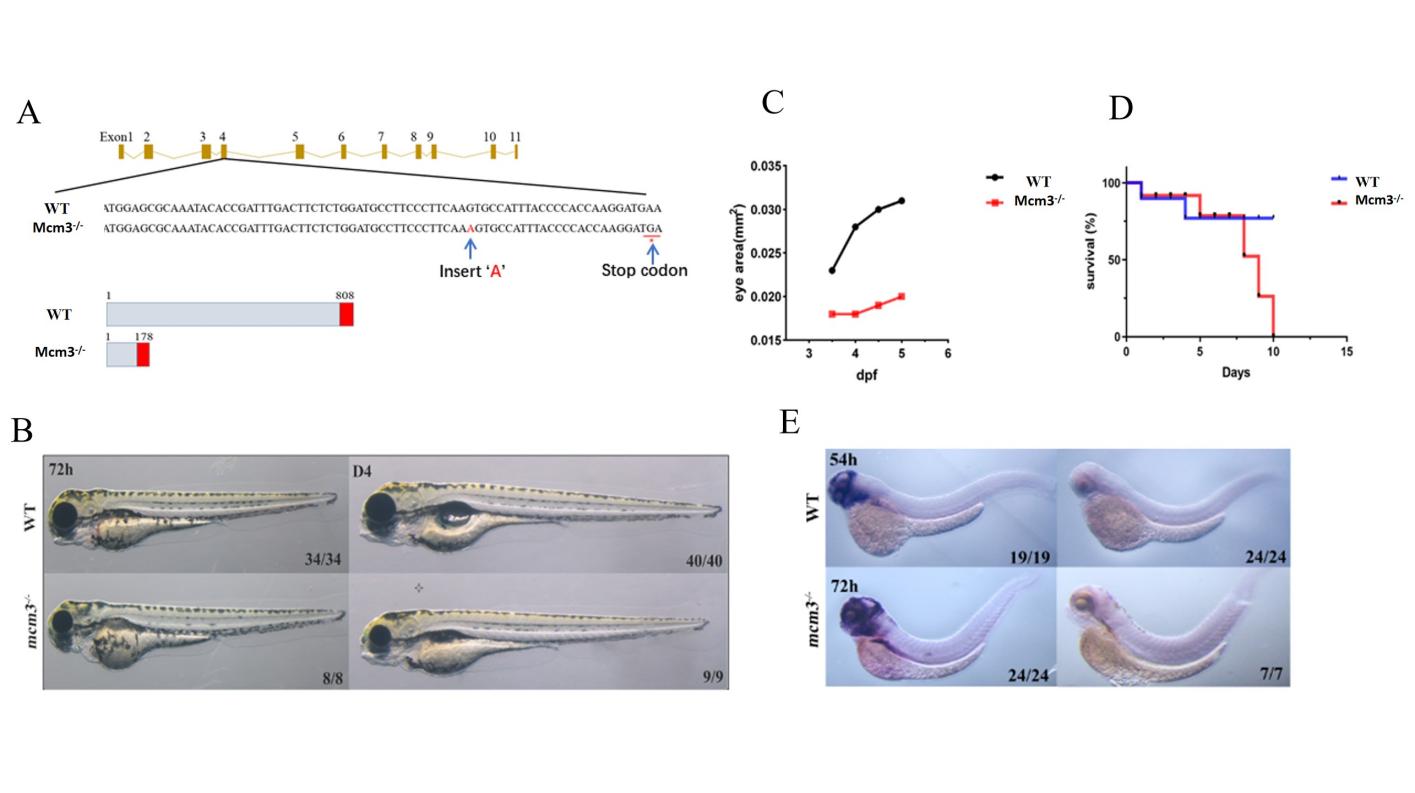
**

**Fig. S17 Generation of *mcm3* mutant**

(A) In the exon4 of *mcm3* gene, additional ‘A’ was inserted, which result the stop codon ‘TGA’. As result, in the mutant *mcm3* gene, the premature protein (only 177 AA) was produced. (B) Comparing with the wild type embryos, *mcm3* homozygotes displayed mild shorter and smaller eyes from 3dpf. (C) The size difference of the eyes in wild type embryos and mutant embryos. (D) The survival ratio of wild type embryos and mutants in early development. (E) At 54hpf and 72hpf, the expression of *mcm3* was greatly down-regulated.

**
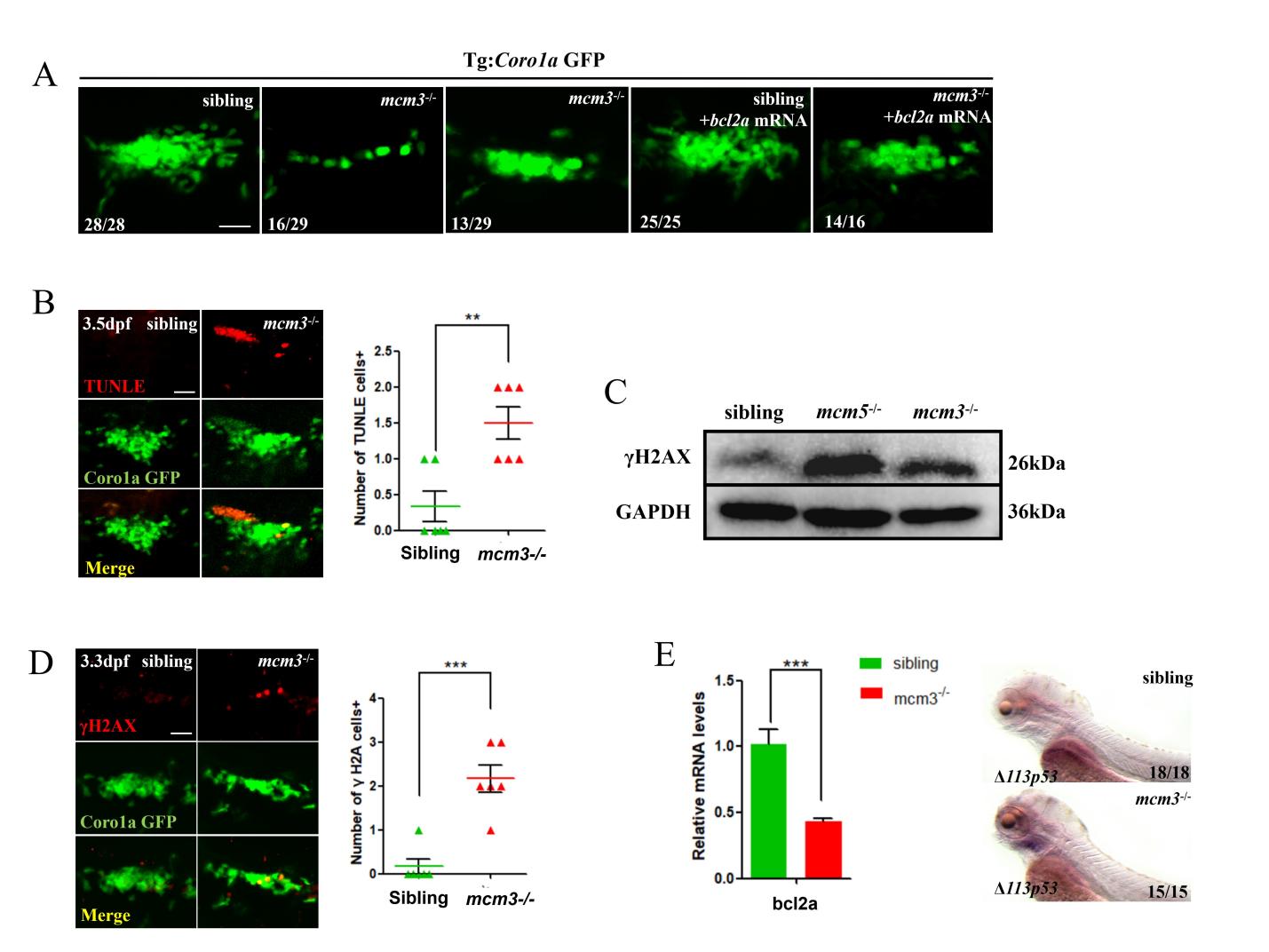
Fig. S18 T lymphocytes development in *mcm3^-/-^* embryos**

(A) In *mcm3^-/-^* embryos, *coro1a*:GFP cells was decreased，but was not as strong as that in *mcm5^-/-^* embryos. Injection of *bcl2a* mRNA partially rescued T cell developmental defect. (B) At 3.5dpf, Tunel labeled *coro1a*:GFP cells increased in *mcm3^-/-^* embryos (n=6). (C) Protein level of γH2A was increased in *mcm3^-/-^* embryos and *mcm5^-/-^* embryos. (D) In thymus region, γH2AX labeled *coro1a*:GFP cells was increased (n=6). (E) The transcription of *Δ113p53* mRNA was upregulated in *mcm3^-/-^* embryos. *Δ113p53* was upregulated in thymus and the around region. Scare bars, 40μm. For B and D, the data were presented as means ± SD; The P values (t-test; two-tailed); “*” P < 0.05,“**” P < 0.01, “***” P < 0.001.

**
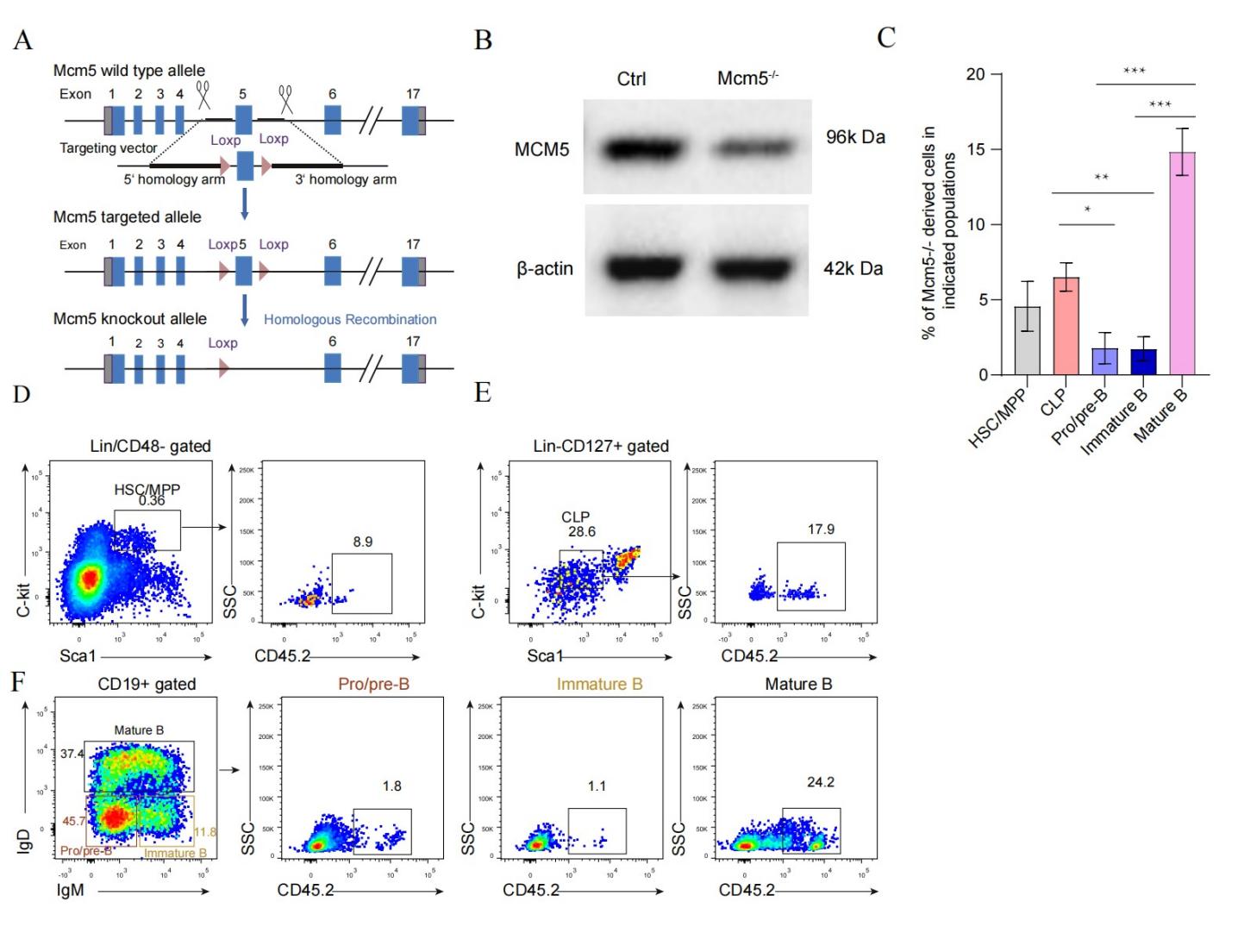
Fig. S19 Pro/pre-B and immature B cells are sensitive to Mcm5 knockout.**

(A) The method to generate Mcm5 knockout using Cre/loxp system. (B) Comparing the protein level between control mouse bone marrow and experimental mouse bone marrow using western blot. To the control mouse, WT (CD45.1) mouse bone marrow was used to perform western blot (left lane). To the experimental mouse, the Mcm5f/f;Mx1-Cre (CD45.2) and WT (CD45.1) mouse bone marrow were transplanted, 4 weeks after transplantation, 200 µg of pI-pC was injected intraperitoneally into recipients every other day for 3 doses. At 1.5 weeks after treatment with pI-pC, the bone marrow was used to perform western blot. Because in the transplanted bone marrow, the WT cell still express MCM5 after poly:IC treatment, the total MCM5 level was just downregulated (right lane). (C) Statistical analysis of Mcm5^-/-^ derived cells in HSCs/MPPs, CLPs, Pro/pre-B, Immature B and mature B cells in bone marrow. N=4, Data in (C) are represented as means ± SEM. * p<0.05, ** p<0.001, *** p<0.0001. (D-F) Flow cytometry analysis of Mcm5 knockout cells in hematopoietic stem cells/multi-potential progenitors (HSCs/MPPs), common lymphocyte progenitors (CLPs), Pro/pre-B, Immature B and mature B cells.


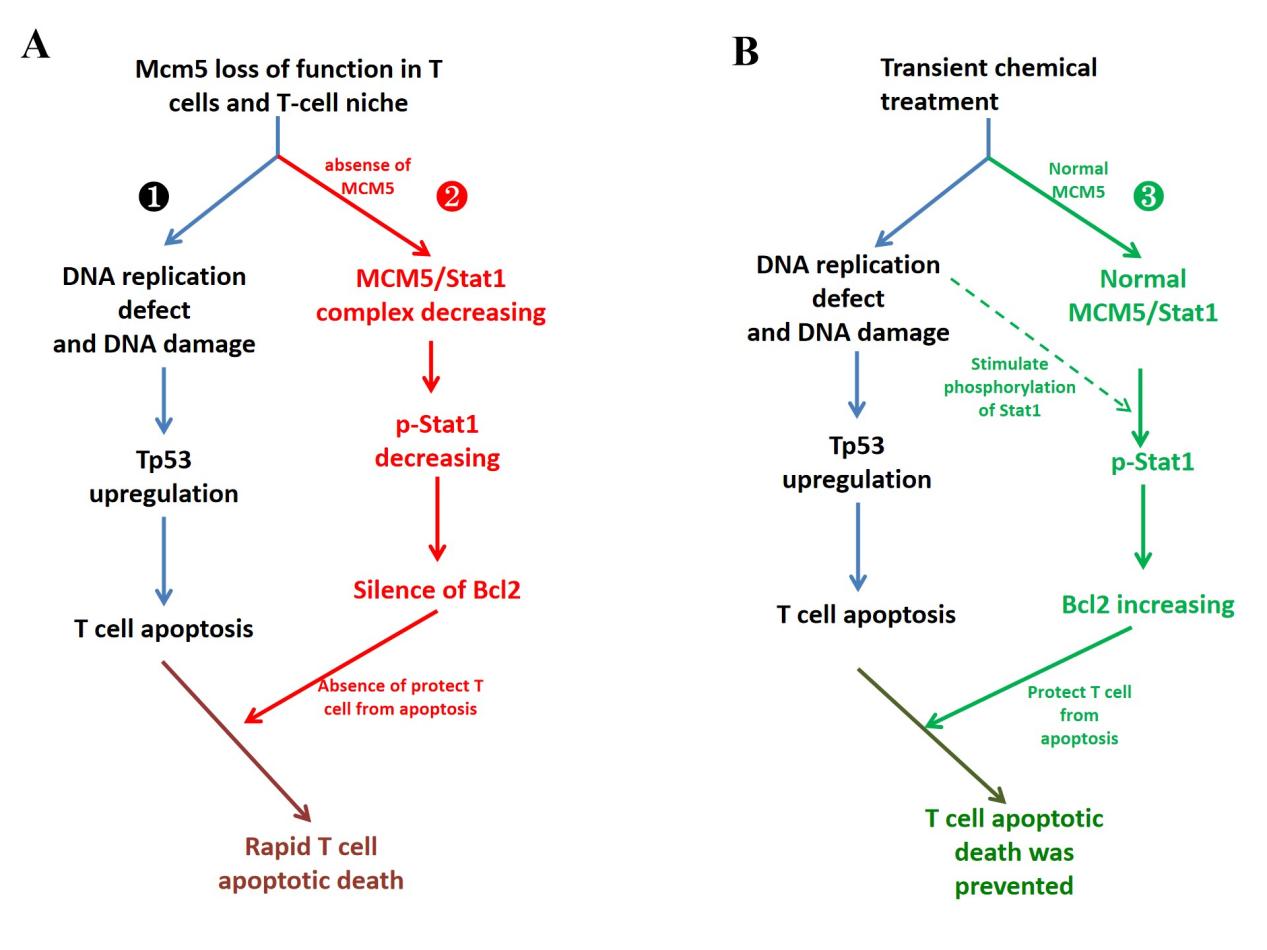


**Fig. S20 Model of how *mcm5* loss of function facilitates p53-dependent apoptosis in immature T cells**

(A) In *mcm5* mutants, *mcm5* loss of function leads to DNA damage and the sequentialupregulation of Tp53 signaling, which results in T-cell apoptosis (A, pathway 1).In addition, *mcm5* loss of function decreases the level of p-Stat without increasing the transcription of *bcl2*, which accelerates T-cell apoptosis (A, pathway 2). In contrast, under transient DNA replication stress, the normal levels of MCM5 and the MCM5/Stat1 complex increase the level of *bcl2a*to protect T cells from apoptosis (B, pathway 3).

Table S1.

Primers for RT-qPCR

| **gene name** | **Forward** | **Reverse** |
| --- | --- | --- |
| *beta actin* | 5′-CCCAGACATCAGGGAGTG-3′ | 5′-TCTCTGTTGGCTTTGGGATT-3′ |
| *P53* | 5′-GAGTTCGCGGAGCTCTGG-3′ | 5′-CCTAAATCCATGATCGCCGGG-3′ |
| *Δ113p53* | 5′-GAAGTCCGAGCATGTGGCTG-3′ | 5′-CCCCATGCAGCTGCTATTGC-3′ |
| *p21* | 5′-CGCAAACAGACCAACATCAC-3′ | 5′-CGGAATAAACGGTGTCGTCT-3′ |
| *mdm2* | 5′-CTCGCAGTGAGGGCAGTGAAG-3′ | 5′-TCTAGGCACGTAGCGGGAAGG-3′ |
| *ccng1* | 5′-GACCAGGTGACCGAAACGGG-3′ | 5′-CAGCCAGGAATCGATCTAGGAG-3′ |
| *bcl2a* | 5′-TGGCGTCCCAGGTAGATAAT-3′ | 5′-ACCGTACATCTCCACGAAGG-3′ |
| *gbp1* | 5′-CCAACATGTCTAAACCCACAATG-3′ | 5′-GGACACACCACATCCAGATACC-3′ |
| *tap1* | 5′-GTGCTGGAGATGAAGGATAAGATG-3′ | 5′-GACTGGATAGAGAAGACCGTGG-3′ |
| *irf1a* | 5′-GACATGCATCAGGGGCGTC-3′ | 5′-GAGAGCGCATCTGAAGTTCG-3′ |
| *irf1b* | 5′-CATGCCCGTGTCCAGAATG-3′ | 5′-GCGAGTTCATTGCACAGCG-3′ |

**Table S2.**

Primers for Plasmids construction

| **Primer name** | **sequence** |
| --- | --- |
| PCS^2+^_F | 5′-CTCGAGCCTCTAGAACTATAGTG-3′ |
| PCS^2+^_R | 5′-TGGTGTTTTCAAAGCAACGATATCG-3′ |
| *Δ113p53_*F | 5′-CATCGATATCGTTGCTTTGAAAACACCAGTTCAAATGGTGGTGGACGTTG-3′ |
| *Δ113p53_R* | 5′-CGACTCACTATAGTTCTAGAGGCTCGAGCCCATCACCTTAATCAGAGTCGC-3′ |
| *bcl2a_*F | 5′-GCTTTGAAAACACCACGTGATTTAAAACCATGGCTAACG-3 |
| *bcl2a*_R | 5′-TTCTAGAGGCTCGAGGAGGCTGTCACTTCTGAGC-3′ |
| pcDNA3.1^_^F | 5′-CCAGTGTGGTGGAATTCTGC-3′ |
| pcDNA3.1^+^_R | 5′-GGATCCGAGCTCGGTACC-3′ |
| *mcm5*_F | 5′-ACCGAGCTCGGATCCATGTCGGGATTTGATGATCCAGG-3′ |
| *mcm5*_R | 5′-ATTCCACCACACTGGCTATTTGACCCTGTAAAGCACCTTC-3′ |
| *stat1a*_F | 5′-ACCGAGCTCGGATCCGTCGCAGCAATGACTCAGTGG-3′ |
| *stat1a*_R | 5′-ATTCCACCACACTGGCGAGTCACTCGCGTTTTCTATGTC-3′ |

Table S3.

MO sequence

| **MO name** | **sequence** |
| --- | --- |
| *mcm5*MO | 5′-ATAGTTTCGATAAGTGCTGTC GATG-3′ |
| *p53* MO | 5′-GCGCCATTGCTTTGCAAGAATTG-3′ |
| *stat1a* MO | 5′-GCTGAAGCTCCAACCACTGAGTCAT-3′ |
| control MO | 5′-CCTCTTACCTCAGTTACAATTTATA-3′ |
