# Supplementary material for: Mcm5 mutation leads to silencing of Stat1-bcl2 which accelerating apoptosis of immature T lymphocytes with DNA damage
Source: Cell Death Dis. 2025 Feb 10;16(1):84. doi: 10.1038/s41419-025-07392-8 (PMC11811017; doi:10.1038/s41419-025-07392-8)
Supplement: Supplementary file 2 — Western Blot Gel image (merged in one PDF file). [file 41419_2025_7392_MOESM2_ESM.pdf]

# Fig.3 C Gel Supplementary

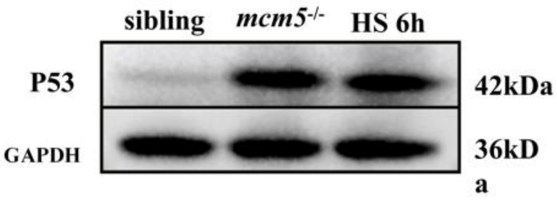


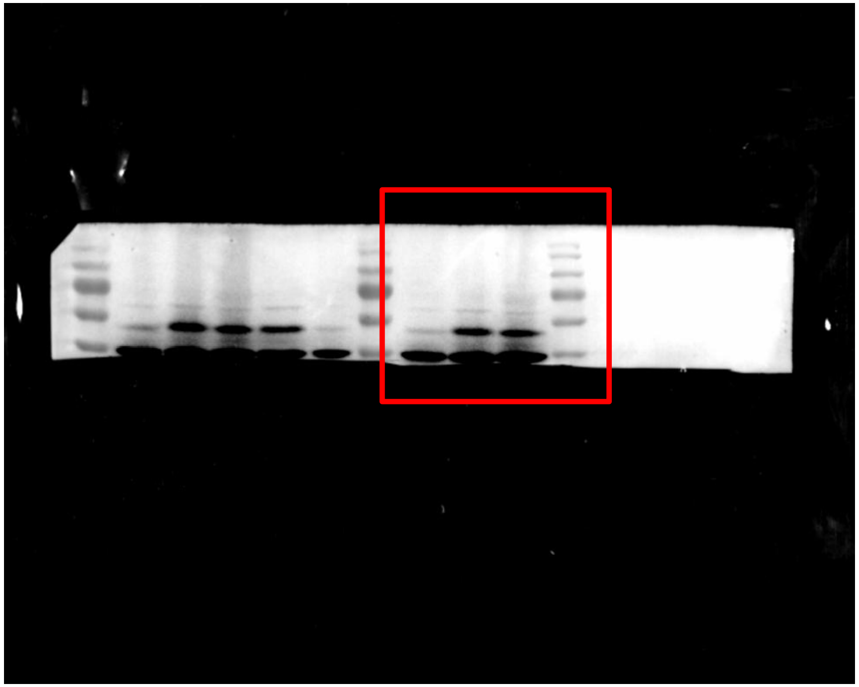

p53

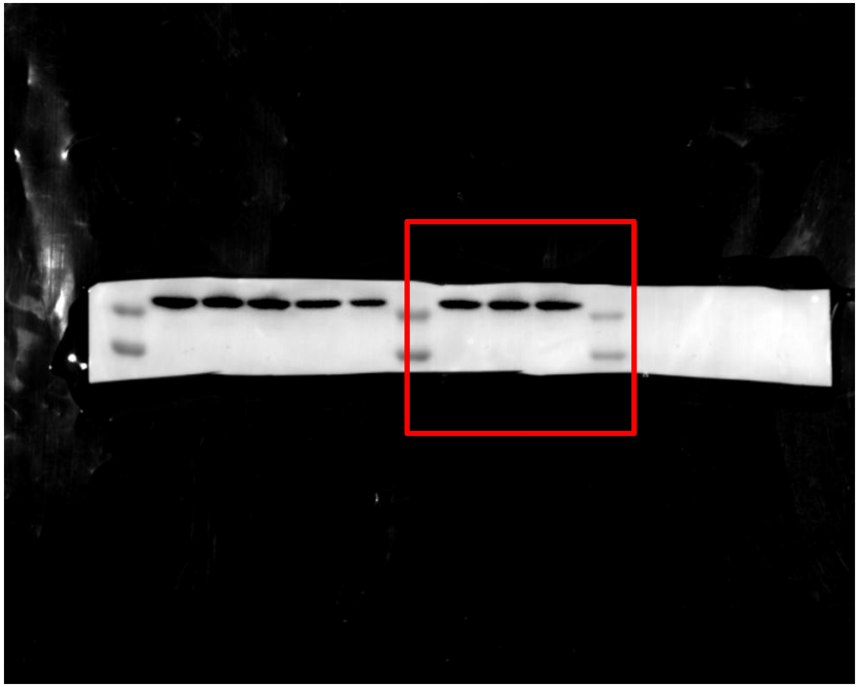

GAPDH

Fig.4 B Gel Supplementary

B

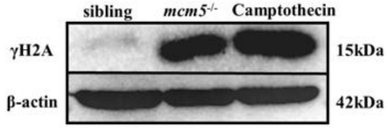


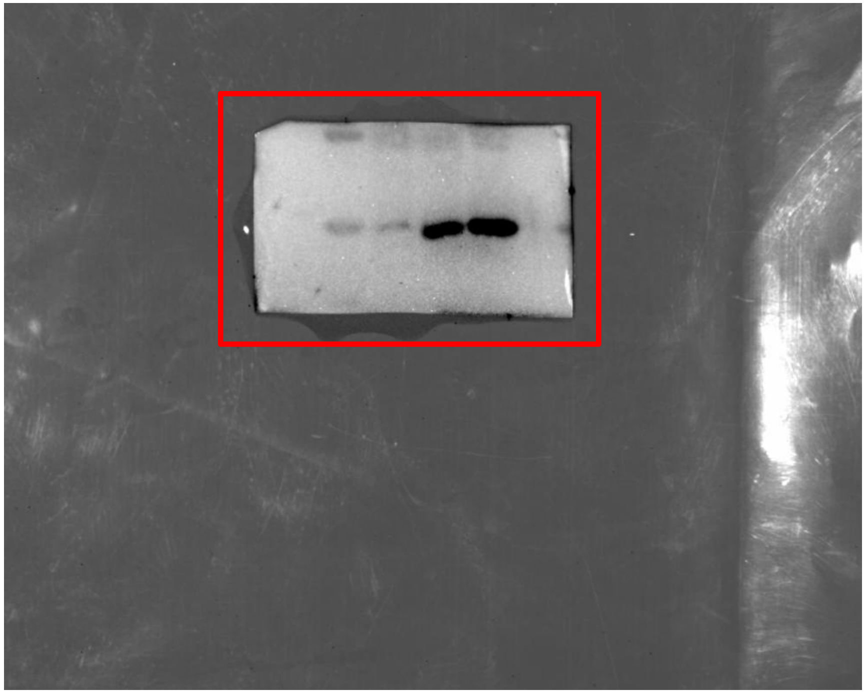

**γH2A**

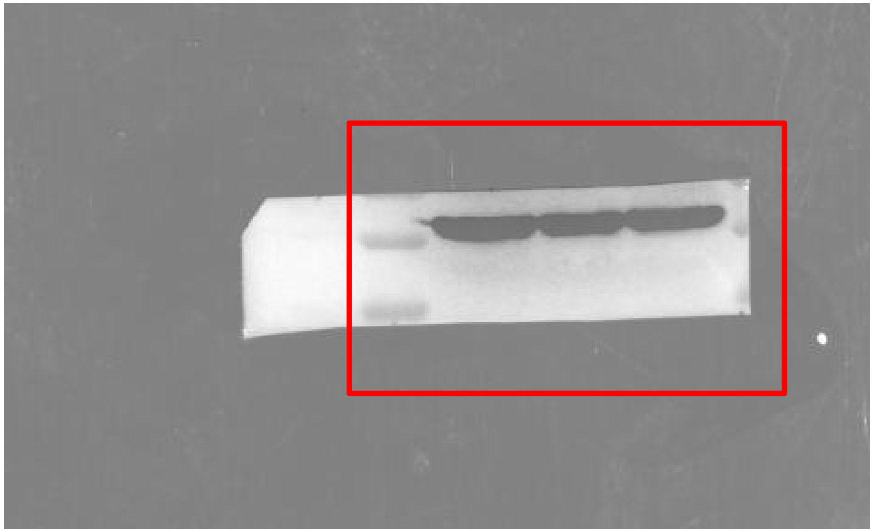

**β-actin**

Fig.5 F Gel Supplementary

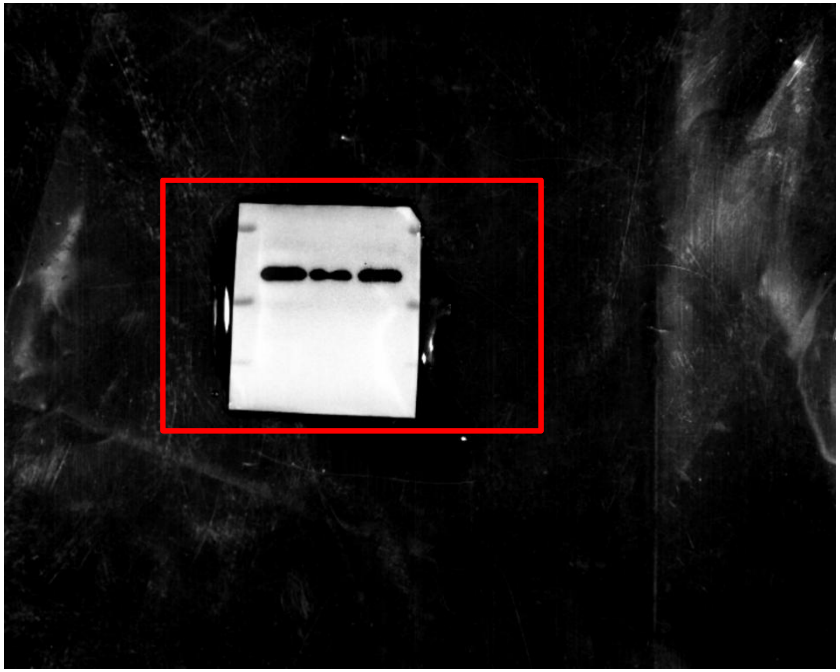

**BCL2**

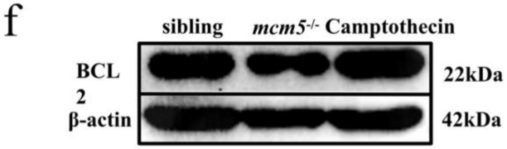


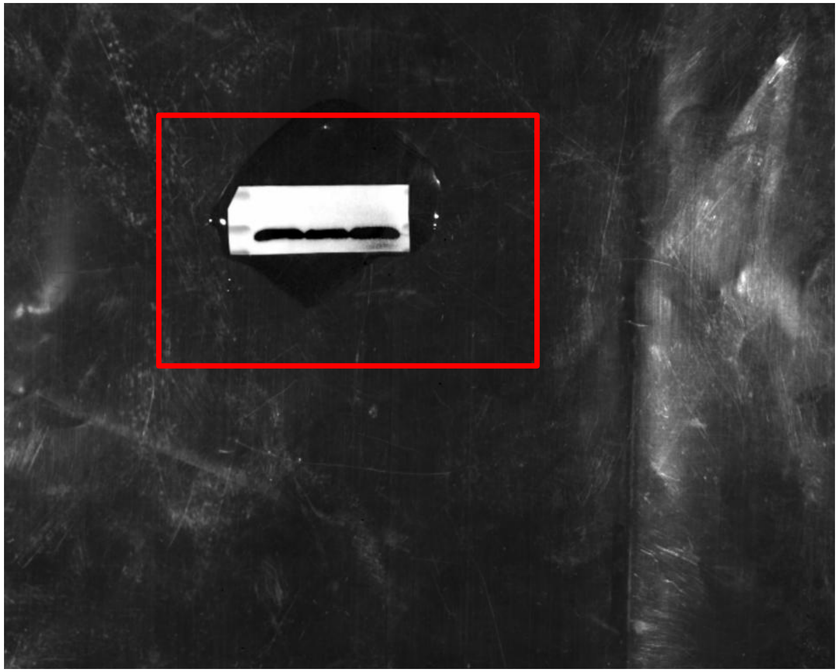

**β-actin**

# Fig.6 A Gel Supplementary 1

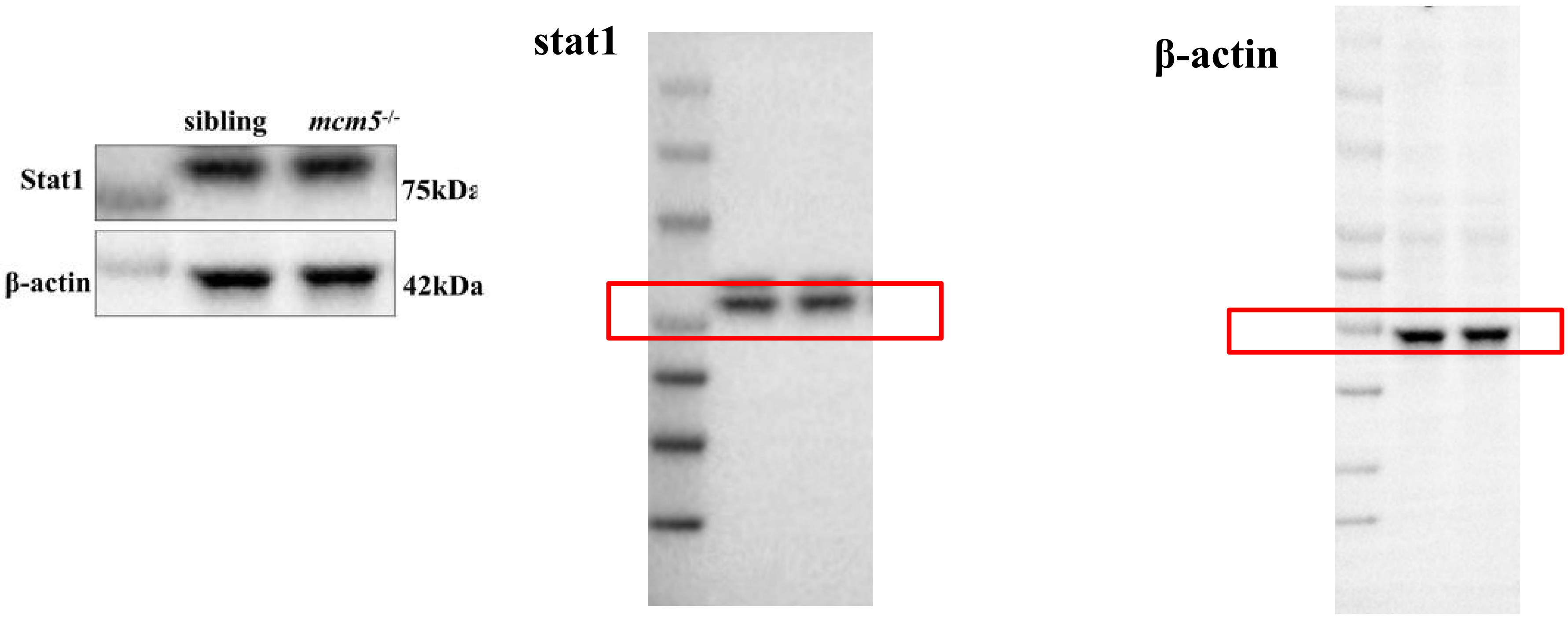

Fig.6 A Gel Supplementary 2

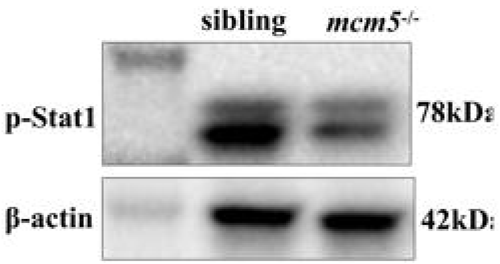


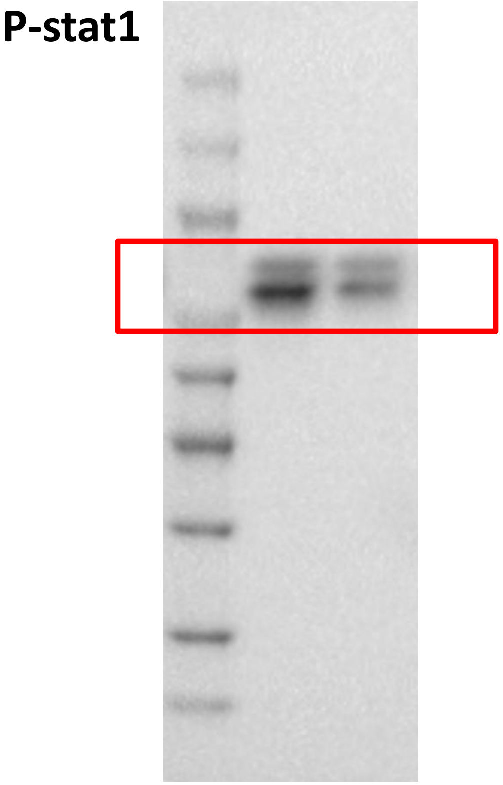


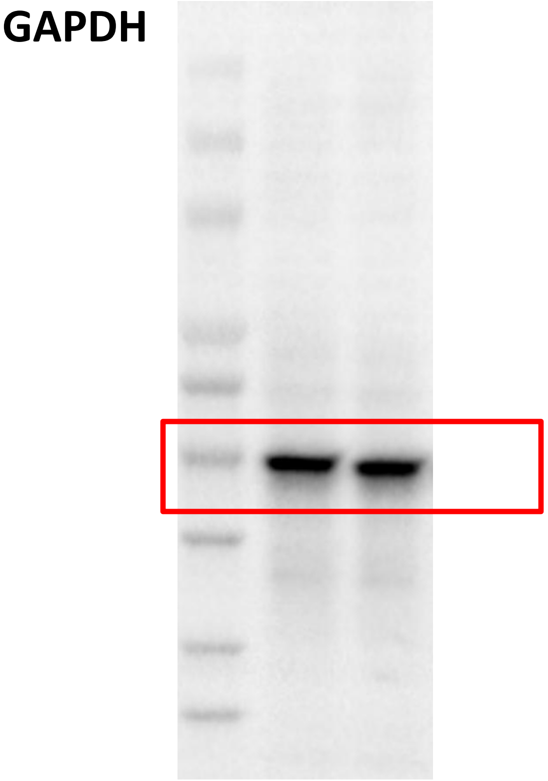

Fig.6 G Gel Supplementary

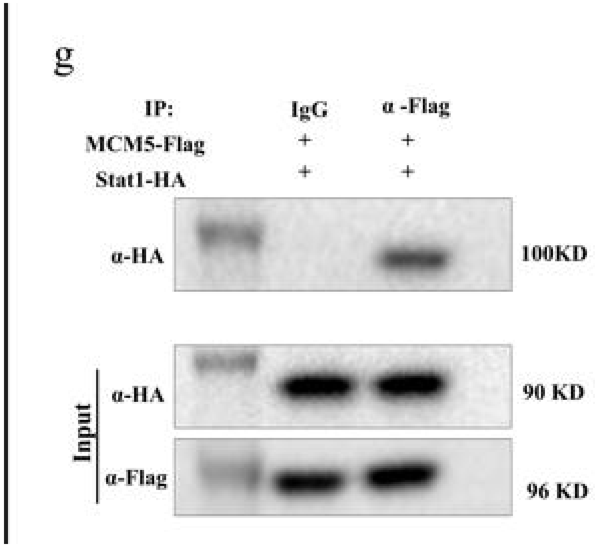


Anti-HA(IP)

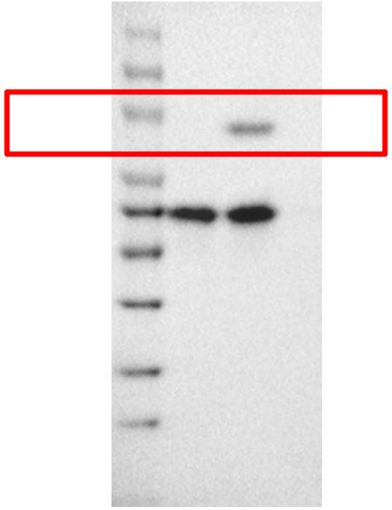

Anti-HA(in put)

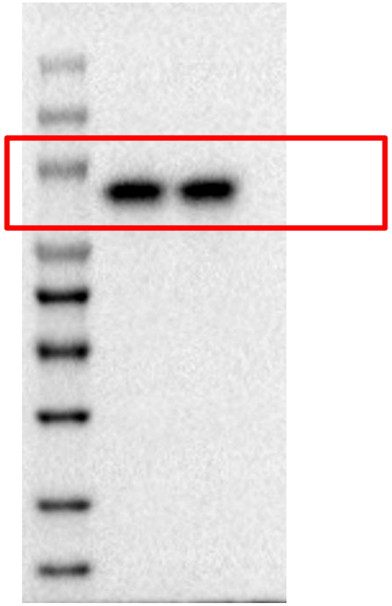

Anti-Flag(in put)

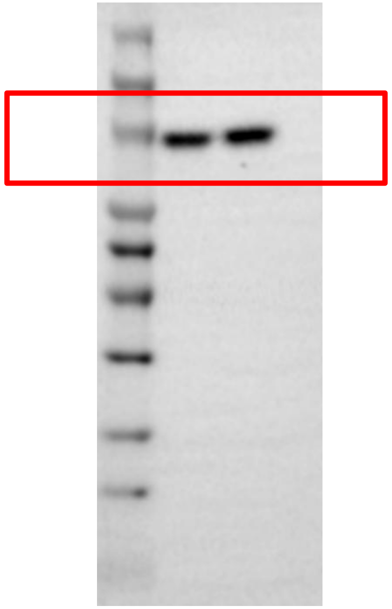

Fig.6 I Gel Supplementary

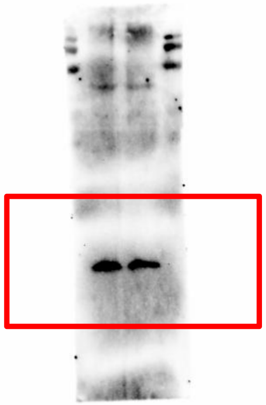

BCL2

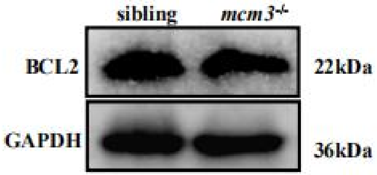


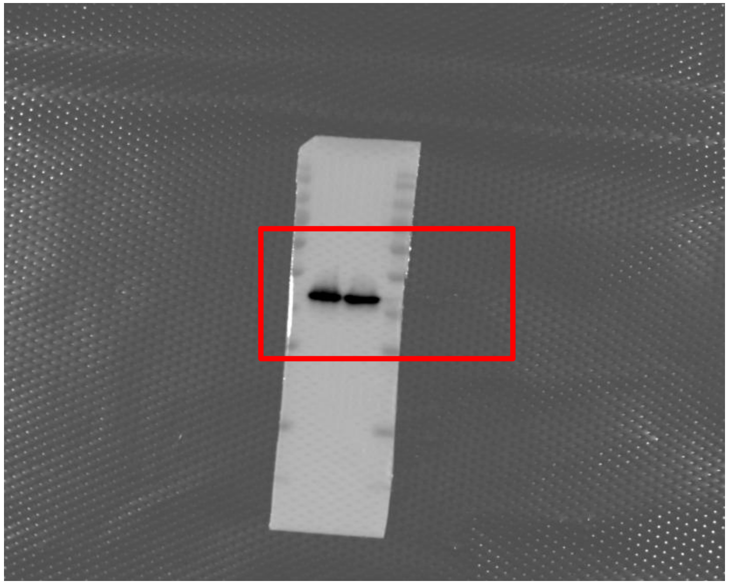

GAPDH

Fig.7 B Gel Supplementary 1

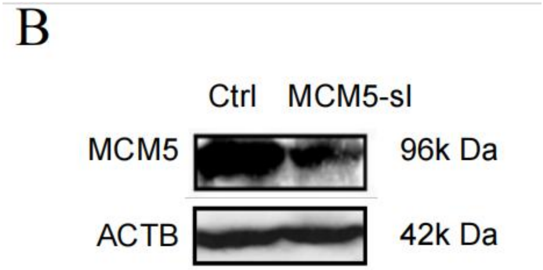


**MCM5**

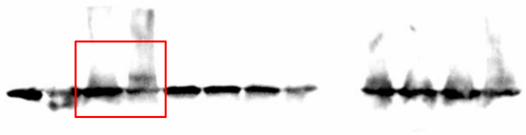

**β-actin**

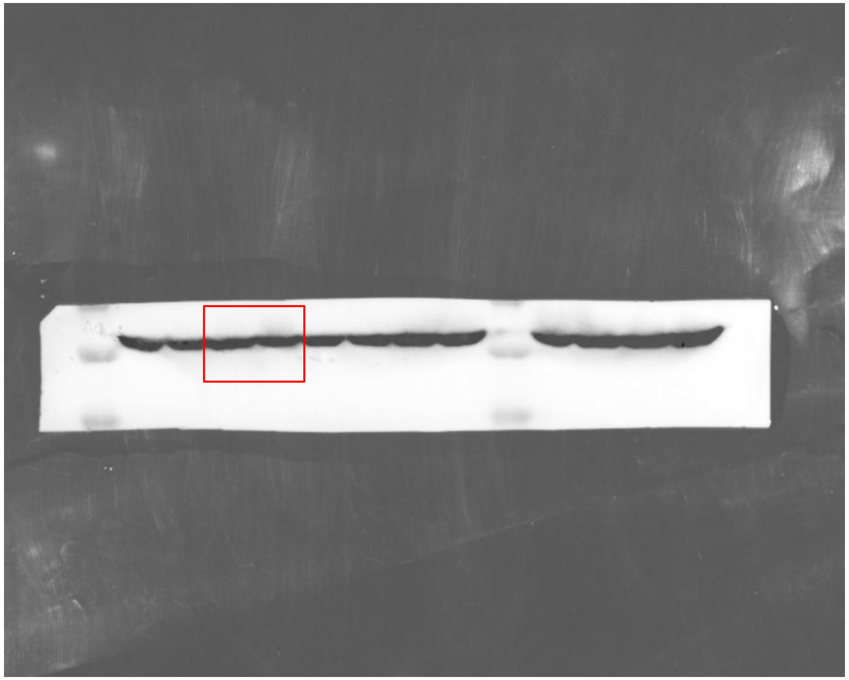

Fig.7 B   Gel Supplementary 2

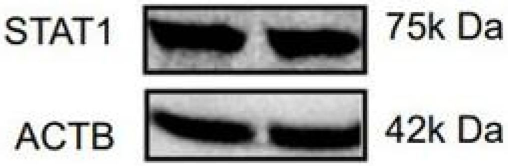


**STAT1**

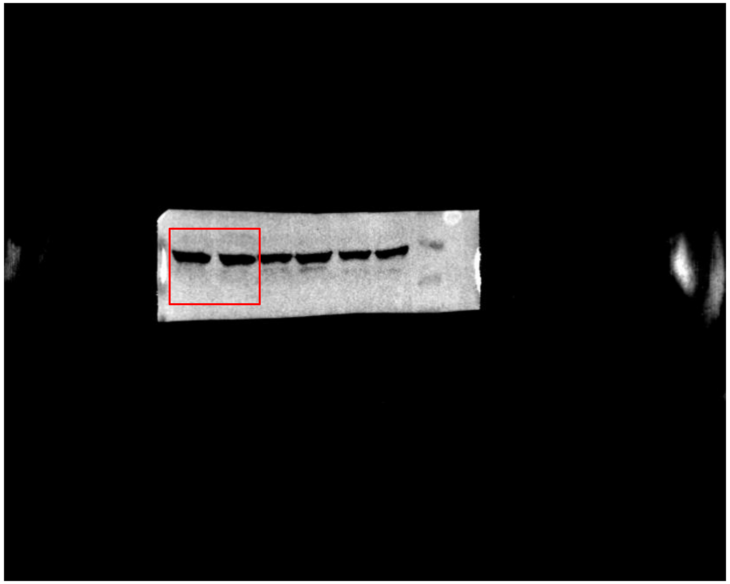

**β-actin**

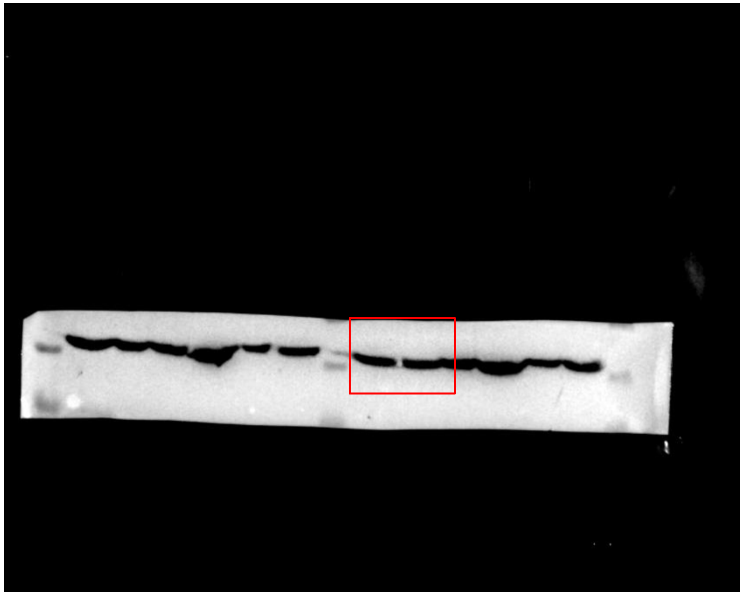

**P-STAT1**

Fig.7 B   Gel Supplementary 3

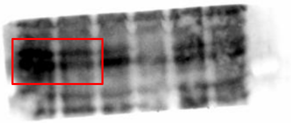

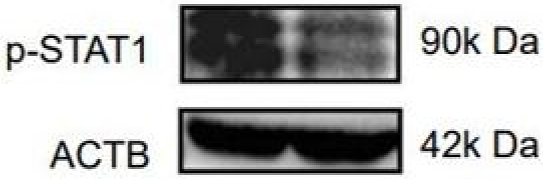


**β-actin**

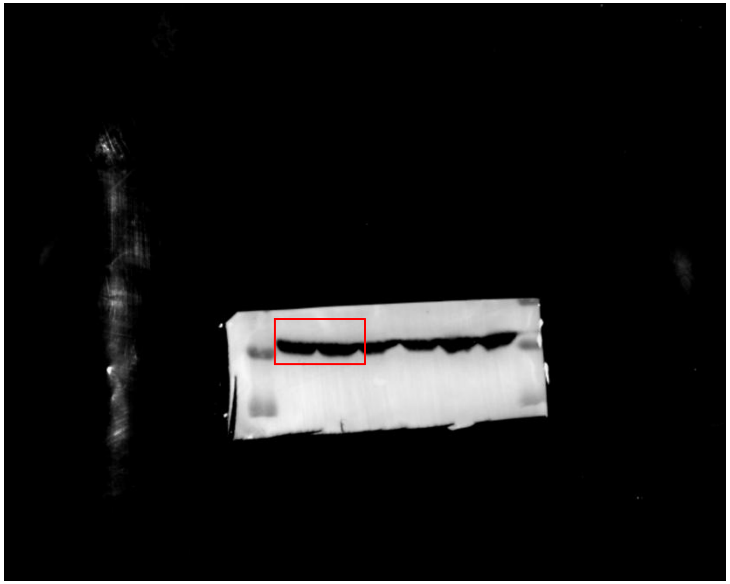

Fig. S1 B Gel Supplementary

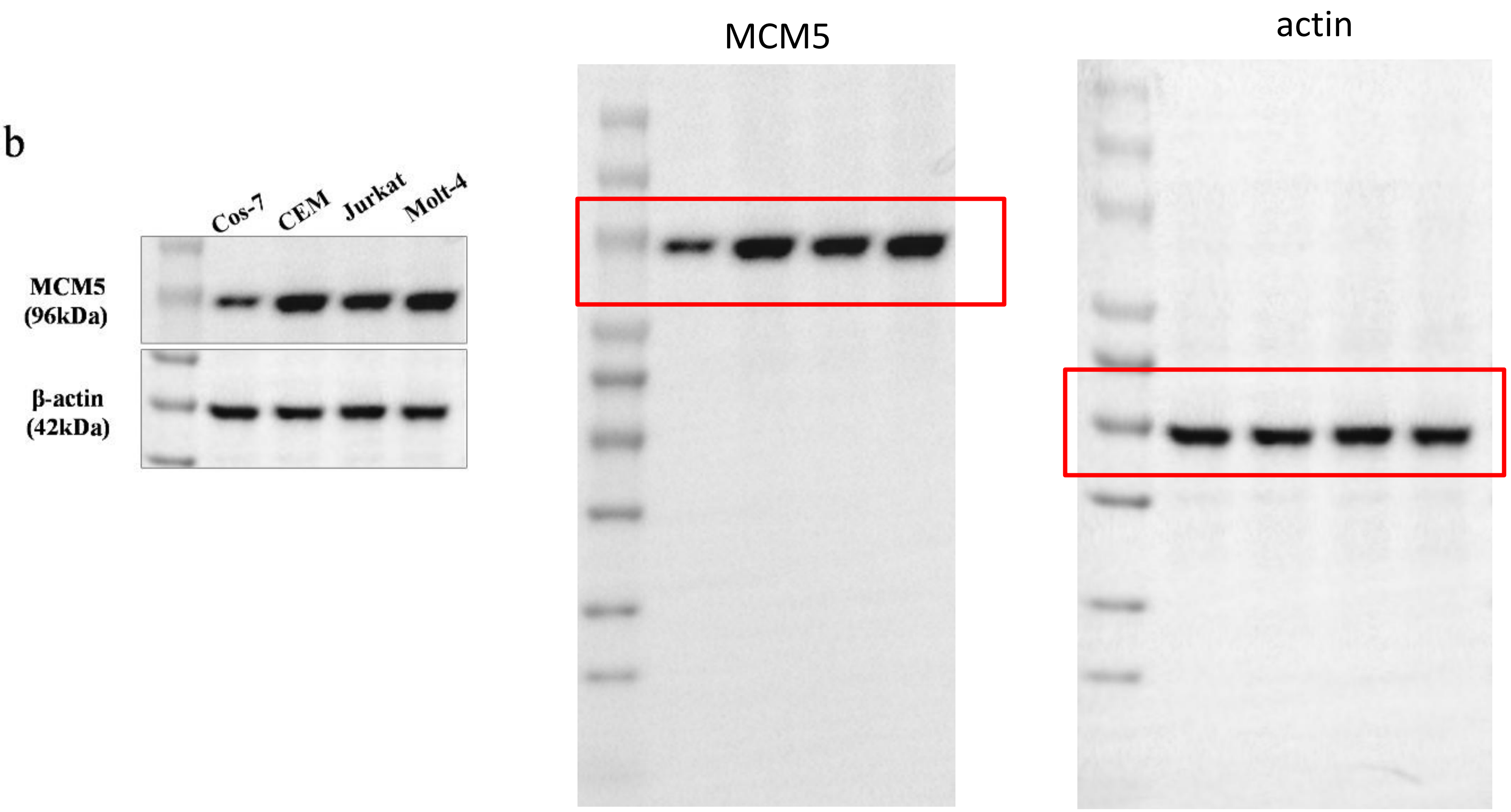

Fig. S16 D    Gel Supplementary

E

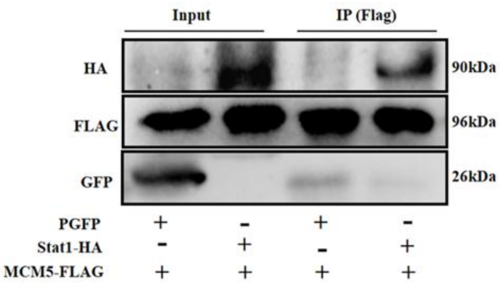


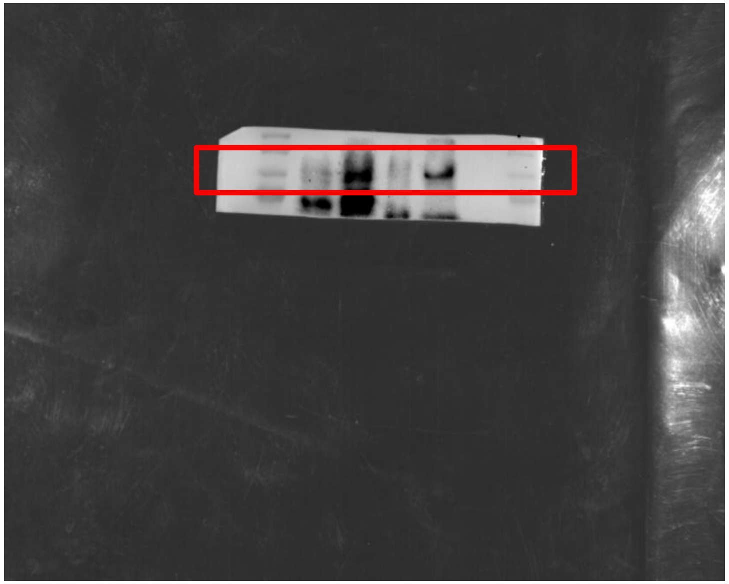

HA

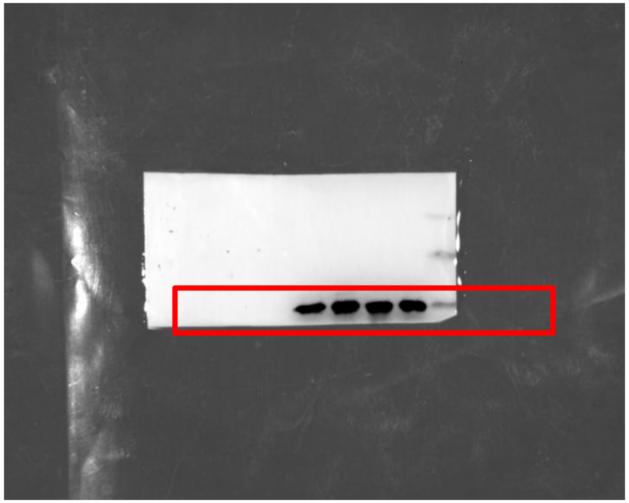

Flag

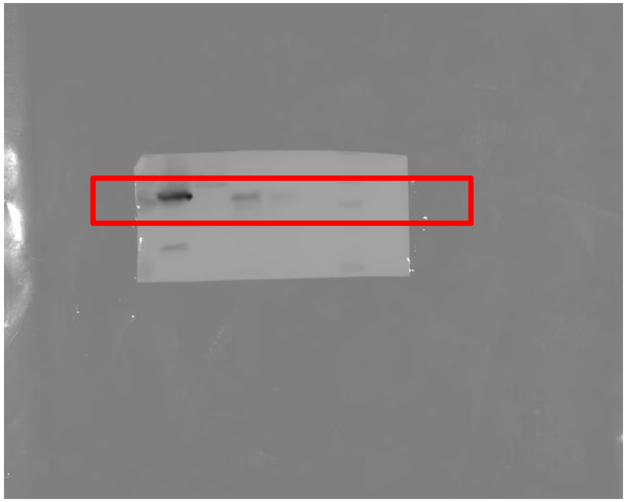

GFP

Fig.S18 C Gel Supplementary

C

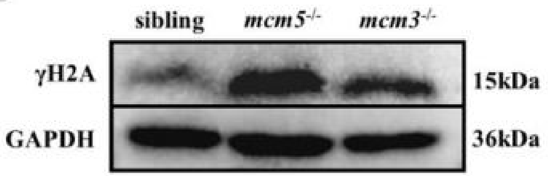


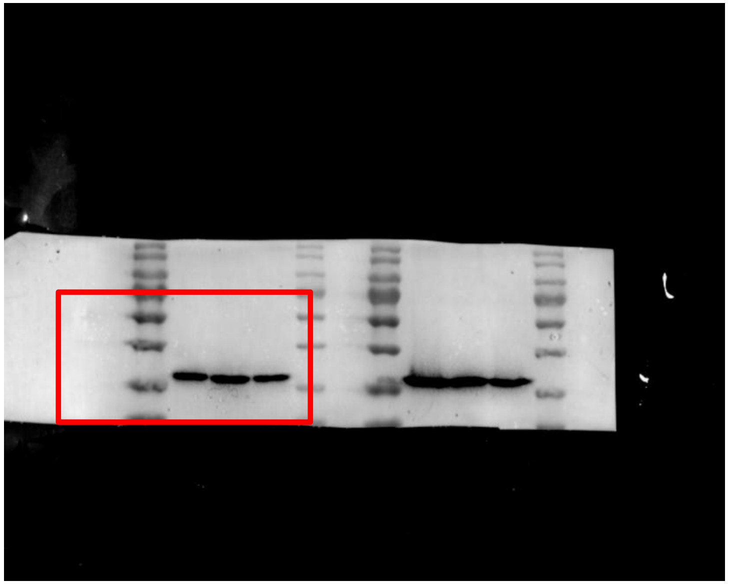

gapdh

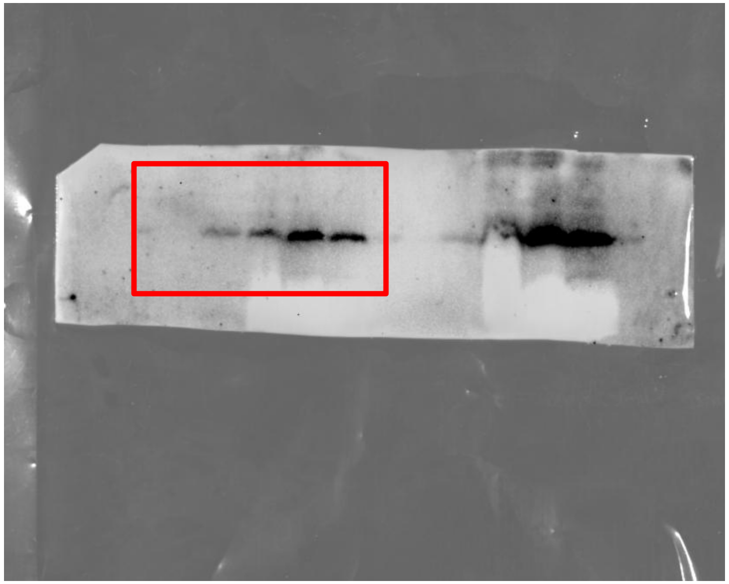

γH2A
